# A force-sensitive adhesion GPCR is required for equilibrioception

**DOI:** 10.1038/s41422-025-01075-x

**Published:** 2025-02-18

**Authors:** Zhao Yang, Shu-Hua Zhou, Qi-Yue Zhang, Zhi-Chen Song, Wen-Wen Liu, Yu Sun, Ming-Wei Wang, Xiao-Long Fu, Kong-Kai Zhu, Ying Guan, Jie-Yu Qi, Xiao-Hui Wang, Yu-Nan Sun, Yan Lu, Yu-Qi Ping, Yue-Tong Xi, Zhen-Xiao Teng, Lei Xu, Peng Xiao, Zhi-Gang Xu, Wei Xiong, Wei Qin, Wei Yang, Fan Yi, Ren-Jie Chai, Xiao Yu, Jin-Peng Sun

**Affiliations:** 1 NHC Key Laboratory of Otorhinolaryngology, Qilu Hospital of Shandong University, and New Cornerstone Science Laboratory, Department of Biochemistry and Molecular Biology, School of Basic Medical Sciences, Cheeloo College of Medicine, Shandong University, Jinan, Shandong China; 2 State Key Laboratory of Digital Medical Engineering, Department of Otolaryngology Head and Neck Surgery, Zhongda Hospital, School of Life Sciences and Technology, Advanced Institute for Life and Health Jiangsu Province High-Tech Key Laboratory for Bio-Medical Research, Southeast University, Nanjing, Jiangsu China; 3 Department of Physiology, School of Basic Medical Sciences, Cheeloo College of Medicine, Shandong University, Jinan, Shandong China; 4 Department of Otolaryngology-Head and Neck Surgery, Shandong Provincial ENT Hospital, Cheeloo College of Medicine, Shandong University, Jinan, Shandong China; 5 Department of Otorhinolaryngology, Union Hospital, Tongji Medical College, Huazhong University of Science and Technology, Wuhan, Hubei China; 6 Advanced Medical Research Institute, Cheeloo College of Medicine, Shandong University, Jinan, Shandong China; 7 Department of Clinical Laboratory, The Second Hospital, Cheeloo College of Medicine, Shandong University, Jinan, Shandong China; 8 Medical Science and Technology Innovation Center, Shandong First Medical University & Shandong Academy of Medical Sciences, Jinan, Shandong China; 9 State Key Laboratory of Vascular Homeostasis and Remodeling, Department of Physiology and Pathophysiology, School of Basic Medical Sciences, Peking University, Beijing, China; 10 Shandong Provincial Key Laboratory of Animal Cells and Developmental Biology, Shandong University School of Life Sciences, Qingdao, Shandong China; 11 Chinese Institute for Brain Research, Beijing, China; 12 School of Physics, State Key Laboratory of Crystal Materials, Shandong University, Jinan, Shandong China; 13 Department of Biophysics, and Department of Neurology of the Fourth Affiliated Hospital, Zhejiang University School of Medicine, Hangzhou, Zhejiang China; 14 Department of Pharmacology, School of Basic Medical Sciences, Shandong University, Jinan, Shandong China

**Keywords:** Ion channel signalling, Calcium signalling

## Abstract

Equilibrioception (sensing of balance) is essential for mammals to perceive and navigate the three-dimensional world. A rapid mechanoelectrical transduction (MET) response in vestibular hair cells is crucial for detecting position and motion. Here, we identify the G protein-coupled receptor (GPCR) LPHN2/ADGRL2, expressed on the apical membrane of utricular hair cells, as essential for maintaining normal balance. Loss of LPHN2 specifically in hair cells impaired both balance behavior and the MET response in mice. Functional analyses using hair-cell-specific *Lphn2*-knockout mice and an LPHN2-specific inhibitor suggest that LPHN2 regulates tip-link-independent MET currents at the apical surface of utricular hair cells. Mechanistic studies in a heterologous system show that LPHN2 converts force stimuli into increased open probability of transmembrane channel-like protein 1 (TMC1). LPHN2-mediated force sensation triggers glutamate release and calcium signaling in utricular hair cells. Importantly, reintroducing LPHN2 into the hair cells of *Lphn2*-deficient mice restores vestibular function and MET response. Our data reveal that a mechanosensitive GPCR is required for equilibrioception.

## Introduction

The sense of balance and motion enables us to perceive and navigate the three-dimensional world, making it crucial to our interactions with the environment. Positional or motional information is primarily perceived by hair cells located in the sensory epithelium of vestibular end organs, which include two perpendicularly arranged otolith organs, the utricle and saccule, and three semicircular canals.^1,2^ Extensive evidence has shown that vestibular hair cells (VHCs) transmit balance information about head motion or tilt into electrical signals through opening of mechanically-gated ion channels, which is termed mechanoelectrical transduction (MET).^3,4^ Extremely rapid channel gating on a microsecond timescale occurs following hair bundle displacement in response to external forces, likely through tip links, as illustrated by the seminal work of the Hudspeth group and many others.^3–6^ Specifically, the MET channels in bullfrog saccular hair cells can open within ~40 μs when mechanical stimuli are presented to the cells.^6^ Most recently, two candidate channels, transmembrane channel-like proteins (TMCs) 1 and 2, which are expressed at the tip of stereocilia, have been suggested to be the pore-forming subunits of MET channels. The TMC1/2 forms a complex with additional components, such as TMIE, LHFPL5, CIB2/3 and the tip link, to sense and transduce mechanical forces.^7–10^

In addition to ion channels, membrane receptors belonging to the family of G protein-coupled receptors (GPCRs), which are the most common drug targets, can sense mechanical forces.^11–19^ For example, several adhesion GPCRs (aGPCRs), which have large, multidomain N-termini that enable interaction with extracellular matrices, can respond to mechanical force stimulation (Supplementary information, Fig. S1a).^16,20–24^ Ion channels and GPCRs represent two principal categories of sensory receptors in vertebrates.^25^ Notably, temperature and touch are sensed by TRP channels and Piezo channels, respectively.^26,27^ In contrast, vision and olfaction are mainly mediated by GPCRs, which convert light or odor stimuli into electrical signals through coupling to cyclic nucleotide-gated (CNG) channels.^28–31^ Both GPCR family members and ion channels participate in distinct taste sensations.^32,33^ The downstream signaling and cellular outputs differ between GPCRs and ion channels. While sensory ion channels directly mediate ion permeability, GPCRs regulate sensory signals by controlling intracellular concentrations of secondary messengers such as cAMP, cGMP, Ca^2+^.^34–37^ The generation of a second messenger requires enzyme catalysis, the speed of which is normally limited by diffusion limits.^38,39^ Therefore, despite playing important roles in the sensation of light, smell and taste, the GPCR-second messenger system is conventionally excluded from the equilibrioception process in hair cells due to its relatively slow kinetics.^6,40,41^

**Fig. 1:**
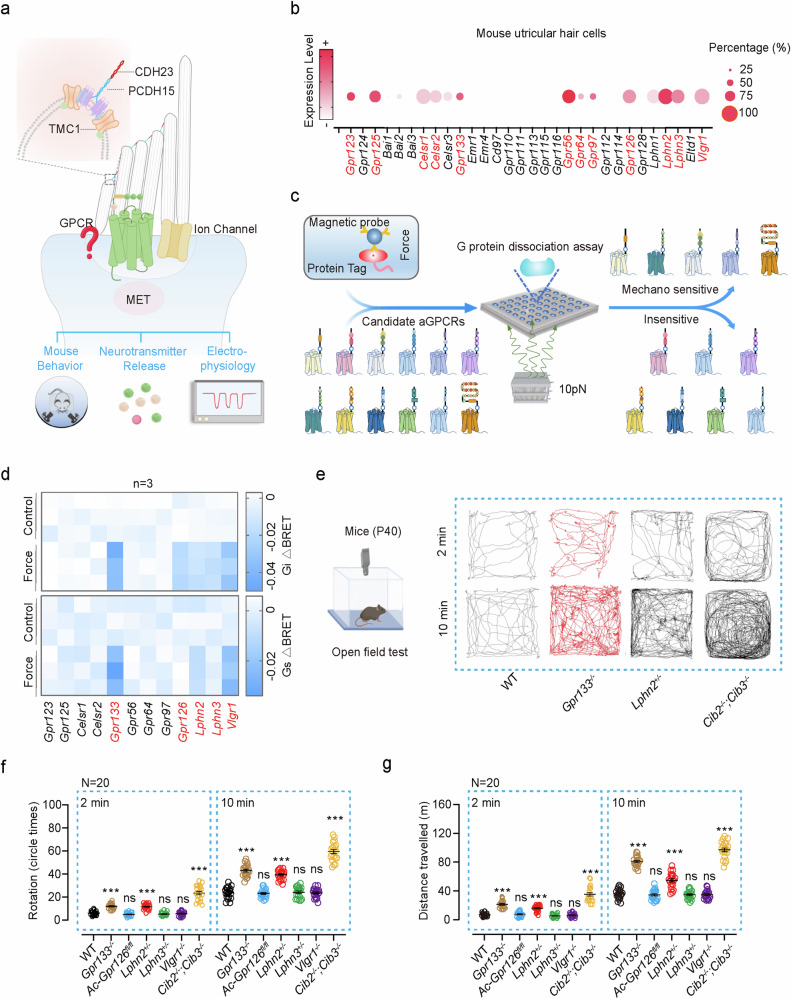
Screening of mechanosensitive aGPCRs in vestibular hair cells. **a** Schematic representation of the potential roles of ion channels and GPCRs in MET process in vestibular hair cells. Enlarged box shows a tip link, composed of PCDH15 and CDH23, and putative components of the MET channel complex at the top of one stereocilium. **b** Expression profiles of 30 aGPCR genes in mouse utricular hair cells (data from GSE71982). The intensity of the circle color indicates the average mRNA expression level of the aGPCR in utricular hair cells. The size of the circle indicates the percentage of hair cells in which expression of the aGPCR was detected (aGPCR-expressing hair cell number/total hair cell number × 100%). The 12 aGPCRs expressed in more than 20% of the utricular hair cells are highlighted. **c** Schematic representation of the strategy used to screen mechanosensitive aGPCRs. **d** Summary of the force-induced Gi3 (top panel) and Gs (bottom panel) activation downstream of 12 aGPCRs. A force of 10 pN was applied to the receptors and the Gi3 or Gs activation was measured by BRET assay, which was presented as a heatmap (*n* = 3). **e** Schematic view (left panel) and representative tracks (right panel) of WT, *Gpr133*^−*/*−^, *Lphn2*^*+/*−^ and *Cib2*^−*/*−^*;Cib3*^−*/*−^ mice in open-field tests during 2-min or 10-min tracking period. **f**, **g** Quantification of the circling (**f**) and traveling activity (**g**) of WT, *Gpr133*^−*/*−^, *Gpr133*^*+/*−^, *Atoh1*-*Cre*^+/−^;*Gpr126*^*fl/fl*^ (referred to as *Ac*-*Gpr126*^*flfl*^), *Lphn2*^*+/*−^, *Lphn3*^*+/*−^, *Vlgr1*^−*/*−^ and *Cib2*^−*/*−^*;Cib3*^−*/*−^ mice in open-field tests (*n* = 20 mice per group). Data are shown as means ± SEM. ***P < 0.001; ns no significant difference. Data were statistically analyzed using one-way ANOVA with Dunnett’s post hoc test.

Although the ion-channel-centered MET system may play a central role in equilibrioception, it may not fully represent all molecular constitutes of the MET apparatus in hair cells. Importantly, GPCRs may regulate the activity of ion channels by direct physical interaction and conformational transition, thus bypassing the time-consuming second messenger system.^42–44^ We thus cannot exclude the possibility that, in addition to ion channels, GPCRs may also actively participate and play modulatory roles in equilibrioception (Fig. 1a). We speculate that these equilibrioception receptors should fulfill the following criteria: (1) they are expressed in the stereocilia or on the apical membrane of VHCs; (2) they can directly sense force in a physiological range (2–100 dynes/cm^2^); (3) they are able to convert force stimuli into chemical or electrical signals in VHCs or neurotransmitter release from VHCs; and (4) genetic ablation of these receptors in animal models leads to balance disorder.

According to the above criteria for equilibrioception receptors, in the present study, we screened the mechanosensitivity of aGPCRs in utricular hair cells. Specifically, we identify LPHN2 as a mechanosensitive receptor, which is expressed at the apical surface of VHCs. Lphn2 deficiency impairs balance behavior in mice. Moreover, the MET current in VHCs is impaired either by genetic ablation of *Lphn2* or by pharmacological inhibition of LPHN2 with a specific inhibitor in a reversible manner. Furthermore, force sensation by LPHN2 in utricular hair cells induces Ca^2+^ response and glutamate release. Collectively, our findings suggest that a mechanosensitive GPCR is required for normal balance; this receptor actively regulates a previously uncharacterized MET process at the apical surface of VHCs.

## Results

### Screening of mechanosensitive aGPCRs in the vestibular system

The human aGPCR family consists of 33 members, while mice have 30. Several of these receptors are known to be activated by mechanical forces (Supplementary information, Fig. S1a).^15,16,21,24,45–47^ Single-cell RNA sequencing (scRNA-seq) data (GSE71982) indicate that 12 aGPCRs are expressed in more than 20% of the utricular hair cells of the mice (Fig. 1b).^48^ We then established a high-throughput mechanical stimulation assay to examine the mechanical sensitivity of the 12 aGPCRs using a magnetic tweezer system integrated with a GPCR biosensor platform. In this assay, tensile forces were applied to paramagnetic beads coated with either anti-Flag M2 antibody or polylysine using a magnetic system, and force-induced G protein activation was analyzed using a bioluminescence resonance energy transfer (BRET) assay in HEK293 cells transfected with plasmids encoding N-terminal Flag-tagged GPCR and G protein biosensors (Fig. 1c).^49,50^ In response to magnetic stimulation, tension forces were produced by magnetic beads attached to the N-terminus of selected aGPCRs. The resulting activation of Gs or Gi signaling, downstream of mechanosensitive GPCRs, was measured using a BRET assay — a well-established method for detecting GPCR activation.^16,20,50–54^ Specifically, a force of 10 pN was applied via magnetic beads, corresponding to 100 dynes/cm^2^ on the plasma membrane. This force was previously used to mimic arterial wall shear stress, which is ~1 m/s under physiological conditions and similar to the normal walking pace.^55^ Using this system, we revealed that five receptors (GPR133, GPR126, LPHN2, LPHN3 and VLGR1) activated Gi signaling in response to force stimulation; and among these, three receptors (GPR133, LPHN2 and VLGR1) also activated Gs signaling when subjected to force (Fig. 1d). To verify the mechanosensitivity of these GPCRs, we also employed a fluid-jet system and examined whether the mechanical stimuli impinging on cell membrane could activate downstream signaling of these receptors in HEK293 cells using G protein dissociation BRET assay (Supplementary information, Fig. S1b). GPR68, a well-established mechanosensitive GPCR that activates Gq-Ca^2+^ signaling, was employed as a positive control.^56^ We found that a one-time step fluid-jet specifically initiated Gi activation downstream of LPHN3, and the same mechanical stimulus simultaneously evoked both Gs and Gi activation in HEK293 cells expressing LPHN2 (Supplementary information, Fig. S1c–e). As a negative control, no activation of any G protein subtype was detected in HEK293 cells transfected with G protein BRET probes and the empty vector pcDNA3.1 (Supplementary information, Fig. S1c–e). Therefore, these findings align with results obtained from the magnetic beads assay and further support the mechanosensitivity of the tested aGPCRs.

We then investigated whether these mechanosensitive aGPCRs are required for equilibrioception by comparing *Gpr133*^−/−^ mice, *Lphn2*^+/−^ mice, *Lphn3*^+/−^ mice, *Atoh1*-*Cre*^+/−^;*Gpr126*^*fl/fl*^ mice, *Vlgr1*^−/−^ (*Vlgr1*/del7TM) mice with wild-type (WT) littermates in the open field test, which is a commonly used assay for evaluating vestibular functions (Fig. 1e–g). The *Cib2*^−*/*−^*;Cib3*^−*/*−^ double knockout mice, which had significant balance defects, were used as a positive control.^57^ *Lphn2*^+/−^ mice and *Lphn3*^+/−^ mice were used since homozygous ablation of *Lphn2* or *Lphn3* caused embryonic lethality and developmental defects, respectively.^58,59^ *Gpr126* deficiency also leads to embryonic lethality due to cardiac abnormalities^60^; therefore, we generated *Atoh1*-*Cre*^+/−^;*Gpr126*^*fl/fl*^ mice to eliminate Gpr126 expression in hair cells (*Atoh1* is a marker gene for nascent cochlear and VHCs,^61,62^ and scRNA-seq data suggested that ~85% of *Gpr126*-expressing utricle hair cells have detectable *Atoh1*). The deficiency of the target receptor genes in the mutant mice was verified by genotyping and western blotting analysis (Supplementary information, Figs. S1f–m, S2a–j). All of the above mice were viable, fertile (except for the *Gpr133*^−*/*−^ female mice, which were sterile) and maintained normal body weights when fed a normal chow diet (Supplementary information, Fig. S2k, l). Notably, with *Cib2*^−/−^;*Cib3*^−/−^ mice as a positive control, behavioral analyses of these mice in the open field test revealed that the *Gpr133*^−*/*−^ mice and *Lphn2*^+/−^ mice exhibited ~1.5–2-fold increases in circling behavior and traveling distances compared with their WT littermates in both the 2 min timeframe (*Gpr133*^−*/*−^: 12.0 ± 0.6 circles and 21.4 ± 1.3 meters; *Lphn2*^+/−^: 11.6 ± 0.6 circles and 16.0 ± 1.0 meters; WT: 6.0 ± 0.4 circles and 7.0 ± 0.6 meters) and the 10 min timeframe (*Gpr133*^−*/*−^: 42.9 ± 1.2 circles and 80.8 ± 1.6 meters; *Lphn2*^+/−^: 39.3 ± 0.9 circles and 54.9 ± 2.4 meters; WT: 23.8 ± 1.1 circles and 36.0 ± 1.5 meters). In contrast, the *Atoh1*-*Cre*^+/−^;*Gpr126*^*fl/fl*^ mice, *Vlgr1*^−/−^ mice, and *Lphn3*^+/−^ mice did not show significantly abnormal circling or traveling behaviors compared with the WT controls (Fig. 1e–g). These results suggest that mechanosensitive LPHN2 and GPR133 may play a role in maintenance of normal balance.

### The mechanosensitive LPHN2 is required for normal vestibular functions

Because a functional and mechanistic analysis of GPR133 was described in another study,^63^ we focused on LPHN2 in the current manuscript. We assessed LPHN2 expression in the vestibular system by quantitative reverse transcription polymerase chain reaction (RT-qPCR) analysis and revealed that LPHN2 maintained constant and stable expression from late embryonic stages (embryonic day 15, E15) to adulthood (postnatal day 120, P120) (Supplementary information, Fig. S3a). Both the scRNA-seq data (GSE71982, GSE155966 and GSE207817) and the results from our single-hair-cell RT-qPCR analysis confirmed the expression of LPHN2 in the utricular hair cells.^48,64,65^ Compared with LPHN3, another member of the mechanosensitive LPHN family expressed in utricular hair cells, LPHN2 exhibited a significantly higher frequency of expression and a greater average expression level (Supplementary information, Fig. S3b–g).

The vestibular behaviors of the *Lphn2*^+/−^ mice at P40 were then assessed using rotarod test and forced swimming test, with the *Cib2*^−/−^;*Cib3*^−/−^ mice as a positive control. Despite their nearly normal performance in the rotarod test (*Lphn2*^+/−^ 109.8 ± 3.7 s vs WT 110.6 ± 4.0 s), the *Lphn2*^+/−^ mice exhibited impaired swimming ability, with a swimming score of 0.88 ± 0.13 (the 0–3 scoring system was used; WT mice and *Cib2*^−/−^;*Cib3*^−/−^ mice scored 0 and 2.71 ± 0.13, respectively) (Supplementary information, Fig. S4a, b). To specifically investigate the vestibular function of *Lphn2*^+/−^ mice, we measured their vestibular-ocular reflex (VOR) during sinusoidal head rotations. Notably, compared with WT mice, the *Lphn2*^+/−^ mice showed an ~25%–50% decrease in compensatory VOR gains in response to both earth-vertical and off-vertical axis rotations, suggesting deficits in the semicircular canals and otolith system (Supplementary information, Fig. S4c–f). In contrast to *Lphn2*^+/−^ mice, *Lphn3*^+/−^ mice did not significantly differ from WT mice in the rotarod test, forced swimming test or VOR test; thus, *Lphn3*^+/−^ mice served as a negative control for vestibular behavior analyses (Supplementary information, Fig. S4a–f). Collectively, these results indicated that LPHN2 plays an important role in balance maintenance.

### Expression pattern of LPHN2 in VHCs

**Fig. 2:**
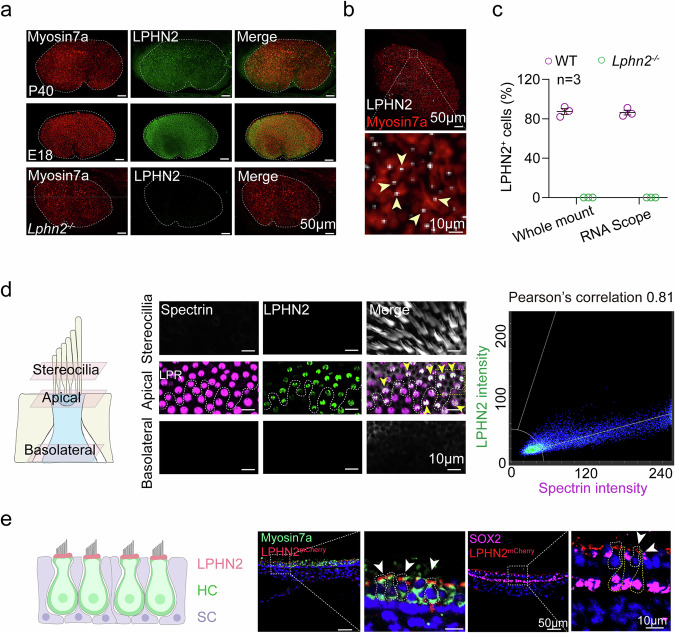
LPHN2 is primarily expressed at the apical surface of utricular hair cells. **a** Co-immunostaining of LPHN2 (green) with Myosin7a (red) in utricle wholemounts derived from WT mice at E18 or P40, or from *Lphn2*^−*/*−^ mouse embryos at E18 (*n* = 3 mice per group). Scale bars, 50 μm. **b** Representative images of wholemount RNAscope in situ hybridization of *Lphn2* (white) combined with immunostaining of Myosin7a (red) in utricles of P40 mice (*n* = 3 mice). Arrows indicate *Lphn2* staining in myosin7a-expressing hair cells. Scale bars, 50 μm and 10 μm for low- and high-magnification views, respectively. **c** Quantitative analysis of LPHN2 expression in myosin7a-positive utricular hair cells from WT mice or *Lphn2*^−*/*−^ embryos. Data are correlated to Fig. 2a, b (*n* = 3 mice per group). **d** Left panel: Diagram of utricular hair cells showing the selected optical planes (stereocilia, apical surface or basolateral section) for imaging by confocal microscopy. Middle panel: Co-immunostaining of LPHN2 (green) with spectrin (magenta) or phalloidin (gray) at different optical planes of hair cells in utricle wholemounts of P40 mice. Arrows indicate co-immunostaining of LPHN2 with spectrin. The line of polarity reversal (LPR) is depicted as a white dotted line. Scale bars, 10 μm. Right panel: Pearson’s correlation analysis of the fluorescence intensities of LPHN2 and spectrin at the apical surface of utricular hair cells was performed, revealing a correlation coefficient of 0.81. Data are correlated to Supplementary information, Fig. S5l, m. **e** Left panel: Schematic view of LPHN2 (red) expression in utricular hair cells. HC hair cells, SC supporting cells. Right panel: Expression of LPHN2-mCherry (red) with Myosin7a (green) or with SOX2 (magenta) in utricular sections derived from *Lphn2*^*mCherry*^ mice at P40 (*n* = 3 mice per group). Arrows indicate the distribution of LPHN2-mCherry at the apical surface of utricular hair cells. The utricular hair cells and supporting cells are depicted by white and yellow dashed lines, respectively. Scale bars, 50 μm and 10 μm for low- and high-magnification views, respectively.

We next examined the expression patterns of LPHN2 in the mouse utricle by whole-mount immunostaining and found that LPHN2 was expressed in ~80% of the Myo7a-positive hair cells (Fig. 2a). The specificity of the LPHN2 antibody was demonstrated by both the western blotting results in HEK293 cells expressing different LPHNs and the immunostaining of utricles derived from *Lphn2*^−/−^ embryos (*Lphn2*^−/−^ mice showed embryonic lethality but exhibited normal utricle morphology during embryonic period, with comparable macular size, hair cell density and stereocilia polarity to WT utricles of the same age) (Fig. 2a; Supplementary information, Fig. S5a–f). The expression pattern of *Lphn2* was further supported by RNAscope in situ hybridization, which revealed a comparable percentage of LPHN2-expressing hair cells (~80%) to that determined by immunostaining (Fig. 2b, c). Co-immunostaining of LPHN2 with different hair cell markers, including the type I hair cell marker oncomodulin (OCM) in the striolar (S) region, the type I hair cell marker osteopontin (OPN) in the extrastriolar (ES) region, and the type II hair cell marker Annexin A4 (Anxa4), revealed that LPHN2 was distributed in all three types of hair cells. While LPHN2 showed nearly equal distribution in type I and II hair cells in the S region (51% vs 49%), it presented a relatively higher expression frequency in type II hair cells than in type I cells in the ES region (58% vs 42%) (Supplementary information, Fig. S5g–k).

To further determine the subcellular localization of LPHN2 in utricular hair cells, we examined the expression pattern of LPHN2 in different optical sections spanning from the stereocilia to the hair cell body. LPHN2 expression was found primarily at the apical surface of the utricular hair cells, as revealed by its close proximity to spectrin, which is a marker of the cuticular plate in hair cells^66,67^ (Fig. 2d; Supplementary information, Fig. S5l). In contrast, LPHN2 expression was not found within the stereocilia (Fig. 2d; Supplementary information, Fig. S5l, m). The primary expression of LPHN2 at the apical surface was further supported by section staining of the utricle sensory epithelium using Myo7a and Sox2 as hair cell and supporting cell markers, respectively (Supplementary information, Fig. S5n). Consistent with the antibody-based expression analysis, we observed LPHN2-mCherry expression at the apical surface of the utricular hair cells, utilizing a *Lphn2*^*mCherry*^ transgenic knock-in mouse line (Fig. 2e). While ~86% of utricular hair cells showed LPHN2 staining exclusively at the apical surface, 14% displayed LPHN2 staining at the bottom or at both the apical surface and bottom (Supplementary information, Fig. S5o). Analysis of LPHN2 fluorescence intensity revealed a similar result, with ~90% staining at the apical surface vs ~10% staining at the bottom (Supplementary information, Fig. S5p).

The significantly decreased gain values of *Lphn2*^+/−^ mice in vertical VOR responses to off-vertical rotation also suggest a potential regulatory role of LPHN2 in the saccule (Supplementary information, Fig. S4c–f). We accordingly examined the expression of LPHN2 in mouse saccule by whole-mount immunostaining and found that LPHN2 was expressed in ~47% of the saccular hair cells (Supplementary information, Fig. S5q, r). Similar to its subcellular expression pattern in the utricle, LPHN2 was observed exclusively at the apical surface of saccular hair cells but not in the stereocilia, as revealed by optical sectioning microscopy (Supplementary information, Fig. S5s). The expression pattern of the mechanosensitive LPHN2 at the apical membrane of VHCs suggests that it may participate in force sensation during equilibrioception.

### LPHN2 in VHCs specifically regulates balance sensation

**Fig. 3:**
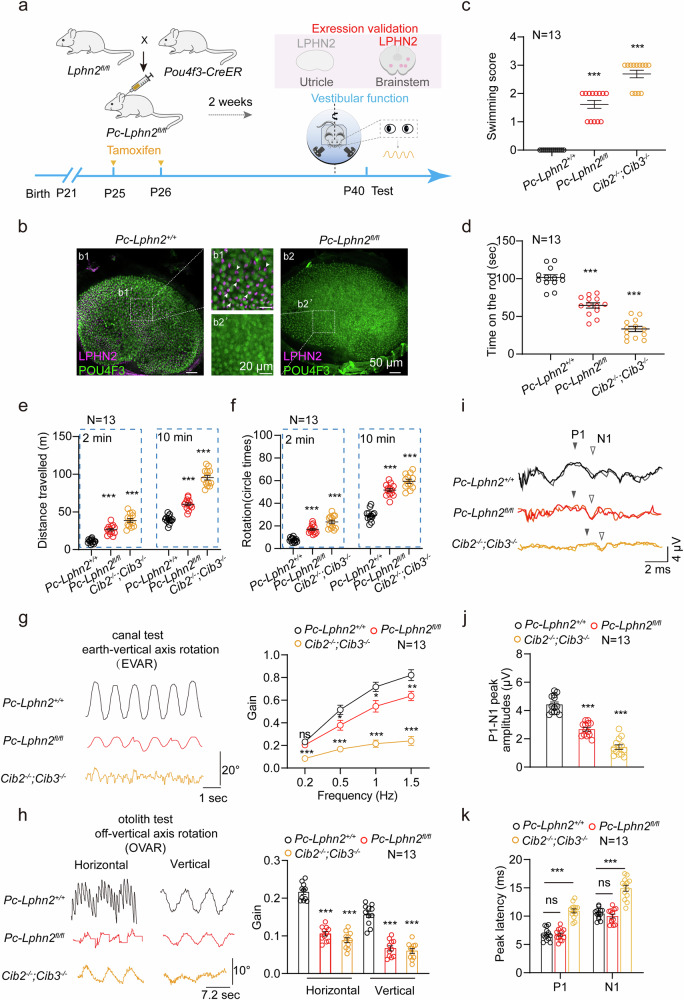
LPHN2-deficiency in mouse hair cells impairs balance. **a** Schematic representation of the crossbreeding strategy to generate hair-cell-specific *Lphn2*-knockout mice and the time scales for vestibular functional analysis. The *Pou4f3-CreER*^*+/*−^;*Lphn2*^*fl/fl*^ mice (referred to as *Pc-Lphn2*^*fl/fl*^) or *Pou4f3-CreER*^*+/*−^;*Lphn2*^*+/+*^ mice (referred to as *Pc-Lphn2*^*+/+*^) were treated with tamoxifen (75 mg/kg) dissolved in corn oil through round window membrane injection at P25 (left ear) and P26 (right ear) consecutively, and vestibular behavior tests were performed at P40. **b** Immunostaining of LPHN2 (magenta) and POU4F3 (green) in utricle wholemounts derived from *Pc-Lphn2*^*fl/fl*^ or *Pc-Lphn2*^*+/+*^ mice (*n* = 3 mice per group). Enlarged images show the ablation of LPHN2 in the utricular hair cells of *Pc-Lphn2*^*fl/fl*^ mice. Scale bars, 50 μm and 20 μm for low and high magnification views, respectively. **c**–**f** Quantification of the swimming scores (**c**), time on the rotating rod (**d**), traveling activity (**e**) and circling activity (**f**) in the open field test of *Pc-Lphn2*^*fl/fl*^ mice, *Pc-Lphn2*^*+/+*^ mice and *Cib2*^−*/*−^*;Cib3*^−*/*−^ mice (*n* = 13 mice per group). Data are shown as means ± SEM. ***P < 0.001. Data were statistically analyzed using one-way ANOVA with Dunnett’s post hoc test. **g** Representative recording curves (left panel) and quantification of the VOR gain values (right panel) of *Pc-Lphn2*^*fl/fl*^ mice, *Pc-Lphn2*^*+/+*^ mice and *Cib2*^−*/*−^*;Cib3*^−*/*−^ mice in response to earth-vertical axis rotation (*n* = 13 mice per group). Data are shown as means ± SEM. *P < 0.05; **P < 0.01; ***P < 0.001; ns, no significant difference. Data were statistically analyzed using two-way ANOVA with Dunnett’s post hoc test. **h** Representative recording curves (left panel) and quantification of the VOR gain values (right panel) of *Pc-Lphn2*^*fl/fl*^ mice, *Pc-Lphn2*^*+/+*^ mice and *Cib2*^−*/*−^*;Cib3*^−*/*−^ mice in response to off-vertical axis rotation (*n* = 13 mice per group). Data are shown as mea*n*s ± SEM. ***P < 0.001. Data were statistically analyzed using one-way ANOVA with Dunnett’s post hoc test. **i**–**k** Representative click-evoked VEMP waveforms (**i**), quantification of the P1–N1 peak amplitudes (**j**) and the P1 (filled triangle) and N1 (hollow triangle) peak latencies (**k**) of *Pc-Lphn2*^*fl/fl*^ mice, *Pc-Lphn2*^*+/+*^ mice and *Cib2*^−*/*−^*;Cib3*^−*/*−^ mice at 100 dB nHL (*n* = 13 mice per group). Data are shown as means ± SEM. ***P < 0.001; ns, no significant difference. Data were statistically analyzed using one-way with Dunnett’s post hoc test.

To determine the specific functional role of LPHN2 in VHCs, we crossed *Lphn2*^*fl/fl*^ mice with inducible *Pou4f3-CreER*^+/−^ transgenic mice (Fig. 3a). *Pou4f3* is the transcriptional target of ATOH1 and a commonly used marker of hair cells and is expressed in all detected LPHN2-positive utricular hair cells, as revealed by scRNA-seq data^48,68^ (Supplementary information, Fig. S6a, b). The specificity of LPHN2 ablation in the vestibule of the *Pou4f3-CreER*^+/−^;*Lphn2*^*fl/fl*^ mice was indicated by the loss of LPHN2 immunostaining in the Pou4f3-expressing utricular hair cells but not in other tissues, such as the vestibular or somatosensory nuclei in the brainstem (Fig. 3b; Supplementary information, Fig. S6c, d). The specific decrease in Lphn2 expression in the vestibules of the *Pou4f3-CreER*^+/−^;*Lphn2*^*fl/fl*^ mice was further demonstrated by western blotting analysis (Supplementary information, Fig. S6e).

Various behavioral tests were conducted to assess the effects of LPHN2 ablation in VHCs on balance maintenance, using the *Cib2*^−/−^;*Cib3*^−/−^ mice as a positive control. Notably, in addition to their significantly decreased swimming performance, the *Pou4f3-CreER*^+/−^;*Lphn2*^*fl/fl*^ mice spent ~50% less time on the rotarod than their *Pou4f3-CreER*^+/−^;*Lphn2*^*+/+*^ littermates, which was not observed in the *Lphn2*^+/−^ mice (Fig. 3c, d). Moreover, compared with their *Pou4f3-CreER*^+/−^;*Lphn2*^*+/+*^ littermates, the *Pou4f3-CreER*^+/−^;*Lphn2*^*fl/fl*^ mice exhibited an ~2-fold increase in circling times in both 2-min and 10-min testing timeframes, accompanied by significantly increased traveling distances (Fig. 3e, f; Supplementary information, Fig. S6f). Furthermore, compared with control mice, *Pou4f3-CreER*^+/−^;*Lphn2*^*fl/fl*^ mice showed a ~25%–50% decrease in VOR responses in both earth-vertical and off-vertical axis rotation tests (Fig. 3g, h). To further evaluate the effects of LPHN2 deficiency on vestibular function, we assessed vestibular-evoked myogenic potentials (VEMPs) in the *Pou4f3-CreER*^+/−^;*Lphn2*^*fl/fl*^ and *Pou4f3-CreER*^+/−^;*Lphn2*^*+/+*^ mice. While the positive peak (P1) and negative peak (N1) latency in the *Pou4f3-CreER*^+/−^;*Lphn2*^*fl/fl*^ mice was not significantly altered, the P1-N1 amplitude was reduced by ~50% compared with that of their control littermates (Fig. 3i–k). Collectively, these results suggest that LPHN2 in VHCs plays an important role in regulating the equilibration.

**Fig. 4:**
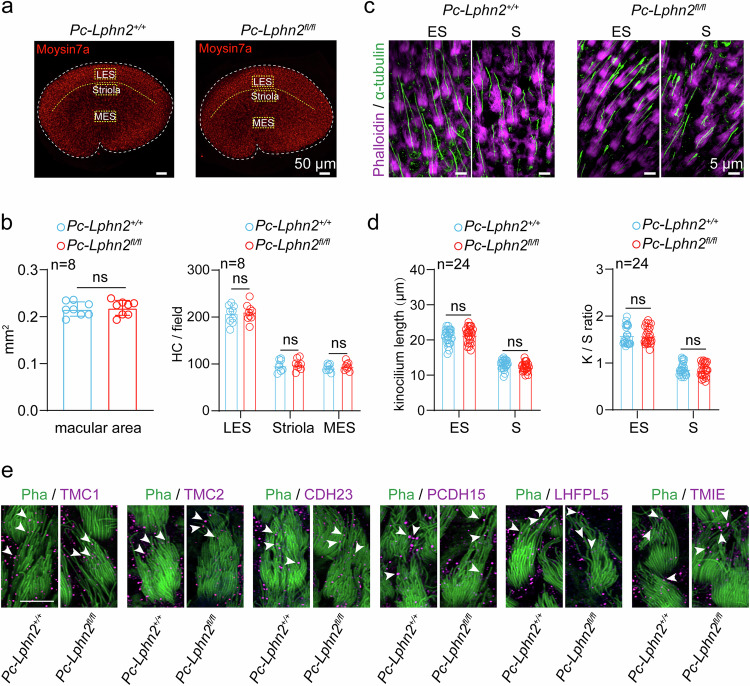
LPHN2-deficiency does not affect the morphology of the mouse utricle. **a** Immunostaining of Myosin7a (red) in utricular hair cells derived from *Pc-Lphn2*^*+/+*^ and *Pc-Lphn2*^*fl/fl*^ mice at P40 (*n* = 8 mice per group). Scale bars, 50 μm. Three fields of 100 μm × 50 μm were defined and outlined in the lateral extrastriolar (LES) region, striolar region (S) and medial extrastriolar (MES) region. **b** Quantification of the size of utricles (left panel) and hair cell density at different regions of utricles (right panel) derived from *Pc-Lphn2*^*+/+*^ and *Pc-Lphn2*^*fl/fl*^ mice (*n* = 8 mice per group). Data are correlated to Fig. 4a. Data are shown as means ± SEM. ns no significant difference. Data were statistically analyzed using unpaired two-sided Student’s *t*-test. **c** Immunostaining of kinocilium (labeled with α-tubulin, green) and stereocilia (labeled with phalloidin, magenta) in utricle wholemounts derived from *Pc-Lphn2*^*+/+*^ and *Pc-Lphn2*^*fl/fl*^ mice (*n* = 3 mice per group). Scale bars, 5 μm. **d** Quantification of the length of kinocilium (left panel) and the ratio of lengths of the kinocilium to tallest stereocilia (right panel) in ES or S region of utricle wholemounts derived from *Pc-Lphn2*^*+/+*^ and *Pc-Lphn2*^*fl/fl*^ mice (*n* = 24 hair cells from 3 mice per group). Data are correlated to Fig. 4c. Data are shown as means ± SEM. ns no significant difference. Data were statistically analyzed using unpaired two-sided Student’s *t*-test. **e** Co-immunostaining of phalloidin (green) and different MET machinery components (magenta), including TMC1, TMC2, CDH23, PCDH15, LHFPL5 and TMIE, in utricular hair cells derived from *Pc-Lphn2*^*+/+*^ and *Pc-Lphn2*^*fl/fl*^ mice (*n* = 6 mice per group). Scale bar, 5 μm. Data are correlated to Supplementary information, Fig. S6g, h.

To determine whether the impaired balance in the LPHN2-deficient mice was due to defects in vestibular organ development, we further examined the morphology of utricles and utricular hair cells derived from *Pou4f3-CreER*^+/−^;*Lphn2*^*fl/fl*^ mice at P40. Our results indicated that the overall size of the utricular macula and the number of hair cells in different areas (S region, lateral ES region and medial ES region) of the utricle of *Pou4f3-CreER*^+/−^;*Lphn2*^*fl/fl*^ mice were comparable to those of their *Pou4f3-CreER*^+/−^;*Lphn2*^*+/+*^ littermates (Fig. 4a, b). At the subcellular level, the apical surface (cuticular plate) size of the utricular hair cells, the kinocilium length and the stereocilia structure of *Pou4f3-CreER*^+/−^;*Lphn2*^*fl/fl*^ utricle were not significantly affected by *Lphn2* ablation compared with those of their *Pou4f3-CreER*^+/−^;*Lphn2*^*+/+*^ littermates (Fig. 4c, d; Supplementary information, Fig. S5l, m). Moreover, the expression levels and localization of MET channel components, including TMC1, TMC2, TMIE, LHFPL5 and PCDH15, in utricular hair cells of *Pou4f3-CreER*^+/−^;*Lphn2*^*fl/fl*^ mice were comparable to those in *Pou4f3-CreER*^+/−^;*Lphn2*^*fl/fl*^ mice, as revealed by the quantification of immunostaining signals for the respective proteins in the stereocilia (Fig. 4e; Supplementary information, Fig. S6g–k). These data collectively support the normal morphology of the *Pou4f3-CreER*^+/−^;*Lphn2*^*fl/fl*^ utricles and suggest that LPHN2 plays a regulatory role in balance sensation.

### Genetic disruption or pharmacological blockade of LPHN2 impairs the MET current in VHCs

**Fig. 5:**
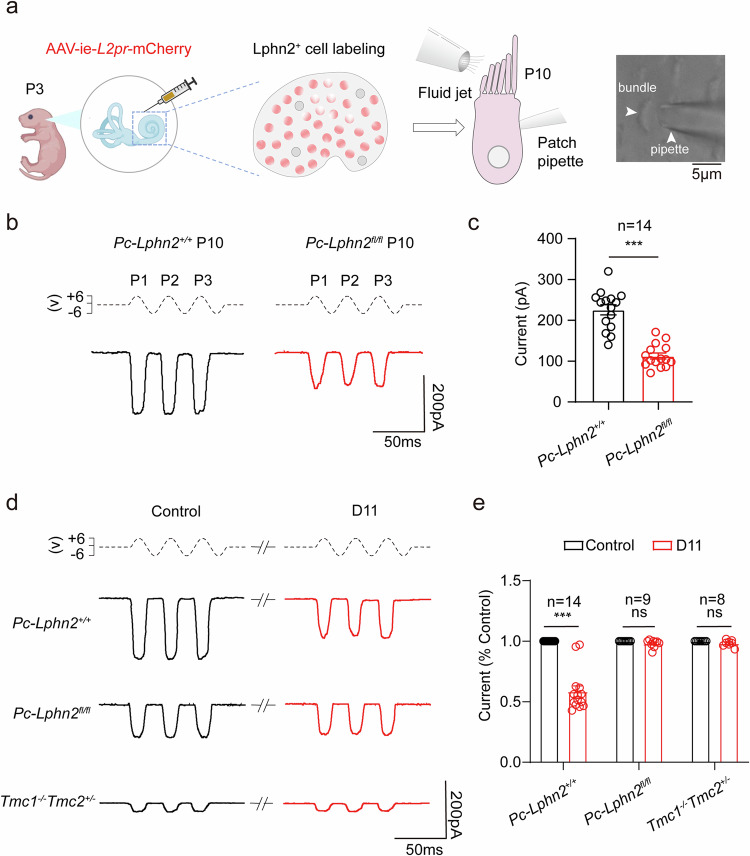
LPHN2-deficiency impairs MET currents in utricular hair cells. **a** Schematic illustration of the labeling of LPHN2-expressing utricular hair cells by AAV-ie-*Lphn2pr*-mCherry (referred to as AAV-ie-*L2pr*-mCherry) and the MET current recording by fluid-jet stimulation. The Cre recombinase was activated in *Pou4f3-CreER*^*+/*−^ mouse embryos by treating the pregnant mice at E14 with 100 mg/kg tamoxifen supplemented with 37.5 mg/kg progesterone for 3 consecutive days through intraperitoneal injection. AAV-ie-*Lphn2pr*-mCherry was injected into P3 mice through round window membrane, and the mCherry-labeled utricular hair cells at P10 were selected for MET current recording. **b** Representative MET current traces induced by sinusoidal fluid jet stimulation in utricular hair cells of *Pc-Lphn2*^*+/+*^ mice (black) or *Pc-Lphn2*^*fl/fl*^ mice (red) at P10. **c** Quantification of the peak MET currents in utricular hair cells of *Pc-Lphn2*^*+/+*^ mice or *Pc-Lphn2*^*fl/fl*^ mice at P10 (*n* = 14). Data are correlated to Fig. 5b. Data are shown as means ± SEM. ***P < 0.001. Data were statistically analyzed using unpaired two-sided Student’s *t*-test. **d**, **e** Representative current traces (**d**) and quantitative analysis (**e**) of fluid-jet-stimulated MET responses in utricular hair cells derived from *Pc-Lphn2*^*+/+*^, *Pc-Lphn2*^*fl/fl*^ or *Tmc1*^−*/*−^*;Tmc2*^+/−^mice in the absence (black) or presence (red) of 50 nM D11. Data are normalized to the peak MET current of control vehicle-treated hair cells in respective groups (*n* = 14, 9 a*n*d 8 for *Pc-Lphn2*^*+/+*^, *Pc-Lphn2*^*fl/fl*^ and *Tmc1*^−*/*−^*;Tmc2*^+/−^, respectively). Data are correlated to Supplementary information, Fig. S7e. Data are shown as means ± SEM. ***P < 0.001; ns, no significant difference. Data were statistically analyzed using paired two-sided Student’s *t***-**test.

To investigate whether LPHN2 participates in equilibrioception, we examined MET responses using isolated utricles and saccules. We employed a fluid jet system to deflect hair bundles in VHCs and recorded the corresponding MET currents using a whole-cell patch-clamp technique.^7,69,70^ To record the MET response in the LPHN2-expressing VHCs, we labeled these cells by using a modified AAV-ie-*Lphn2pr*-mCherry vector, which enabled the expression of the fluorescent protein mCherry driven by the *Lphn2* promoter (Fig. 5a; Supplementary information, Fig. S7a, b). AAV-ie-*Lphn2pr*-mCherry was injected into P3 mice through round window membrane, and the mCherry-labeled VHCs at P10 were selected for fluid jet stimulation and MET recording at a holding potential of –64 mV.^71^ Notably, the peak MET currents in the mCherry-labeled utricular hair cells and saccular hair cells of *Pou4f3-CreER*^+/−^;*Lphn2*^*fl/fl*^ mice were reduced by ~50% and ~40%, respectively, compared with those in control littermates (utricle: 225.8 ± 12.4 pA vs 112.4 ± 7.4 pA; saccule: 190.4 ± 31 pA vs 117.8 ± 27 pA), suggesting that LPHN2 is involved in the MET process in VHCs (Fig. 5b, c; Supplementary information, Fig. S7c, d). However, LPHN2 deficiency in utricular hair cells did not appear to affect the hair bundle stiffness or the tip-link-mediated MET currents, as revealed by the comparable current displacement plots between control and *Pou4f3-CreER*^+/−^;*Lphn2*^*fl/fl*^ utricular hair cells when stimulating the hair bundle with a stiff glass probe (Supplementary information, Fig. S7e, f).

To further investigate the regulatory role of LPHN2 in MET and to exclude the possibility that dampened MET in LPHN2-deficient mice might be caused by potential deficits in certain MET machinery components, we utilized a reversible and selective inhibitor of LPHN2, named D11,^72^ to investigate the regulatory mechanism of LPHN2 in vestibular MET. Consistent with the MET data obtained from *Lphn2*-deficient VHCs, pretreatment of utricular or saccular explants from *Pou4f3-CreER*^+/−^;*Lphn2*^*+/+*^ mice with 50 nM D11 caused an ~40%–45% decrease in MET currents, which returned to normal levels after D11 was removed (Fig. 5d, e; Supplementary information, Fig. S7g–k). Specifically, D11 dose-dependently inhibited MET currents in utricular hair cells, with an EC_50_ value of 25 ± 5 nM, which was similar to that obtained for D11 in inhibiting mechanosensitivity of LPHN2 in HEK293 cells (20 ± 1 nM) (Supplementary information, Fig. S7l, m). As a negative control, the inhibitory effects of D11 on MET currents in *Lphn2* promoter-labeled VHCs were abolished in utricular hair cells derived from the *Pou4f3-CreER*^+/−^;*Lphn2*^*fl/fl*^ mice, suggesting a specific role for LPHN2 in MET regulation. Intriguingly, the residual MET response in VHCs derived from *Tmc1*^−*/*−^*;Tmc2*^+/−^ mice, which might be regulated by TMC2 compensation or by other unknown channels, was not significantly altered by D11 administration (Fig. 5d, e; Supplementary information, Fig. S7g). These data collectively suggest that the mechanosensitive LPHN2 participates in a tip-link-independent MET process in VHCs, potentially through crosstalk with TMC1, the ion-conducting pore of the MET channel complex.

### LPHN2 regulates the MET current at the apical surface of utricular hair cells

**Fig. 6:**
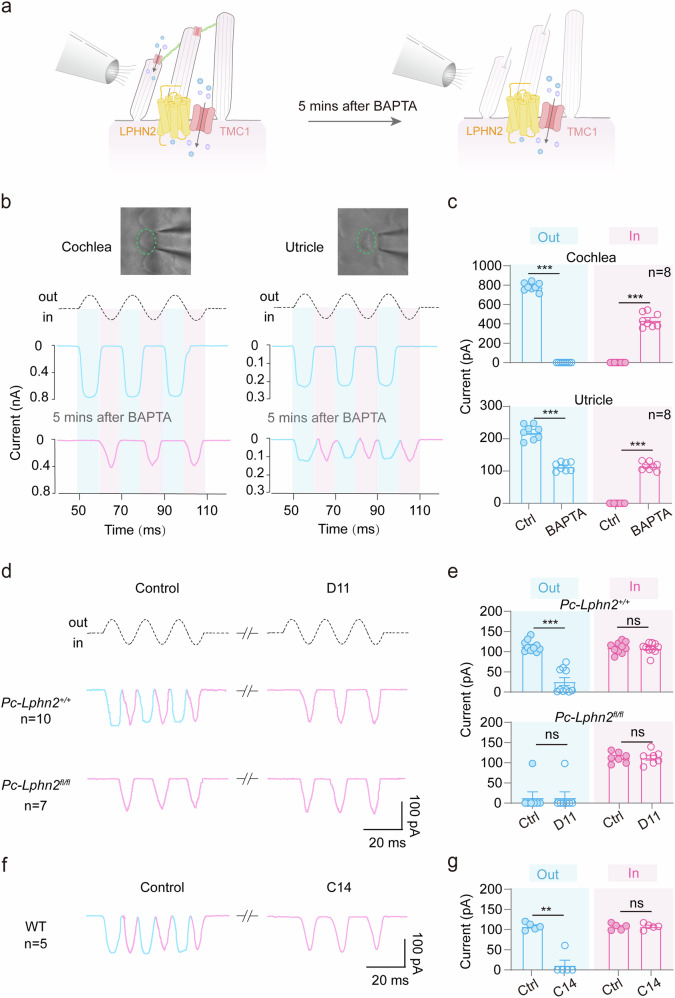
LPHN2 regulates the MET current at the apical surface of utricular hair cells. **a** Schematic illustration showing fluid-jet-stimulated MET responses in utricular hair cells before and after treatment with BAPTA, which disrupts the tip links. **b** Representative MET current traces induced by sinusoidal fluid jet stimulation in cochlear outer hair cells (OHCs) (left panel) or utricular hair cells (right panel) before and after treatment with BAPTA for 5 min (*n* = 8 per group). The normal-polarity and reverse-polarity MET current traces are colored blue and pink, respectively. The fluid-jet-stimulated cochlear or utricular hair cells are outlined in green. **c** Quantification of the normal-polarity (outward phase, blue) or reverse-polarity (inward phase, pink) MET current of cochlear (top) or utricular hair cells (bottom) in response to fluid jet stimulation (*n* = 8 per group). Data are shown as means ± SEM. ***P < 0.001. Data were statistically analyzed using paired two-sided Student’s *t*-test. **d**, **e** Representative current traces (**d**) and quantitative analysis (**e**) of fluid-jet-stimulated MET responses in BAPTA-treated utricular hair cells derived from *Pc-Lphn2*^*+/+*^ or *Pc-Lphn2*^*fl/fl*^ mice in the absence or presence of 50 nM D11 (*n* = 10 and 7 for *Pc-Lphn2*^*+/+*^ and *Pc-Lphn2*^*fl/fl*^ mice, respectively). Data are shown as means ± SEM. ***P < 0.001; ns no significant difference. Data were statistically analyzed using paired two-sided Student’s *t*-test. **f**, **g** Represen*t*ative current traces (**f**) and quantitative analysis (**g**) of fluid-jet-stimulated MET responses in BAPTA-treated WT utricular hair cells in the absence or presence of 1 μM C14 (*n* = 5). Data are show*n* as means ± SEM. **P < 0.01; ns no significant difference. Data were statistically analyzed using paired two-sided Student’s *t*-test.

Previous studies have indicated that in the fluid jet assay, the outward phase of the sinusoidal flow induces a normal-polarity MET current by deflecting the hair bundle toward the longest stereocilia in mature cochlear or VHCs; in contrast, the inward fluid flow, deflecting the bundle in the opposite direction, can evoke a reverse-polarity current in hair cells lacking tip links or key MET channel components (e.g., *Tmc1*^−*/*−^*;Tmc2*^−/−^ or *Tmie*^−/−^).^69,70,73^ Following previously established protocol, we treated the utricular hair cells with the calcium-chelating agent BAPTA to disrupt tip links and investigated the potential role of LPHN2 in tip-link-independent MET in utricles (Fig. 6a). Consistent with previous reports, after cochlear explants were treated with BAPTA for 5 min, the normal-polarity MET currents (782.5 ± 15.0 pA) were replaced by reverse-polarity currents (439.3 ± 26.0 pA) in response to sinusoidal fluid jet stimulation (Fig. 6b, c).^69,70^ Unexpectedly, treatment of utricular explants with BAPTA under the same conditions as cochlear explants resulted in unique biphasic MET currents, characterized by a reduced normal-polarity MET current (a 48.2% decrease compared to the control normal-polarity current) and the emergency of a reverse-polarity current (Fig. 6b, c). These data suggest the coexistence of tip-link-independent normal-polarity and reverse-polarity MET currents in utricular hair cells, which was different from the MET characteristics of cochlear hair cells (CHCs).

Notably, the residual normal-polarity MET current in the BAPTA-treated utricular hair cells recorded from WT or *Pou4f3-CreER*^+/−^;*Lphn2*^*+/+*^ mice was absent in those recorded from *Pou4f3-CreER*^+/−^;*Lphn2*^*fl/fl*^ mice (Fig. 6d, e). Therefore, we speculated that LPHN2 expressed at the apical surface of utricular hair cells might be responsible for tip-link-independent normal-polarity MET currents. Consistent with this hypothesis, pretreatment with the LPHN2-specific inhibitor D11 (50 nM) abrogated the residual normal-polarity MET current in the BAPTA-treated utricular hair cells derived from the *Pou4f3-CreER*^+/−^;*Lphn2*^*+/+*^ mice, which returned to normal levels after D11 was washed out, suggesting that the residual current was LPHN2-dependent (Fig. 6d, e; Supplementary information, Fig. S8a, b). In contrast, the reverse-polarity currents were not significantly affected by D11 treatment in either *Pou4f3-CreER*^+/−^;*Lphn2*^*+/+*^ or *Pou4f3-CreER*^+/−^;*Lphn2*^*fl/fl*^ utricular hair cells (Fig. 6d, e).

To further test the possibility that the residual normal-polarity MET currents were mediated by any remaining tip links, we recorded the MET currents in utricular hair cells before and after BAPTA treatment by stimulating the hair bundle with a stiff glass probe. We showed that after 5-mM BAPTA treatment for 5 min, the MET currents in these hair cells induced by hair bundle deflection were completely abolished, indicating the disruption of all the functional tip links (Supplementary information, Fig. S8c, d). We also examined the Ca^2+^ signals in BAPTA-treated utricular hair cells in response to force stimulation with magnetic beads that were coated with anti-CDH23 antibody (recognizing the residues 141–450 at the N-terminus; referred to as CDH23-M-beads), which could theoretically pull tip links and induce Ca^2+^ response through normal-polarity MET. Consistently, we observed similar CDH23-M-beads-stimulated Ca^2+^ responses in mouse utricular hair cells derived from *Pou4f3-CreER*^+/−^*Lphn2*^*+/+*^ and *Pou4f3-CreER*^+/−^*Lphn2*^*fl/fl*^ mice (Supplementary information, Fig. S8e, f). However, we did not observe any CDH23-M-beads-elicited Ca^2+^ response in BAPTA-treated WT utricular hair cells (Supplementary information, Fig. S8g, h). These results collectively support that the residual normal-polarity MET currents in BAPTA-treated utricular hair cells are LPHN2-dependent and not due to the persistence of tip links.

To further determine the potential roles of TMC1 in tip-link-independent MET currents, we developed a reversible inhibitor through structure-based in silico screening (a simulated structure of mouse TMC1 was modeled using Alphafold2) (Supplementary information, Fig. S8i–l), named C14. This inhibitor C14 showed inhibitory effects on TMC1, but not on several other ion channels, such as the CNG channel in vitro or sodium channels in primary utricular hair cells (Supplementary information, Fig. S8m–o). We revealed that, similar to LPHN2, pharmacological inhibition of TMC1 by the inhibitor C14 also abolished the residual normal-polarity currents without affecting the reverse-polarity currents (Fig. 6f, g; Supplementary information, Fig. S8p). Collectively, these data indicate that LPHN2 plays an important role in regulating previously uncharacterized normal-polarity MET currents at the apical surface of utricular hair cells.

### Colocalization and functional coupling of LPHN2 with TMC1 at the apical surface

**Fig. 7:**
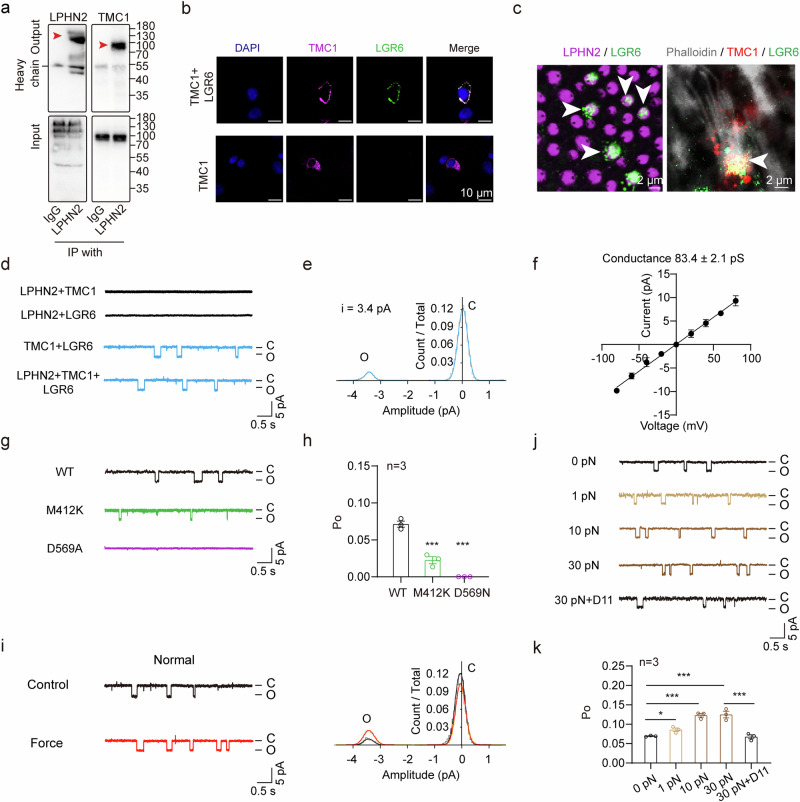
LPHN2 colocalizes with TMC1 at the apical surface of utricular hair cells and regulates MET through coupling to TMC1. **a** Co-immunoprecipitation of LPHN2 with TMC1 in the lysates of mouse utricles. Representative blots from three independent experiments are shown (*n* = 3). **b** Co-immunostaining of TMC1 (magenta) with LGR6 (green) in HEK293 cells transfected with TMC1 only or with TMC1 and LGR6. Scale bars, 10 μm. Representative images from three independent experiments are shown (n = 3). **c** Co-immunostaining of LGR6 (green) with LPHN2 (magenta) or with TMC1 (red) at the apical surface of utricular hair cells. Scale bars, 2 μm. Representative images from three independent experiments are shown (*n* = 3). **d** Representative spontaneous single-channel currents of TMC1 at –40 mV recorded in HEK293 cells co-transfected with LPHN2/TMC1, LPHN2/LGR6, TMC1/LGR6 or LPHN2/TMC1/LGR6. Representative current traces from three independent experiments are shown (*n* = 3). **e** The normalized all-point amplitude histogram analysis of single-channel currents in HEK293 cells transfected with TMC1/LGR6. The distribution data were fitted by a sum of two Gaussians, and the peaks correspond to the closed (C) and open (O) states. The histogram is correlated with the current trace in Fig. 7d and represents a time window of 5 s. **f** The current-voltage (I-V) relationship of the spontaneous currents recorded in HEK293 cells transfected with TMC1/LGR6 (*n* = 3). **g**, **h** Representative traces (**g**) and quantitative analysis (**h**) of the spontaneous single-channel currents at –40 mV recorded in HEK293 cells transfected with LGR6 and TMC1 (WT or mutants) (*n* = 3). Data are shown as means ± SEM. ***P < 0.001. Data were statistically analyzed using one-way ANOVA with Dunnett’s post hoc test. **i** Representative current traces (left panel) and histogram analysis (right panel) of the single-channel currents recorded in HEK293 cells transfected with LPHN2/TMC1/LGR6 under control condition (black) or in response to 10 pN force stimulation applied through LPHN2-M-beads (red). **j**, **k** Representative traces (**j**) and summarization of the channel open probability (**k**) of the single-channel currents recorded in HEK293 cells transfected with LPHN2/TMC1/LGR6 in response to varying force amplitudes (1 pN, 10 pN and 30 pN) applied through LPHN2-M-beads in the absence or presence of 50 nM D11 (*n* = 3). Data are shown as means ± SEM. *P < 0.05; ***P < 0.001. Data were statistically analyzed using paired two-sided Student’s *t*-test.

Our previous proteomic interactome analyses using purified LPHN2 as bait and co-immunostaining assay suggested a direct interaction between LPHN2 and TMC1 in the mouse cochlea.^72^ A similar interaction between LPHN2 and TMC1 was also detected by in vivo co-immunoprecipitation of mouse utricular lysates (Fig. 7a). In contrast, LPHN2 did not show direct interaction with PIZEO2, a mechanosensitive ion channel expressed in both cochlear and vestibular hair cells (Supplementary information, Fig. S9a). Several studies have shown that TMC1 is distributed at the apical surface of hair cells.^9,74,75^ We observed fluorescent puncta of TMC1 at the apical surface of the utricular hair cells by optical sectioning microscopy, where TMC1 was co-immunostained with LPHN2, suggesting the potential localized assembly of these two membrane proteins in vivo (Supplementary information, Fig. S9b).

To further study the interaction between LPHN2 and TMC1, we next attempted to reconstitute the functional coupling between LPHN2 and TMC1 in a heterologous system. We previously identified two GPCRs that are endogenously expressed in hair cells and can transport TMC1 to the plasma membrane in HEK293 cells^72^ (Fig. 7b). One of these two receptors, LGR6, was found to be expressed at the apical surface of utricle hair cells, where it was co-immunostained with LPHN2 and TMC1 (Fig. 7c). Therefore, we selected this receptor to function as the trafficking chaperone for TMC1 in the heterologous system, as we reasoned this would more closely mimic the endogenous landscape. At least 20% of HEK293 cells co-transfected with TMC1/LGR6 exhibited significant cell surface expression of TMC1 and these cells were selected for further analysis (Supplementary information, Fig. S9c, d). By performing patch-clamp recording at a holding potential of –40 mV, we observed comparable spontaneous single-channel opening in the HEK293 cells co-transfected with TMC1/LPHN2/LGR6 and the cells co-transfected with TMC1/LGR6, but not in the negative control cells co-transfected with TMC1/LPHN2 or with LPHN2/LGR6 (Fig. 7d). The average single-channel TMC1 current and conductance were 3.4 pA and 83.4 ± 2.1 pS, respectively, suggesting the trafficking of TMC1 onto the plasma membrane by LGR6 (Fig. 7e, f). Intriguingly, LGR6 can also chaperon the deafness-related TMC1 mutants, including M412K (*Beethoven*) and D569N, to the plasma membrane of HEK293 cells (Supplementary information, Fig. S9c, d). However, compared with the WT TMC1, M412K mutant showed an ~70% reduction in the single-channel open probability while D569N mutant nearly completely abolished the channel activity (Fig. 7g, h). These data are consistent with the results from a previous report studying the channel activity of TMC1 mutants in artificial liposomes and support the feasibility of this heterologous system for TMC1 characterization.^76^

We further revealed that application of force through LPHN2-M-beads to the heterologous system co-expressing LPHN2/TMC1/LGR6 induced a force-amplitude-dependent increase in the probability of TMC1 opening compared with the resting condition, which was abrogated by pretreatment with D11 (Fig. 7i–k). Notably, the LGR6 itself only acts as a trafficking chaperon of TMC1, but not a mechanical sensor that regulates TMC1 activity, since LGR6 could neither translate force stimulation into Gs/Gi activation nor affect the open probability of TMC1 in the heterologous system co-expressing LGR6 and TMC1 (Supplementary information, Fig. S9e–g).

To characterize TMC1 channel activity in response to a natural force stimulation, we recorded the fluid-jet-induced whole-cell currents in the heterologous system. An average current of ~75 pA was recorded in HEK293 cells co-expressing TMC1 and LGR6 in response to a one-time step fluid jet, which was abolished by pretreatment with TMC1 inhibitor C14 (Supplementary information, Fig. S9h, i). Incorporation of LPHN2 expression in the above system induced an ~1-fold increase in the maximal MET current (~130 pA), and this effect was abrogated by pretreatment with D11 (Supplementary information, Fig. S9j, k). As the negative controls, HEK293 cells transfected with TMC1 alone or co-transfected with TMC1/LPHN2 or LPHN2/LGR6 showed no detectable current in response to fluid jet stimulation (Supplementary information, Fig. S9h, i). In addition, LPHN2-mediated TMC1 opening upon force sensation was still observed in *PIEZO1-*knockout HEK293T cells, which showed no detectable endogenous *PIEZO2* expression, as revealed by RT-qPCR analysis (Supplementary information, Fig. S9l–p). Collectively, these results indicate that LPHN2 can sense extracellular mechanical stimuli to enhance the TMC1 opening.

### Force sensation by LPHN2 induces neurotransmitter release and Ca^2+^ influx in utricular hair cells

**Fig. 8:**
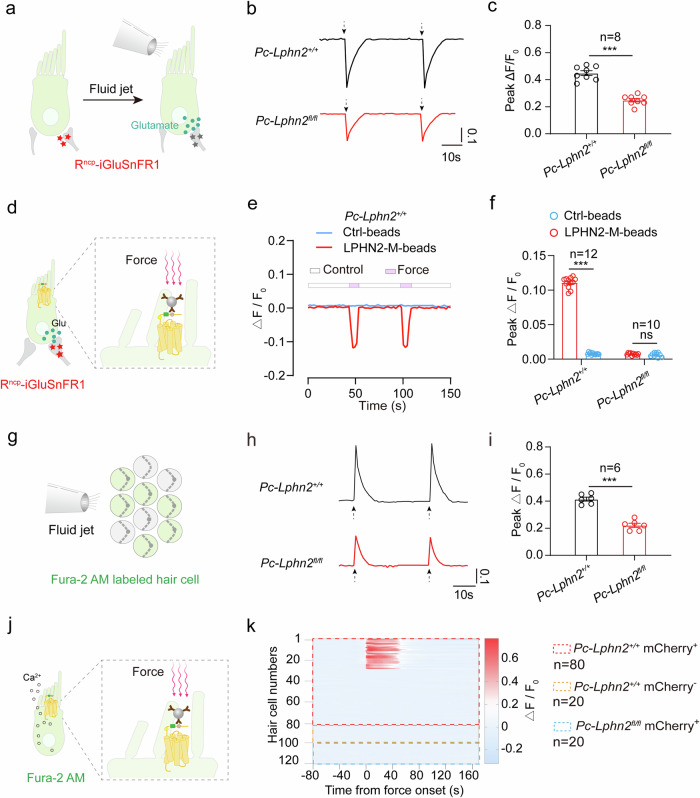
Force sensation by LPHN2 induces glutamate release and Ca^2+^ signals in utricular hair cells. **a** Schematic diagram showing the detection of fluid-jet-stimulated glutamate release in utricular hair cells by a glutamate reporter R^ncp^-iGluSnFR. **b**, **c** Representative traces (**b**) and quantitative analysis (**c**) of fluid-jet-stimulated glutamate secretion from utricular hair cells derived from *Pc-Lphn2*^*+/+*^ mice (black) or *Pc-Lphn2*^*fl/fl*^ mice (red) at P10 (*n* = 8). The magnitude of the glutamate secretion was characterized by ΔF/F_0_. Data are shown as means ± SEM. ***P < 0.001. Data were statistically analyzed using unpaired two-sided Student’s *t*-test. **d** Schematic diagram showing the detection of glutamate release in mouse utricular hair cells in response to force stimulation applied through magnetic beads. **e**, **f** Representative traces (**e**) and quantitative analysis (**f**) of glutamate secretion from individual utricular hair cell derived from P10 *Pc-Lphn2*^*+/+*^ mice (*n* = 12) or *Pc-Lphn2*^*fl/fl*^ mice (*n* = 10) in response to force applied through LPHN2-M-beads or control beads. Data are correlated to Supplementary information, Fig. S10c. Data are shown as means ± SEM. ***P < 0.001; ns no significant difference. Data were statistically analyzed using unpaired two-sided Student’s *t*-test. **g** Schematic diagram showing the detection of fluid-jet-stimulated Ca^2+^ response in utricular hair cells. **h**, **i** Representative traces (**h**) and quantitative analysis (**i**) of fluid-jet-stimulated Ca^2+^ signals in utricular hair cells derived from *Pc-Lphn2*^*+/+*^ mice (black) or *Pc-Lphn2*^*fl/fl*^ mice (red) at P10 (*n* = 6). The magnitude of the Ca^2+^ response was characterized by ΔF/F_0_. Data are shown as means ± SEM. ***P < 0.001. Data were statistically analyzed using unpaired two-sided Student’s *t*-test. **j** Schematic diagram showing the detection of Ca^2+^ signals in mouse utricular hair cells in response to force stimulation applied through magnetic beads. **k** Heatmaps showing the Ca^2+^ responses in individual utricular hair cells derived from *Pc-Lphn2*^*+/+*^ or *Pc-Lphn2*^*fl/fl*^ mice. *n* = 80, 20 and 20 for mCherry-labeled *Pc-Lphn2*^*+/+*^ cells (red box), mCherry-unlabeled *Pc-Lphn2*^*+/+*^ cells (orange box), and mCherry-labeled *Pc-Lphn2*^*fl/fl*^ cells (blue box), respectively. The color intensity indicates the magnitude of the calcium response characterized by ΔF/F_0_.

In response to mechanical stimulation during equilibrioception, VHCs may release neurotransmitters, such as glutamate, to transmit positional or motional information to spiral ganglion neurons (SGNs). We therefore expressed a genetically encoded glutamate sensor R^ncp^-iGluSnFR (R^ncp^-iGlu) in SGNs to monitor real-time glutamate release from utricular hair cells.^77^ Binding of glutamate to the R^ncp^-iGlu sensor induced conformation-based modulation of the protonation state of the chromophore, leading to a reduction in fluorescence intensity (Fig. 8a). Notably, compared with *Pou4f3-CreER*^*+/*−^;*Lphn2*^*+/+*^ utricular hair cells, those cells pretreated with LPHN2-specific inhibitor D11 or *Pou4f3-CreER*^*+/*−^;*Lphn2*^*fl/fl*^ hair cells displayed an ~45% decrease in glutamate release in response to fluid jet stimulation (Fig. 8b, c; Supplementary information, Fig. S10a, b). We further investigated LPHN2-mediated glutamate release using magnetic bead assay and found that repeated force application with 10 pN on utricular sensory epithelium through LPHN2-M-beads induced detectable glutamate release, which disappeared with force removal (Fig. 8d–f). As a negative control, the force applied by the Ctrl-beads did not induce any detectable glutamate secretion (Fig. 8e, f; Supplementary information, Fig. S10c). Moreover, force-stimulated glutamate secretion via LPHN2-M-beads was abrogated in the WT utricular explants pretreated with the LPHN2-specific inhibitor D11 or TMC1 inhibitor C14 or in the utricular explants derived from the *Pou4f3-CreER*^*+/*−^;*Lphn2*^*fl/fl*^ mice (Supplementary information, Fig. S10c–e). These results support a modulatory role of LPHN2 in converting mechanical stimuli into glutamate release.

Intracellular Ca^2+^ is a key element for neurotransmitter release and may play a pivotal role in balance sensation.^78–81^ We next examined the Ca^2+^ response downstream of LPHN2 in single hair cells after force stimulation. The Ca^2+^ signal in the LPHN2-expressing utricular hair cells, which were labeled with AAV-ie-*Lphn2pr*-mCherry, in response to force stimulation was recorded by a Fura-2 AM fluorescent probe. Consistent with the glutamate release results, the fluid-jet-stimulated Ca^2+^ response of utricular hair cells derived from *Pou4f3-CreER*^*+/*−^;*Lphn2*^*fl/fl*^ mice was ~45% weaker than that of the hair cells derived from the *Pou4f3-CreER*^*+/*−^;*Lphn2*^*+/+*^ littermates (Fig. 8g–i). A similar reduction in the fluid-jet-stimulated Ca^2+^ response was also observed in *Pou4f3-CreER*^*+/*−^;*Lphn2*^*+/+*^ utricular hair cells pretreated with D11 or with BAPTA (Supplementary information, Fig. S10f, g). Moreover, using magnetic bead assay, we found that ~35% of LPHN2-expressing hair cells derived from *Pou4f3-CreER*^*+/*−^;*Lphn2*^*+/+*^ utricle showed an increase in Ca^2+^ levels in response to force stimulation, and this Ca^2+^ signal was independent of the Gq signaling, as revealed by its insensitivity to the Gq inhibitor YM-254890 (Fig. 8j, k; Supplementary information, Fig. S10h, i). In contrast, the AAV-ie-*Lphn2pr*-mCherry-labeled utricular hair cells derived from the *Pou4f3-CreER*^*+/*−^;*Lphn2*^*fl/fl*^ mice showed no detectable Ca^2+^ signals in response to force application via LPHN2-M beads (Fig. 8j, k). We speculated that the relatively lower response rate of LPHN2-expressing hair cells in the Ca^2+^ assay compared to that in the fluid jet assay might be due to inefficient interaction between the LPHN2-M-beads and endogenous LPHN2. Consistent with this hypothesis, only ~53% of the randomly-selected utricular hair cells showed detectable Ca^2+^ signals in response to mechanical stimulation applied on tip link component CDH23, suggesting that binding efficiency of the magnetic beads was ~50% (Supplementary information, Fig. S10j, k).

Taken together, our results collectively indicate that the activation of LPHN2 by force stimulation induces Ca^2+^ signaling and neurotransmitter release in VHCs.

### Re-expression of LPHN2 in VHCs rescues the equilibrioception of LPHN2-deficient mice

To further assess the functional roles of LPHN2 specifically expressed in the vestibular system, we reintroduced LPHN2 expression in VHCs of *Pou4f3-CreER*^*+/*−^;*Lphn2*^*fl/fl*^ mice via AAV delivery. Our previous structural and functional analyses revealed that force sensation by aGPCRs was primarily mediated by the juxtamembrane GAIN domain.^16,20^ The Flag-tagged LPHN2-GAIN construct with the deletion of 523 N-terminal residues retained the mechanical sensitivity and showed dose-dependent Gs signaling in response to force stimulation with Flag-M-beads, and the response was comparable to that of full-length LPHN2 when expressed at similar levels.^72^ Therefore, we packaged the Flag-LPHN2-GAIN sequences into the AAV-ie-*Lphn2pr*-mCherry vector (mCherry following an IRES element was fused to the C-terminus of LPHN2-GAIN, referred to as AAV-ie-LPHN2) and delivered the virus into P3 *Pou4f3-CreER*^*+/*−^;*Lphn2*^*fl/fl*^ mice through a round window membrane injection using AAV-ie-*Lphn2pr*-mCherry as a negative control (Supplementary information, Fig. S11a). LPHN2-GAIN was specifically expressed in the utricular hair cells, but not in the brains, of the *Pou4f3-CreER*^*+/*−^;*Lphn2*^*fl/fl*^ mice 14 days after the administration of AAV-ie-LPHN2, as shown by western blotting and immunofluorescence analysis (Supplementary information, Fig. S11b, c). As a negative control, the *Pou4f3-CreER*^*+/*−^;*Lphn2*^*fl/fl*^ mice infected with AAV-ie-*Lphn2pr*-mCherry exhibited only specific utricular hair cell labeling but no detectable LPHN2-GAIN expression (Supplementary information, Fig. S11b, c).

**Fig. 9:**
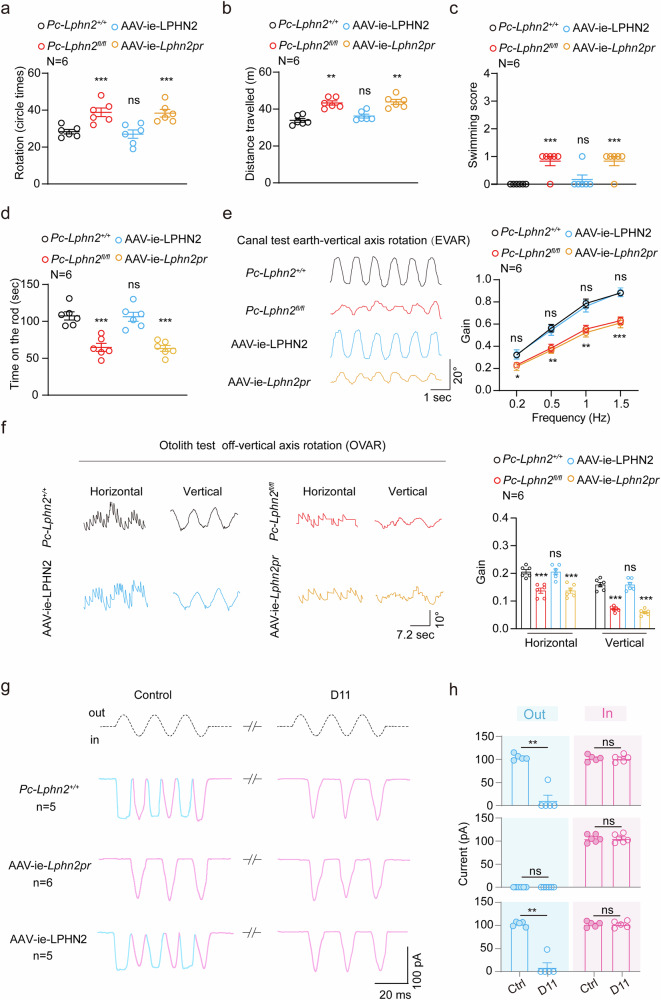
Re-expression of LPHN2 specifically in hair cells rescues vestibular function of LPHN2-deficient mice. **a**–**d** Quantification of the circling (**a**) and traveling activity (**b**) in the open-field test, swimming scores (**c**) and duration time on the rotating rod (**d**) of *Pc-Lphn2*^*+/+*^, *Pc-Lphn2*^*fl/fl*^, AAV-ie-LPHN2 mice and AAV-ie-*Lphn2pr* mice (*n* = 6 mice per group). Data are shown as means ± SEM. **P < 0.01; ***P < 0.001; ns no significant difference. Data were statistically analyzed using one-way ANOVA with Dunnett’s post hoc test. **e**, **f** Representative recording curves (left panels) and quantification of the VOR gain responses (right panels) of *Pc-Lphn2*^*+/+*^, *Pc-Lphn2*^*fl/fl*^, AAV-ie-LPHN2 mice and AAV-ie-*Lphn2pr* mice to earth-vertical axis (**e**) or off-vertical axis (**f**) rotation (*n* = 6 mice per group). Data are shown as means ± SEM. *P < 0.05; **P < 0.01; ***P < 0.001; ns no significant difference. Data were statistically analyzed using one-way ANOVA with Dunnett’s post hoc test. **g**, **h** Representative current traces (**g**) and quantitative analysis (**h**) of fluid-jet-stimulated MET responses in BAPTA-treated utricular hair cells derived from *Pc-Lphn2*^*+/+*^, AAV-ie-LPHN2 or AAV-ie-*Lphn2pr* mice in the absence or presence of 50 nM D11 (n = 5, 5 and 6 for *Pc-Lphn2*^*+/+*^, AAV-ie-LPHN2 or AAV-ie-*Lphn2pr* mice, respectively). Data are shown as means ± SEM. **P < 0.01; ns no significant difference. Data were statistically analyzed using paired two-sided Student’s *t*-test.

We next assessed the equilibration-related behaviors of the mice receiving LPHN2-GAIN gene delivery. Notably, the LPHN2-deficient mice treated with AAV-ie-LPHN2 exhibited significantly improved performance in all behavior studies related to equilibrioception, including forced swimming, open field and rotarod tests, and the performances were comparable to those of the WT mice (Fig. 9a–d). Moreover, the VOR gain values of the AAV-ie-LPHN2-treated *Pou4f3-CreER*^*+/*−^;*Lphn2*^*fl/fl*^ mice at all tested frequencies in both earth-vertical and off-vertical axis rotation tests recovered to levels comparable to those of the WT mice (Fig. 9e, f). As a negative control, the LPHN2-deficient mice treated with AAV-ie-*Lphn2pr*-mCherry showed no significant improvement in performance in any of the above tests (Fig. 9a–f). By analyzing MET response of the utricular hair cells, we found that *Lphn2*-deficient utricular hair cells infected with AAV-ie-LPHN2, but not those infected with AAV-ie-*Lphn2pr*-mCherry, showed significantly increased MET currents and restored D11 responsiveness, which were comparable to those of the WT utricular hair cells (Supplementary information, Fig. S11d, e). Furthermore, after BAPTA treatment to disrupt the tip links, the AAV-ie-LPHN2-treated *Pou4f3-CreER*^*+/*−^;*Lphn2*^*fl/fl*^ utricular hair cells reproduced a normal-polarity MET current, which was comparable to that found in the BAPTA-treated WT utricular hair cells and could be ablated by D11 administration (Fig. 9g, h). In contrast, the *Pou4f3-CreER*^*+/*−^;*Lphn2*^*fl/fl*^ vestibular hair cells treated with AAV-ie-*Lphn2pr*-mCherry showed no significant alterations in MET characteristics (Fig. 9g, h). Therefore, reintroduction of mechanosensitive LPHN2-GAIN in utricular hair cells rescued equilibrioception and restore D11-sensitive MET in LPHN2-deficient mice. These findings further indicated that force-activated LPHN2 in the vestibular system directly contributes to normal equilibrioception.

## Discussion

Despite its importance for daily life and motion in three-dimensional space, the molecular mechanism underlying the sense of balance is not fully understood. Studies have revealed that ion channels, such as TMC1/2, are potential key components of the MET process, which is essential for equilibrioception.^76,82,83^ In addition to ion channels, another group of membrane receptors, GPCRs, are known as direct sensors for vision, odor and touch.^30,50,84^ Although recent studies have shown that certain GPCR members, such as CELSR1, GPP156 and GPR126, contribute to vestibular development or the maintenance of VHC planar polarity,^85–87^ GPCRs are generally excluded from the equilibrioception process in the peripheral vestibular system due to their relatively slow kinetics in mediating secondary messenger pathways. In our current study, by screening potential mechanosensitive aGPCRs in vestibular system, we reveal that the force-sensing LPHN2 plays an important role in maintenance of normal balance. Importantly, while conditional knockout (cko) of *Lphn2* in VHCs impairs the balance behaviors of mice without affecting the morphology of utricular macula or hair cells, re-expression of LPHN2 in VHCs of *Lphn2*-deficient mice rescues equilibrioception. Specifically, both tamoxifen treatment and AAV delivery in *Pou4f3-CreER*^*+/*−^;*Lphn2*^*fl/fl*^ mice are limited to the vestibular organs through round window membrane injection, which results in no detectable effects on LPHN2 expression in tissues beyond the inner ear, especially the vestibular or somatosensory nuclei in the brainstem. These results collectively suggest a specific role for LPHN2 in regulating balance sensation in the peripheral vestibular system.

The LPHN2 actively participates in the MET process in VHCs, which is supported by the results of a fluid jet assay using VHCs derived from *Lphn2*-deficient mice or a specific reversible LPHN2 inhibitor. Unexpectedly, in contrast to the unique presence of LPHN2 near the tips of stereocilia in CHCs,^72^ LPHN2 is absent in stereocilia and is exclusively expressed at the apical surface of utricular hair cells. Importantly, through local interaction with TMC1, LPHN2 at the apical surface regulates a tip-link-independent normal-polarity MET current, which contributes to ~50% of the total MET currents in response to fluid jet stimulation. Therefore, our findings reveal a previously uncharacterized MET process at the apical surface of VHCs mediated by a GPCR–TMC1 functional coupling pair. Notably, previous studies have reported differing MET properties, including current amplitude and adaptation parameters, in utricular hair cells stimulated by a fluid jet (mechanical stimulation of both the hair bundle and apical surface) compared to those deflected by a stiff probe (stimulation of the hair bundle only), with potentially unknown underlying mechanisms.^71,88^ Our findings herein may provide a possible explanation for this discrepancy. Moreover, a tip-link-independent reverse-polarity MET current has been previously identified in CHCs; this current was reported to be regulated by Piezo2 and could be evoked following disruption of the sensory-transduction machinery.^69,89^ A similar reverse-polarity MET current is also present in saccular hair cells of mice during embryonic development but disappears as the normal MET current develops.^70^ Our findings, together with these data, suggest that the MET process in hair cells is not confined to the stereocilia but also involves other subcellular regions, such as the apical surface, where cells might sense mechanical forces from the extracellular matrix or fluid motion. Notably, there are two layers of extracellular matrix under the otoconial mass of utricle, namely, the upper otolithic membrane into which the stereocilia project and the lower columnar filament layer that directly contacts the apical surface of sensory epithelium.^90,91^ The amplitude of the force applied by extracellular matrices perpendicular to the utricular sensory epithelium may change in response to head tilting. The swelling pressure of these gel layers may also change as a result of modulation of the composition of the surrounding endolymphatic fluid.^91^ These changes may confer the natural mechanical stimuli on the apical surface of utricular hair cells. Our results also suggest that VHCs have MET characteristics different from those of CHCs, and these differences may be due to the substantial differences in hair bundle morphology between these two types of cells,^92^ or may be partially attributed to the specific distribution of mechanosensitive GPCRs and/or their potential regulation of the local cell membrane through downstream signaling. Although much remains unknown about the regulatory mechanism and physiological importance of MET currents at the apical surface of VHCs, as well as their potential relationship with MET currents at the stereocilia, the distinct MET processes at different subcellular locations suggest a greater complexity in equilibrioception compared to auditory perception. Integrated MET signals from both the stereocilia and the apical surface may provide more precise spatial resolution for positional sensation than is required for hearing.

In our previous study characterizing the functional roles of LPHN2 in auditory perception, we found that hair-cell-specific LPHN2-deficiency also causes hearing loss and impaired MET responses in CHCs.^72^ We revealed that LPHN2 and TMC1 may form a heteromer in the heterologous system, with the intracellular loop (ICL)1/2 (or potentially transmembrane helices (TM)1–4) of LPHN2 being in proximity to the C-terminus or TM10 region of TMC1. Further mechanistic study using intramolecular FlAsH-BRET assay showed that force applied on LPHN2 induces a separation between ICL2/3 and ICL4 of TMC1, which corresponds to a dissociation between the TM4–7 and TM8–9 ends. These conformational changes suggest the potential opening of the ion channel pore of TMC1, as revealed by the cryo-electron microscopy structure of the native *Caenorhabditis elegans* TMC-1.^93^ These results suggest that force sensation by LPHN2 could induce TMC1 activation through a conformational transition between these two membrane proteins. Intriguingly, LPHN2 regulates only the normal-polarity MET current but not the reverse-polarity current, suggesting that it can convert only a directional mechanical stimulus into enhanced TMC1 activity. Currently, the detailed underlying mechanism is unclear and requires further investigation. From the preliminary results of the STED imaging of LPHN2 in utricular hair cells, we observed a unique ring-like expression pattern of LPHN2 at the apical surface (Supplementary information, Fig. S11f). We speculate that the directional mechanosensitivity of LPHN2 might be related to this expression pattern as well as the interaction mode between LPHN2 and TMC1 at the apical surface; this is expected to help with the encoding of positional information. Further studies are required to investigate this issue. Notably, although the LPHN2–TMC1 pair is sufficient to convert extracellular stimuli into electrical signals in the heterologous system, we cannot exclude the potential involvement of other subunits or accessory proteins in vivo. For example, the conventional MET component LHFPL5 may be expressed at the apical surface of CHCs, while the chaperone receptor LGR6 used in the present study is endogenously expressed at the apical surface of VHCs.^75^ Whether these molecules are part of the apical surface MET machinery requires further investigation. Previous study has identified the expression of Piezo2 at the apical surface of cochlear hair cells, where it regulates the reverse-polarity MET current. Consistently, the *Piezo2*-cko and *Piezo1/2*-double cko mice showed mild auditory defects.^69^ In the vestibular hair cells, despite its expression, Piezo2 seems to be dispensable for vestibular functions since *Piezo2*-cko and *Piezo1/2*-double cko mice exhibited no obvious vestibular defects.^69^ In contrast, hair-cell-specific LPHN2-deficiency leads to balance dysfunction. Moreover, by an in vivo co-immunoprecipitation assay using mouse utricular lysates, we showed that LPHN2 does not directly interact with PIEZO2. We therefore speculate that PIEZOs may not participate in the modulation of LPHN2 on MET currents at the apical surface of vestibular hair cells. However, future studies using gene knockout mice are warranted to thoroughly clarify the relationship between PIEZOs or other mechanosensitive channels, with the LPHN–TMC1 signaling complex. Moreover, since LPHN2 is expressed in ~80% of utricular hair cells, it is of interest to explore whether and how the MET process might be regulated by other mechanosensitive GPCRs in the remaining 20% of LPHN2-negative hair cells. Future studies on these questions will provide in-depth insights into more functional roles of GPCRs in equilibrioception.

In addition to being primarily distributed at the apical surface of utricular hair cells (~86%), LPHN2 expression, though weak, was also observed at the bottom of a small fraction (~10%) of hair cells. Although we cannot exclude the possibility that LPHN2 has important function at the basolateral membrane of certain utricular hair cells, this does not affect the main conclusion of the current work. Moreover, the scRNA-seq datasets suggest that *Lphn2* might also be expressed in utricular supporting cells. However, using both a commercially available LPHN2 antibody and a transgenic knock-in mouse line, our results indicate that LPHN2, at the protein level, is specifically expressed in utricular hair cells and is not detectable in the supporting cells. Currently, we do not know the mechanism underlying the discrepancy of LPHN2 expression measured at the protein and at the mRNA levels. We speculated that this difference might be due to the regulation of protein abundance, such as the different protein degradation systems of the LPHN2 protein in supporting cells or hair cells, which needs further investigation. Beyond hearing and vestibular system, LPHN2 is widely expressed and regulates various other processes, such as synapse formation,^94^ heart development^95^ and vascular remodeling.^96^ The diverse expression pattern and multiple functions of LPHN2 are similar to those of PIEZOs. These mechanosensitive channels or receptors might respond to diverse types of forces (e.g., cellular compression, fluid shear stress, membrane tension) in different organs or tissues. However, further studies are required to investigate the function of the force sensation by LPHN2 in other tissues or cells.

Finally, many vertigo cases are caused by peripheral vestibular disorders such as vestibular neuronitis, Ménière’s disease and benign paroxysmal positional vertigo.^97^ Currently, the vestibular suppressants, which include anticholinergics, antihistamines, benzodiazepines and calcium channel antagonists, represent the primary medication for vertigo management.^98,99^ These vestibular suppressants mainly function through sedation or reducing nausea by inhibiting neural activity; but none of them directly addresses the vertigo problem by targeting the balance-sensing vestibular hair cells. The identification of the role of LPHN2 in equilibrioception may provide a potentially novel therapeutic avenue for treating vertigo. In particular, the therapeutic potential of selective inhibitors of LPHN2 that antagonize its force sensation, exemplified by D11, is expected to be probed in future studies using animal models of balance disorders. Moreover, it will be also of interest to test whether LPHN2 agonists might enhance or deteriorate the performance of animals in balance-related tasks.

Our current study indicates that LPHN2 expressed at the apical surface of utricular hair cells regulates a tip-link-independent MET current by converting force stimuli into TMC1 activity, which is required for the normal equilibrioception. However, we are aware that HEK293 cells express endogenously mechanosensitive ion channels, which may interfere with the measurement of the MET currents. Further studies using the liposome system or structural analysis of the force-sensing LPHN2–TMC1 complex would provide more insight into the mechanosensitivity of this GPCR–ion channel complex. Moreover, the mechanisms underlying different MET characteristics in CHCs and VHCs remain unknown. Further studies using more precise tools to differentiate the tip-link-dependent and -independent MET are required for the mechanistic investigations. In addition, although the magnetic-bead-delivered force in the present study is theoretically within the physiological range, its physiological significance was limited by the relatively low binding efficiency of the antibody-coated beads and the potential time delay in transmitting force. Further in-depth investigation of the functional roles of VHC-expressed LPHN2 in equilibrioception requires real-time recording of cellular responses in VHCs when stimulated by a natural force. Future in vivo two-photon calcium or neurotransmitter imaging in moving mouse models is expected to further elucidate the equilibrioception potential of LPHN2 or other GPCRs in a more physiological setting.

In conclusion, here we (1) identified a force-sensitive GPCR expressed at the apical surface of VHCs, (2) which senses force within a physiological range, (3) regulates a tip-link-independent MET process and converts force stimuli into neurotransmitter glutamate release. (4) Specific ablation of this receptor in hair cells severely impairs the balance of mice. These results conform to the criteria we propose for equilibrioception receptors and suggest that the mechanosensitive GPCR is required in equilibrioception.

## Materials and methods

### Mice

C57BL/6J WT mice were obtained from the Jackson Laboratory. *Lphn2*^*+/*−^ (S-KO-15867), *Lphn2*^*fl/fl*^ (S-CKO-17378), *Lphn3*^*+/*−^ (S-KO-09139), *Gpr133*^−*/*−^ (S-KO-07428), *Gpr126*^*fl/fl*^ (S-CKO-05939) and *Tmc1*^−*/*−^ (S-KO-18952) mice on a C57BL/6J background were purchased from Cyagen (China). *Cib2*^−*/*−^ and *Cib3*^−*/*−^ mice were generated by Cyagen as previously described^57^ and *Cib2*^−*/*−^;*Cib3*^−*/*−^ mice were obtained by crossing *Cib2*^−*/*−^ and *Cib3*^−*/*−^ mice. *Vlgr1*^−*/*−^ mice (*Vlgr1*/del7TM, Stock No. 009379) and *Atoh1-Cre* mice (Stock No. 011104) on a C57BL/6J background were obtained from the Jackson Laboratory. *Pou4f3-CreER* mice on a C57BL/6J background were generated by GemPharmatech (China). The *Pou4f3-CreER* line was generated by placing the *CreERT2* element downstream of the endogenous coding sequence of *Pou4f3*, which were separated by a P2A sequence. The *Pou4f3-CreER*^*+/*−^;*Lphn2*^*fl/fl*^ mice were generated by crossing *Pou4f3-CreER*^*+/*−^ mice with *Lphn2*^*fl/fl*^ mice. For activation of Cre recombinase in *Pou4f3-CreER*^*+/*−^ mice, the mice were treated with tamoxifen (75 mg/kg) dissolved in corn oil through round window membrane injection at P25 (left ear) and P26 (right ear) consecutively. For activation of Cre recombinase in *Pou4f3-CreER*^*+/*−^ mouse embryos, pregnant mice at 14 days post coitum were treated with 100 mg/kg tamoxifen supplemented with 37.5 mg/kg progesterone for 3 consecutive days through intraperitoneal injection. The *Atoh1-Cre*^*+/*−^;*Gpr126*^*fl/fl*^ mice were generated by crossing *Gpr126*^*fl/fl*^ and *Atoh1-Cre*^*+/*−^ mice. *Lphn2*^*mCherry*^ mice were generated by Cyagen with a 3× FLAG tag and a mCherry inserted at the N-terminus and C-terminus of LPHN2, respectively. All the mice were housed at the Shandong University Animal Care Facility under a 12-h light/12-h dark cycle. All the mice were group-housed in pathogen-free facilities with regulated temperature and humidity and given ad libitum access to food and water. Both male and female mice were used and were randomly assigned to experimental groups. All mouse care and experiments were reviewed and approved by the Animal Use Committee of Shandong University Cheeloo College of Medicine.

### Cell lines

Human embryonic kidney 293 (HEK293) cells were obtained from American Type Culture Collection and were cultured in DMEM supplemented with 10% fetal bovine serum, penicillin (100 IU/mL), and streptomycin (100 mg/mL) in 5% CO_2_ at 37 °C. The *PIEZO1-*knockout HEK293T cell was a gift from Prof. Bailong Xiao at Tsinghua University. Cell transfection was performed with Lipofectamine 2000 (Invitrogen) according to the manufacturer’s instructions.

### RNA extraction and RT-qPCR

Total RNA was extracted from the brain or utricle epithelium of WT mice using TRIzol reagent (Invitrogen). cDNA was synthesized using the RT-qPCR Kit (Toyobo, FSQ-101) and RT-qPCR was conducted using FastStart SYBR Green Master (Roche) on a LightCycler qPCR system (Bio-Rad). The relative mRNA levels of target genes, including *Lphn2* and *Lphn3*, were calculated using *Actb* as an internal control. For single-cell RT-qPCR, single hair cell from utricle sensory epithelium was aspirated into a patch pipette using the patch-clamp system under a microscope. The electrode tip containing the single utricular hair cell was then quickly broken into an RNase-free PCR tube for further analysis. RNA was extracted from single utricular hair cell (or AAV-ie-*Lphn2pr-*mCherry-labeled utricular hair cell) using Discover-sc® WTA Kit V2 (Vazyme, N711). cDNA synthesis, RT-qPCR and data analyses were performed as described above. We examined the expression of hair cell marker *Pou4f3* and only the cells expressing *Pou4f3* were selected for further data analysis. The sequences of all the primers used in the present study are listed in Supplementary information, Table S1.

### G protein dissociation BRET assay

To detect force-induced G protein activation through adhesion GPCRs, HEK293 cells were transiently co-transfected with plasmids encoding the receptors and corresponding BRET probes for different G proteins, including Gs and Gi3 subtypes, as previously described.^50,100^ Twenty-four h after transfection, the cells were distributed into a 96-well microplate at a density of 5 × 10^4^ cells/well. After incubation for another 24 h at 37 °C, the transfected cells were incubated with magnetic beads coated with polylysine (Ctrl-beads) or with Flag-M-beads. A magnetic system was used to apply force on the magnetic beads, which could be quantitatively determined through the following equation:${{{{F}}}}_{z}=\left(-1.2\times {10}^{-10}\right){\rm{exp}} \, \left(-104\cdot z\right)$, where z represents the distance between the beads and the magnetic-source in the z-direction, and could be manipulated to exert mechanical forces with varying strength. After stimulation with mechanical force for 5 min, the luciferase substrate coelenterazine 400a (5 μM, Interchim Cayman) was added into each well and the BRET signal was measured using a Mithras LB940 multimode reader (Berthold Technologies). BRET signal was calculated as the ratio of light emission at 510 nm to that at 400 nm. BRET signal changes due to force application were reported as ΔBRET.

For fluid-jet-stimulated G protein activation downstream of LPHN2, LPHN3 or GPR68, plasmids encoding these receptors and the corresponding BRET probes for different G proteins, including Gq, Gs and Gi3 subtypes, were transiently co-transfected in HEK293 cells. Twenty-four h after transfection, the cells were distributed into a 48-well microplate. After incubation for another 24 h at 37 °C, the transfected cells were incubated with the coelenterazine 400a (5 μM, Interchim Cayman) and the basal BRET signals were first recorded for 20 s using a Mithras LB940 multimode reader. The cells were then stimulated without or with a fluid jet from a pipette with a tip diameter of 100 μm. The fluid flow was controlled by a piezoelectric disk driven by a homemade 20× amplifier.^101^ A 10 V and 1 s square-wave stimulation was given to the piezoelectric disk to drive a fluid jet stimulation on the cell membrane. The cell microplate was immediately transferred back onto the same multimode reader and the BRET signals were recorded for at least 120 s. The peak BRET signal was recorded for data analysis.

### Open field test

To assess the locomotion behavior, the *Gpr133*^−*/*−^ mice, *Atoh1-Cre*^*+/*−^;*Gpr126*^*fl/fl*^ mice, *Lphn2*^*+/*−^ mice, *Lphn3*^*+/*−^ mice, *Pou4f3-CreER*^*+/*−^;*Lphn2*^*fl/fl*^ mice, *Vlgr1*^−*/*−^ mice*, Cib2*^−*/*−^*;Cib3*^−*/*−^ mice or their WT littermates at P40, or the *Pou4f3-CreER*^*+/*−^;*Lphn2*^*fl/fl*^ mice treated with AAV-ie-LPHN2 or control virus at P40 were subjected to open field test. After adapting for 15 min, the mice were placed in a 50 cm by 50 cm open field chamber in a room with a LED lighting set to 30 lux. An overhead camera (Hikvision) was used to record the traveling tracks and circulating behaviors of the mice within a 2-min timeframe and a 10-min timeframe, which were further analyzed by the Smart 3.0 software.

### Rotarod test

The *Lphn2*^*+/*−^ mice, *Lphn3*^*+/*−^ mice, *Pou4f3-CreER*^*+/*−^;*Lphn2*^*fl/fl*^ mice, *Cib2*^−*/*−^*;Cib3*^−*/*−^ mice or their WT littermates at P40, or the *Pou4f3-CreER*^*+/*−^;*Lphn2*^*fl/fl*^ mice treated with AAV-ie-LPHN2 or control virus at P40 were placed on a rotating rod (Harvard Apparatus) at a speed of 4 rpm for 3 min. Before the test, all the mice underwent training for 4 consecutive days with 3 trials per day. On the day of rotarod test, the rotating speed of the rod accelerated at a rate of 0.1 rpm/s and the time when the mice fell from the rotarod was recorded. The mice were tested over five consecutive trials, with a 5-min resting period between each trial. The average duration time on the rotarod for each mouse was used for analysis.

### Forced swim test

The *Lphn2*^*+/*−^ mice, *Lphn3*^*+/*−^ mice, *Pou4f3-CreER*^*+/*−^;*Lphn2*^*fl/fl*^ mice, *Cib2*^−*/*−^*;Cib3*^−*/*−^ mice or their WT littermates at P40, or the *Pou4f3-CreER*^*+/*−^;*Lphn2*^*fl/fl*^ mice treated with AAV-ie-LPHN2 or control virus at P40 were placed in a round plastic tub with a diameter of 20 cm, which was filled with water to a depth of 20 cm at a temperature of 26 °C. The swimming behavior for each mouse was recorded for 1 min. Swimming ability was scored according to the 0–3 scoring system as follows: 0 = normal swimming; 1 = irregular swimming; 2 = immobile floating; 3 = underwater tumbling.

### VOR

The eye movement of *Lphn2*^*+/*−^ mice, *Lphn3*^*+/*−^ mice, *Pou4f3-CreER*^*+/*−^;*Lphn2*^*fl/fl*^ mice, *Cib2*^−*/*−^*;Cib3*^−*/*−^ mice or their WT littermates at P40, or the *Pou4f3-CreER*^*+/*−^;*Lphn2*^*fl/fl*^ mice treated with AAV-ie-LPHN2 or control virus at P40 was evaluated by VOR experiment. The eye position and head signals were recorded by a binocular video oculography system (Gianttek, GAT-MVOR942). The mice were placed in a custom-built holder and fixed on a rotating platform (300-mm diameter) equipped with infrared illumination and video-detection facilities. The motion of platform was driven by a stepper motor (Melike, China), providing stimuli of sinusoidal counter rotation following designed rotation modes. For the earth-vertical axis rotation test, the mouse wearing an eye tracker was sinusoidally rotated at the frequency of 0.2, 0.5, 1, or 1.5 Hz with peak velocities of ±40°/s for no less than 90 s. For the off-vertical axis rotation test, the platform was tilted 17° off-vertical axis with the mouse starting from a nose-down position. The mouse was rotated at a constant velocity of 50°/s for at least 10 completed cycles in randomized clockwise or counterclockwise direction. Eye position data were analyzed using OpenCV 4.0.0 (Open Source Computer Vision Library, Intel) and MatLab (MathWorks, Natick, MA). The Gain data at different testing frequencies, which were defined as the ratio of eye velocity and head velocity, were calculated by performing Fast Fourier Transform on the de-saccaded eye velocity signal and head rotation signal.

### VEMP

*Pou4f3-CreER*^*+/*−^*Lphn2*^*fl/fl*^ mice and their WT littermates at P40 were subjected to click-evoked VEMP recording initiated with simultaneous recording of electromyography potentials as previously described.^102,103^ Briefly, the mouse was anesthetized with ketamine (100 mg/kg) and xylazine (12 mg/kg) intraperitoneally and placed in a custom-built holder, with its neck hyperextended and stabilized with suspension wire fixed behind the front teeth. A platinum needle electrode was inserted into the neck extensors. A reference electrode and a ground electrode were placed on the cervico-occipital region at the midline and on the back, respectively. A stimulus intensity of 100 dB nHL was applied to the mouse and the VEMP was determined by the presence of a positive (P1)-negative (N1) waveform at 6–9 ms latency as well as the presence of a preceding negative wave before P1. The reproducibility was verified by performing at least 5 runs to yield reproducible peaks. The peak latencies and amplitudes were measured and analyzed using Neuro-Audio (Neurosoft).

### Immunofluorescence staining and confocal microscopy

For wholemount immunostaining, the WT, *Pou4f3-CreER*^*+/*−^;*Lphn2*^*fl/fl*^, and the *Pou4f3-CreER*^*+/*−^;*Lphn2*^*fl/fl*^ mice treated with AAV-ie-LPHN2 or control virus were decapitated after anesthesia with ketamine (100 mg/kg) and xylazine (25 mg/kg) administered intraperitoneally. The head was sprayed with 70% (v/v) ethanol, and the skull was opened to expose the inner ear. The inner ears were transferred into dissection solution and then fixed with 4% paraformaldehyde overnight at 4 °C. The utricle or saccule was dissected as a whole and treated with 0.5 M EDTA for 1–3 h at room temperature for decalcification. After three washes with PBS, the utricle or saccule was permeabilized and blocked with PBS containing 0.5% Triton-X and 5% goat serum for 2 h. The utricle or saccule wholemount was incubated with primary antibodies overnight at 4 °C, followed by incubation with secondary antibodies for 1–2 h at room temperature. The samples were washed three times with PBS, mounted in ProLong Gold Antifade (Thermo Fisher Scientific), and subjected to fluorescence microscopic analysis using a Leica SP8 STED laser confocal microscope (Leica Microsystems) with a 100× (1.4 NA) oil immersion objective (for LPHN2 imaging in the optical sectioning microscopy) or a LSM980 laser scanning confocal microscope system (ZEISS) with a 20× (0.8 NA) objective (for imaging of utricle or saccule wholemounts), a 40× (1.2 NA) water immersion objective (for imaging of utricle cryosections), or a 63× (1.4 NA) oil immersion objective (for the other imaging unless otherwise specified). Image acquisition and merging were performed using LAS X (for Leica) or ZEISS Zen (for ZEISS) software. The pinhole size was set to 1 Airy Unit. The optical slice thickness was 0.5 μm. Both imaging systems can achieve a lateral resolution of 120 nm and an axial resolution of 350 nm according to the manufacturers’ instructions.

For the cryosection immunostaining, the decalcified inner ear was sequentially dehydrated using sucrose solutions with a concentration gradient (15, 20 and 30% in PBS). The inner ear was embedded in Tissue-Tek OCT compound (Sakura Fintek, USA) and 6-μm sections were cut. The utricle slices were incubated with blocking buffer at room temperature for 2 h and were processed for immunostaining and fluorescence microscopic analysis.

To investigate the subcellular localization of TMC1 in HEK293 cells, the cells were transiently transfected with plasmids encoding TMC1 and LGR6. Twenty-four h after transfection, the cells were seeded into 35-mm fibronectin-coated glass-bottom dish at a density of 5 × 10^5^ cells/dish. After another 24 h, the cells were fixed and processed for immunostaining and fluorescence microscopic analysis.

Primary antibodies used in the present study were as follows: rabbit anti-LPHN2 (Sigma-Aldrich, HPA043447, 1:200), mouse anti-Myosin 7a (DHSB, 13-1-C, 1:800), mouse anti-SOX2 (Santa Cruz Biotechnology, sc-365823, 1:200), Phalloidin-iFluor 594 (Abcam, ab176757, 1:1000), Phalloidin-iFluor 488 (Abcam, ab176753, 1:5000), Phalloidin-iFluor 647 (Abcam, ab176759, 1:5000), mouse anti-spectrin α (Sigma-Aldrich, ZRB2080, 1:100), mouse anti-Pou4f3 (Santa Cruz Biotechnology, sc-390780, 1:400), mouse anti-acetylated tubulin (Sigma-Aldrich, T7451, 1:400), Rabbit anti-PCDH15 antibody (Abcam, ab202560, 1:100), Mouse anti-CDH23 antibody (Santa Cruz SC-166066, 1:100; this antibody recognizes the extracellular N-terminal sequence (residues 141–450) of CDH23), Rabbit anti-TMC1 antibody (Merck, ABN1649, 1:100), Rabbit anti-TMC2 antibody (Abcam, ab200039, 1:100), Rabbit anti-LHFPL5 antibody (Thermo Fisher Scientific, PA5-23919, 1:100), Rabbit anti-TMIE antibody (Thermo Fisher Scientific, PA5-58330, 1:100), and Mouse anti-FLAG antibody (Sigma, F1804, 1:100). Secondary antibodies were as follows: Alexa Fluor Plus 488 Goat anti-Mouse IgG (Invitrogen, A32723), Alexa Fluor Plus 555 Goat anti-Mouse IgG (Invitrogen, A32727), Alexa Fluor Plus 488 Goat anti-Rabbit IgG (Invitrogen, A32731), and Alexa Fluor Plus 594 Goat anti-Rabbit IgG (Invitrogen, A32740).

### RNAscope in situ hybridization

To detect LPHN2 expression in utricular hair cells, RNAscope in situ hybridization was performed on utricle wholemounts using RNAscope 2.5 HD detection kit (ACD Bio 322360). The samples were treated sequentially with hydrogen peroxide (10 min at room temperature), Target Retrieval solution (5 min at 95 °C), and Protease III (11 min at 40 °C). *Lphn2* Probe (ACD Bio 319341) hybridization and signal amplification were performed according to the manufacturer’s instructions. The samples were washed with PBS and blocked with PBS containing 5% goat serum for 1 h at room temperature. After incubation with mouse anti-Myosin7a antibody and Alexa Fluor Plus 555 Goat anti-Mouse secondary antibody, the samples were subjected to confocal microscopic analysis using an LSM980 laser scanning confocal microscope system (ZEISS). The positive control and negative control experiments were performed in parallel using the probes targeting *Mm-Ppib* (ACD Bio 320881) or bacterial *dapB* probe (ACD Bio 320871), respectively.

### Adeno-associated virus preparation and injection

The AAV-ie-*Lphn2pr*-mCherry (3.5 × 10^13^ genomic copies per mL) and AAV-ie-LPHN2 (3.0 × 10^13^ genomic copies per mL) were generated by OBiO Technology (China). Both viruses were modified from the original AAV-ie vectors.^104^ Briefly, for the construction of AAV-ie-*Lphn2pr*-mCherry, the original CAG promoter in the AAV-ie was replaced with the promoter region (from –800 to 0) of *Lphn2*, which directs the expression of mCherry and could be used for the labeling of LPHN2-expressing utricular hair cells. For in vivo rescue experiment, the LPHN2-GAIN sequence with an N-terminal 3× Flag tag was inserted into the AAV-ie-*Lphn2pr*-mCherry vector. All viral vectors were aliquoted and stored at –80 °C until use.

For virus injection, *Pou4f3-CreER*^*+/*−^;*Lphn2*^*+/+*^ or *Pou4f3-CreER*^*+/*−^;*Lphn2*^*fl/fl*^ mice at P3 were placed in an ice bath until loss of consciousness (within 3 min) and then moved to an ice pad for surgical procedures. Injections were performed through the round window membrane of left ear of mouse with a glass micropipette (25 μm) at a speed of 1 μL virus/min controlled by a micromanipulator UMP3 UltraMicroPump (World Precision Instruments). A total volume of 0.8 μL of the virus was injected and then the incision was closed using veterinary tissue adhesive (Millpledge Ltd, UK). The mice were placed on a 38 °C warming pad until fully awake.

### Single-cell Ca^2+^ imaging

The utricle explants derived from *Pou4f3-CreER*^*+/*−^;*Lphn2*^*+/+*^ or *Pou4f3-CreER*^*+/*−^;*Lphn2*^*fl/fl*^ mice were attached to 12-mm round glass coverslips, which were pre-coated with polylysine and placed in 35-mm dishes. Utricle explants were incubated with imaging buffer (10 mM D-glucose, 150 mM NaCl, 5 mM KCl, 1.3 mM MgCl_2_, 1.2 mM NaH_2_PO_4_, 3 mM CaCl_2_, 20 mM HEPES, pH 7.4) containing 2.5 μM Fura-2 AM and 0.05% Pluronic F-127 (Life Technologies) for 15 min at 37 °C. After washing with imaging buffer, the utricle explants were incubated with fresh imaging buffer containing LPHN2-M-beads, CDH23-M-beads or Ctrl-beads for 20 min at 37 °C to enable the association of the antibody-coated beads with the target proteins.

Then, the dish containing the utricle explants was mounted into an Olympus IX71 microscope equipped with a FluoCa BioHD CMOS camera and a pE-340fura illumination system (coolLED). The utricular hair cells labeled by AAV-ie-*Lphn2pr*-mCherry were selected for Ca^2+^ imaging. The F340/F380 ratio of selected cells were recorded while the magnetic force was applied on the LPHN2-M-beads, CDH23-M-beads or Ctrl-beads. Data are recorded and analyzed using the VisiFLUOR Fluorescence Ratio Imaging System (Visitron Systems).

For fluid-jet-stimulated calcium imaging, the utricle explant preloaded with Fura-2 AM was placed in a recording chamber mounted on an upright microscope (Olympus) (with a 63× water-immersion objective) that was equipped with a FluoCa BioHD CMOS camera and a pE-340fura illumination system (coolLED). A series of 200-ms pulse signals of 10 V were delivered to the piezoelectric disk to drive the fluid jet stimulation from a pipette with a tip diameter of 5–10 μm. The interval between stimuli is 40 s. Data are collected and analyzed as described above.

### Western blotting assay

The brain, cochlea, vestibule or skin from WT, *Lphn2*^*+/*−^, *Lphn3*^*+/*−^, *Lphn3*^−*/*−^, *Pou4f3-CreER*^*+/*−^;*Lphn2*^*+/+*^, *Pou4f3-CreER*^*+/*−^;*Lphn2*^*fl/fl*^, *Lphn2*^−*/*−^ embryos, *Gpr133*^−*/*−^ mice, *Atoh1-Cre*^*+/*−^;*Gpr126*^*fl/fl*^ mice and *Vlgr1*^−*/*−^ mice or HEK293 cells transfected with LPHNs or empty plasmid pcDNA3.1 were collected with NP40 lysis buffer containing phosphatase and protease inhibitors. After centrifugation at 13,000 rpm for 15 min at 4 °C, the proteins were collected from the supernatant. Protein concentration was determined by BCA assay and the proteins were subjected to western blotting. Antibodies used in this study include anti-LPHN2 rabbit antibody (Santa Cruz Biotechnology, sc-514197, 1:500), anti-LPHN3 (Thermo Fisher Scientific, PA5-114759, 1:1000), anti-ACTIN (ZSGB-BIO, TA-09,1:2000), anti-GPR133 rabbit antibody (Invitrogen, PA5-106839, 1:1000), anti-GPR126 rabbit antibody (Proteintech, 17774-1-AP, 1:1000), anti-VLGR1 goat antibody (Santa Cruz, sc-21252, 1:1000), anti-FLAG mouse antibody (Sigma, F1804, 1:1000), anti-TMC1 Rabbit antibody (Abcam, ab199949, 1:1000), anti-Piezo2 Rabbit antibody (Novus, NBP1-78538SS, 1:1000).

### Co-immunoprecipitation

The utricles from WT mice were ground and lysed with NP40 lysis buffer containing phosphatase and protease inhibitors. The lysates were immunoprecipitated using LPHN2-antibody-conjugated agarose overnight at 4 °C. After washing with PBS, the immunoprecipitated proteins were subjected to electrophoresis and western blotting analysis using specific antibodies against TMC1.

### Hair cell electrophysiology

AAV-ie-*Lphn2pr*-mCherry was delivered into *Pou4f3-CreER*^*+/*−^;*Lphn2*^*+/+*^ or *Pou4f3-CreER*^*+/*−^;*Lphn2*^*fl/fl*^ mice at P3. The organs of utricle were isolated at P10 and the mCherry-labeled utricular hair cells were selected for electrophysiological studies. Excised utricle or saccule plants were placed in a recording chamber mounted on an upright microscope (Olympus), and visualized using a 63× water-immersion objective and infrared differential interference contrast. MET currents were recorded using fluid jet assay as described previously.^101^ Briefly, currents were evoked using a fluid jet from a pipette (tip diameter of 5–10 μm) positioned facing the staircase side of the hair bundle at a distance of ~5 μm at room temperature. The fluid jet was driven by a 40-Hz sinusoidal voltage to deflect the hair bundles. The fluid flow was controlled by a 27-mm-diameter piezoelectric disk driven by a homemade 20× amplifier that precisely outputs analog driving voltage. The command signal was generated by an amplifier (EPC10, HEKA) controlled by the PatchMaster software with a holding potential of –64 mV. Patch pipettes were pulled from borosilicate glass using a micropipette puller (P-97, Sutter Instrument Co.) to a resistance of 3–5 MΩ. The pipette solution contained 135 mM KCl, 2.5 mM MgCl_2_, 5 mM EGTA, 2.5 mM K_2_ATP, 0.1 mM CaCl_2_, 5 mM HEPES, adjusted to a pH of 7.4 with KOH. The external solution contained 137 mM NaCl, 5.8 mM KCl, 0.9 mM MgCl_2_, 1.3 mM CaCl_2_, 0.7 mM NaH_2_PO_4_, 10 mM HEPES and 5.6 mM D-glucose, vitamins at 1:100 and amino acids at 1:100 (Invitrogen, USA), adjusted to a pH of 7.4 with NaOH. For MET current stimulated by a stiff glass probe, the utricular hair cells were recorded using the whole-cell voltage clamp as previously described.^8,105^ Briefly, hair bundles were deflected with a glass pipette mounted on a P-885 piezoelectric stack actuator (Physik Instrument). The actuator was driven with voltage steps and the current-displacement curves were fitted with a second-order Boltzmann model. For D11 treatment, a 1-μM stock solution of D11 was prepared, which was added to the bath solution to obtain a final concentration of 50 nM D11. This solution was bath applied using a gravity fed application system at a perfusion rate of 2–3 mL/min and reached the recording chamber (with a volume of 2 mL) to replace the D11-free solution. Electrophysiological recording of utricular or saccular hair cells was performed 2 min after perfusion. To wash away the D11, we switched the D11-containing bath solution to the regular D11-free solution in the gravity fed application system and recorded the currents after continuous perfusion of 3 min. For recording the reverse-polarity MET currents, BAPTA was also added to the bath solution using the gravity fed application system at a perfusion rate of 2–3 mL/min to make a final concentration of 5 mM. To record the Na^+^ current in utricular hair cells, a pipette solution (137 mM CsCl, 5 mM EGTA, 5 mM HEPES, 2.5 mM Na_2_-ATP, 0.1 mM CaCl_2_, 3.5 mM MgCl_2_, adjusted to a pH of 7.4 with CsOH) was used.

For the electrophysiology in the heterologous system, HEK293 cells transfected with different combinations of plasmids encoding LGR6, TMC1 or LPHN2 were plated onto coverslips coated with polylysine. The single-channel currents in HEK293 cells were recorded 24–48 h after transfection. Cell-attached patch recordings were performed in extracellular solution (140 mM NaCl, 5 mM KCl, 2.5 mM CaCl2, 1 mM MgCl_2_, 10 mM D-glucose, 10 mM HEPES, pH 7.4 with NaOH) using 6–8 MΩ resistance electrodes filled with intracellular solution (140 mM CsCl, 5 mM EGTA and 10 mM HEPES, pH 7.4 with KOH). The current-voltage (I-V) curves of the spontaneous currents were plotted and fitted to the response amplitude measured from –80 to +80 mV in 20-mV increments. The single-channel conductance was obtained through linear fitting of the current-voltage plots. The inhibitors (D11 (50 nM) or C14 (1 µM)) were added to the bath solution and the channel opening was recorded as described above. For the force-induced single-channel currents, the cells were incubated with fresh extracellular solution containing LPHN2-M-beads or Ctrl-beads for 20 min at 37 °C to enable the association of the antibody-coated beads with the target proteins. Then, the cell-attached patch recordings were performed while the magnetic force was applied on the LPHN2-M-beads or Ctrl-beads. The single-channel data were processed with Clampfit v.10.6 (Molecular Devices). At least 10 s of recordings for each group were analyzed to characterize the consecutive channel openings. All-point histograms were fitted by a sum of two Gaussians to calculate the open probability (Po). The ratio of the area whose peak corresponded to a larger current, referred to as the ‘open state’, to the total area under the entire Gaussian distribution was considered Po. To explore the potential effect of C14 on CNGA3, whole-cell recordings were performed on HEK293 cells transfected with CNGA3 using 6–8 MΩ resistance electrodes filled with intracellular solution (150 mM KCl, 3 mM MgCl_2_, 5 mM EGTA, 10 mM HEPES, pH 7.2 with KOH, supplemented with 2 μM cGMP). The corresponding I-V curves were plotted as described above for TMC1 currents.

To record the whole-cell currents in the heterologous system, the transfected HEK293 cells were incubated in the recording solution containing 145 mM NaCl, 5.8 mM KCl, 0.9 mM MgCl_2_, 1.3 mM CaCl_2_, 0.7 mM NaH_2_PO_4_, 10 mM HEPES and 5.6 mM D-glucose (adjusted to a pH of 7.4 with NaOH). The pipette solution contains 142 mM KCl, 3.5 mM MgCl_2_, 1 mM EGTA, 2.5 mM MgATP, 0.1 mM CaCl_2_, 5 mM HEPES, adjusted to a pH of 7.4 with KOH. The whole-cell current recording was performed while a 10 V and 400 ms square-wave stimulation was given to the piezoelectric disk to drive a fluid jet stimulation on the cells.

### Virtual screening for TMC1 inhibitors

The three-dimensional structure of mouse TMC1 was modeled using AlphaFold2.^106^ The Sitemap program^107^ from the Schrödinger software package was employed to predict the binding sites of ligands in the constructed TMC1 structure. The grid spacing was set to 0.7 Å, and points that border on too many assigned philic points were excluded. Each pocket required at least 15 site points, and top three potential binding pockets were identified and analyzed. Based on the prediction results, the top scoring binding site was selected for further molecular docking-based virtual screening using the Maestro (version 13.4, Schrödinger LLC) program from the Schrödinger software package. The TMC1 structure was prepared using the Protein Preparation Wizard^108^ module, which involves procedures such as adding hydrogen atoms, assigning hydrogen bonds, and performing restrained optimization with default settings. Then, the Receptor Grid Generation module was used to generate corresponding grid files for the predicted ligand-binding sites. Next, the Ligand Docking module was employed to dock small molecules from our compound library (1,000,000 compounds), and the XP (extra precision)^109^ mode was utilized to evaluate the scoring of compounds with TMC1. Based on the scoring results, the top 5000 small molecules were selected for further screening of key residues’ interactions. Subsequently, the top 300 compounds were subjected to cluster analysis using the ‘Clustering Molecules’ protocols integrated in Pipeline Pilot, version 7.5 (Pipeline Pilot; Accelrys SoftwareInc.). Finally, 60 candidate compounds were manually selected for activity testing by electrophysiology recording assay.

### Glutamate secretion detection

To characterize the glutamate secretion from utricular hair cells, AAV-ie-*hSyn*-R^ncp^-iGluSnFR (4 × 10^13^ genomic copies per mL) were delivered to *Pou4f3-CreER*^*+/*−^;*Lphn2*^*+/+*^ or *Pou4f3-CreER*^*+/*−^;*Lphn2*^*fl/fl*^ mice at P3. A total volume of 0.8 μL of the AAV-ie-*hSyn*-R^ncp^-iGluSnFR virus was injected and then the incision was closed using veterinary tissue adhesive (Millpledge Ltd, UK). The utricle was isolated at P10 and the explants were incubated with fresh imaging buffer containing LPHN2-M-beads, CDH23-M-beads or Ctrl-beads for 20 min at 37 °C to enable the association of the antibody-coated beads with the target proteins. Widefield imaging was performed on an inverted Olympus IX71 microscope equipped with a 200-W metal halide lamp (PRIOR Lumen), 60× objective, and a 16-bit QuantEM 512SC electron-multiplying CCD camera (Photometrics). A filter set of 620/60 nm (emission) and 570 nm (dichroic) was used for recording cells labeled by AAV-ie-*hSyn*-R^ncp^-iGluSnFR. The selected cells were recorded while the magnetic force was applied on the LPHN2-M-beads, CDH23-M-beads or Ctrl-beads. Data were recorded and analyzed using the VisiFLUOR Fluorescence Ratio Imaging System (Visitron Systems). To detect the fluid-jet-stimulated glutamate secretion, the utricle explant expressing the glutamate reporter was placed in a recording chamber mounted on an upright Olympus microscope. A series of 200-ms pulse signals of 10 V were delivered to the piezoelectric disk to drive the fluid jet stimulation from a pipette with a tip diameter of 5–10 μm. The interval between stimuli is 40 s. Data were recorded and analyzed as described above.

### Statistical analysis

All data in the present study are presented as means ± SEM, with the number of mice or individual experiments indicated in the Figures or Figure legends. Paired or unpaired two-tailed Student’s *t*-test was used for comparisons between two groups. Differences among multiple groups with one or two variables were determined using one-way or two-way ANOVA respectively, followed by Dunnett’s post hoc tests. Statistical analysis was performed using GraphPad Prism 9 software. Significance was defined as *P < 0.05, **P < 0.01, ***P < 0.001. No methods were used to determine whether the data met assumptions of the statistical approach. No power analysis was performed to determine the sample size. The sample size of mice in each experiment was determined based on experience from previous studies in our lab.

## Supplementary information

Supplementary Figure1

Supplementary Figure2

Supplementary Figure3

Supplementary Figure4

Supplementary Figure5

Supplementary Figure6

Supplementary Figure7

Supplementary Figure8

Supplementary Figure9

Supplementary Figure10

Supplementary Figure11

Supplementary TableS1

Supplementary TableS2

## Data Availability

All data are available upon reasonable request from the corresponding authors.
