# Supplementary material for: A force-sensitive adhesion GPCR is required for equilibrioception
Source: Cell Res. 2025 Feb 18;35(4):243–64. doi: 10.1038/s41422-025-01075-x (PMC11958651; doi:10.1038/s41422-025-01075-x)
Supplement: Supplementary file 1 — Supplementary Figure1 [file 41422_2025_1075_MOESM1_ESM.pdf]

Supplementary information, Figure S1

a

Receptor Ligand	G Protein	Physiological significance	Assay	References
ADGRE2/EMR2	N/A	Enhanced Degranulation of Mast Cells from Patients.	Vibratory urticaria,in vitro cell-based system	[1]
ADGRE5/CD97	N/A	Cell adhesion	Blood flow/animal model	[2]
ADGRG1/GPR56	G13/Gi	Hemostasis and thrombosis	Platelet Adhesion to Immobilized Collagen Flow Chamber Assays	[3]
ADGRG5/GPR114	Gs	N/A	in vitro cell-based system	[4]
ADGRG6/GPR126	Gq/G11	Schwann cell development	in vitro cell-based system	[5]
ADGRL1/LPHN1/CIRL	Gi	Increasing the mechanosensitivity of neurons	Drosophila model	[6]
ADGRD1/GPR133	Gs	N/A	in vitro cell-based system	[7]

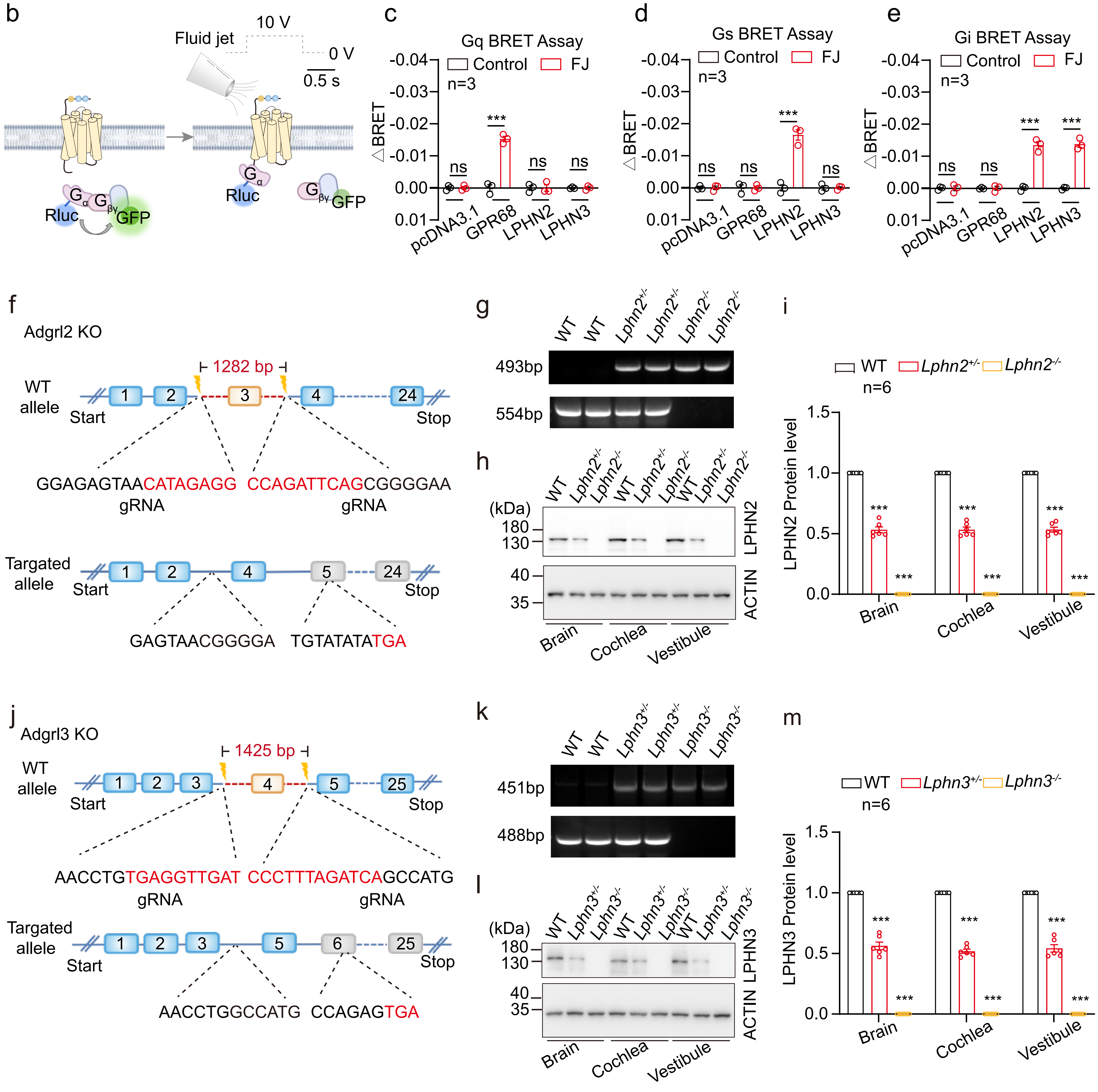

Figure S1. Generation and genotyping of *Lphn2* or *Lphn3* gene knockout mice

(**a**) Summary of the molecular characteristics and physiological significance of the reported mechanosensitive adhesion GPCRs[1-7].

(b) Schematic diagram showing the detection of fluid jet (referred to FJ)-stimulated G protein activation in HEK293 cells transfected with plasmids encoding GPCRs and G protein BRET probes. A constitutive BRET signal could be detected between the Rluc inserted into Gα subunit and the GFP tagged to Gγ, which is decreased upon GPCR activation and G protein dissociation. A one-time step fluid jet (10 V and 1 s square-wave stimulation) was used to drive the mechanical stimulation on the cell membrane.

(c-e) FJ-stimulated Gq **(c)** Gs **(d)** or Gi **(e)** activation in HEK293 cells overexpressing GPR68 or LPHN2 measured by G protein dissociation BRET assay (n = 3). HEK293 cells transfected with the empty vector pcDNA3.1 and G protein probes were used as the negative control. Data are shown as mean ± SEM. ***P < 0.001; ns, no significant difference. FJ-stimulated HEK293 cells compared with control cells. Data were statistically analyzed using unpaired two-sided Student's *t* test.

(f) Schematic representation of the generation of *Lphn2* knockout mice by CRISPR-Cas9 strategy. A deletion of 1282 bp was introduced between the exons 2 and 4 of the *Lphn2* gene to induce a premature translation termination.

(g) Genotyping PCR results showing amplified fragments derived from *Lphn2*-deficient mice and WT mice. The E15-E18 embryonic samples of $Lphn2^{-/-}$ mice were used for genotyping PCR.

(h, i) Western blotting (**h**) and quantitative analysis (**i**) of the endogenous expression of LPHN2 in the membrane fractions of brain, cochlea and vestibule isolated from the WT or *Lphn2*-deficient mice (n = 6). The E15-E18 embryonic samples of $Lphn2^{-/-}$ mice were used for western blotting analysis. Data are normalized to the expression levels of LPHN2 in respective organs of WT mice.) Data are shown as mean ± SEM. ***P < 0.001. *Lphn2* knockout mice compared with WT mice. Data were statistically analyzed using one-way ANOVA with Dunnett's post hoc test**.**

(j) Schematic representation of the generation of *Lphn3* knockout mice by CRISPR-Cas9 strategy. A deletion of 1425 bp was introduced between the exons 3 and 5 of the *Lphn3* gene to induce a premature translation termination.

(k) Genotyping PCR results showing amplified fragments derived from *Lphn3*-deficient mice and their WT littermates.

(l, m) Western blotting (**l**) and quantitative analysis (**m**) of the endogenous expression of LPHN3 in the membrane fractions of brain, cochlea and vestibule isolated from the WT or *Lphn3*-deficient mice (n = 6). Data are normalized to the expression levels of LPHN3 in respective organs of WT mice. Data are shown as mean ± SEM. ***P < 0.001. *Lphn3* knockout mice compared with WT mice. Data were statistically analyzed using one-way ANOVA with Dunnett's post hoc test**.**
