# Supplementary material for: A force-sensitive adhesion GPCR is required for equilibrioception
Source: Cell Res. 2025 Feb 18;35(4):243–64. doi: 10.1038/s41422-025-01075-x (PMC11958651; doi:10.1038/s41422-025-01075-x)
Supplement: Supplementary file 2 — Supplementary Figure2 [file 41422_2025_1075_MOESM2_ESM.pdf]

Supplementary information, Figure S2

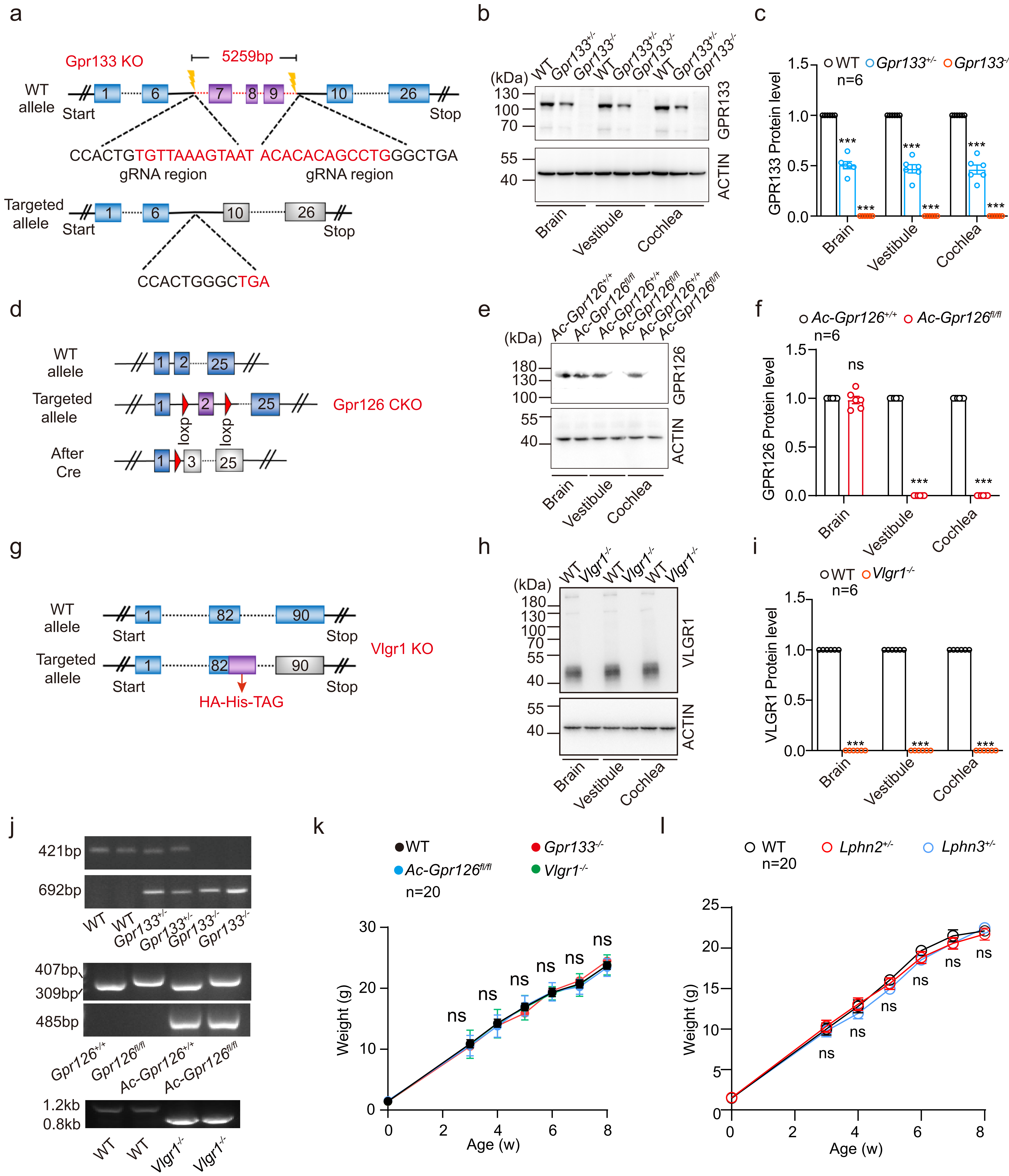

Figure S2. Generation and genotyping of *Gpr133*, *Gpr126* or *Vlgr1* gene knockout mice

(a) Schematic representation of the generation of *Gpr133*$^{-/-}$ mice by CRISPR-Cas9 strategy. A deletion of 5259 bp was introduced between the exons 6 and 10 of the *Gpr133* gene, resulting in a premature translation termination.

(b, c) Western blotting (**b**) and quantitative analysis (**c**) of endogenous expression of GPR133 in the membrane fractions of brain, vestibule and cochlea isolated from the WT or *Gpr133*-deficient mice (n = 6). Data are normalized to the expression levels of GPR133 in respective organs of WT mice. Data are shown as mean ± SEM.***P < 0.001; ns, no significant difference. *Gpr133* knockout mice compared with WT mice. Data were statistically analyzed using one-way ANOVA with Dunnett's post hoc test.

(d) Schematic representation of the generation of *Atoh1-cre*$^{+/-}$*Gpr126*$^{fl/fl}$ mice (referred to as *Ac-Gpr126*$^{fl/fl}$) by Cre–LoxP recombination system. The exon2 of the *Gpr126* gene is deleted upon Atoh1-Cre-mediated recombination, resulting in a premature translation termination and conditional ablation of GPR126 expression in the vestibular or cochlear hair cells.

(e, f) Western blotting (**e**) and quantitative analysis (**f**) of endogenous expression of GPR126 in the membrane fractions of brain, vestibule and cochlea isolated from WT or *Gpr126*-deficient mice (n = 6). Data are normalized to the expression levels of GPR126 in respective organs of WT mice. Data are shown as mean ± SEM.***P < 0.001; ns, no significant difference. *Ac-Gpr126*$^{fl/fl}$ mice compared with *Ac-Gpr126*$^{+/+}$ mice. Data were statistically analyzed using unpaired two-sided Student's *t* test.

(g) Schematic representation of the generation of *Vlgr1*$^{-/-}$ mice by CRISPR-Cas9 strategy. A HA-tag followed by 6×His sequence and a TAG stop codon was introduced after the exon 82 of *Vlgr1* gene to induce a premature translation termination and the deletion of the 7TM and cytoplasmic domains[8].

(h, i) Western blotting (**h**) and quantitative analysis (**i**) of endogenous expression of VLGR1 in the membrane fractions of brain, vestibule and cochlea isolated from WT or *Vlgr1*-deficient mice. Data are normalized to the expression levels of VLGR1 in respective organs of WT mice and the expression of ACTIN in equal amounts of cytosol fractions were used as control (n = 6). Data are shown as mean ± SEM.***P < 0.001. *Vlgr1*$^{-/-}$ mice compared with WT mice. Data were statistically analyzed using unpaired two-sided Student's *t* test.

(j) Genotyping PCR results showing amplified fragments derived from *Gpr133-, Gpr126*-or *Vlgr1*-deficient mice and their wild type littermates.

(k) Body weight changes of *Gpr133*-, *Vlgr1*- or *Gpr126*-deficient mice and their wild type littermates within 8 weeks after birth. (n = 20 mice per group). Data are shown as mean ± SEM. ns, no significant difference. Gene knockout mice compared with WT mice. Data were statistically analyzed using one-way ANOVA with Dunnett's post hoc test.

(l) Body weight changes of WT, *Lphn2*$^{+/-}$, and *Lphn3*$^{+/-}$ mice within 8 weeks after birth (n = 20 mice per group). Data are shown as mean ± SEM. ns, no significant difference. Gene knockout mice compared with WT mice. Data were statistically analyzed using one-way ANOVA with Dunnett's post hoc test.
