# Supplementary material for: A force-sensitive adhesion GPCR is required for equilibrioception
Source: Cell Res. 2025 Feb 18;35(4):243–64. doi: 10.1038/s41422-025-01075-x (PMC11958651; doi:10.1038/s41422-025-01075-x)
Supplement: Supplementary file 3 — Supplementary Figure3 [file 41422_2025_1075_MOESM3_ESM.pdf]

Supplementary information, Figure S3

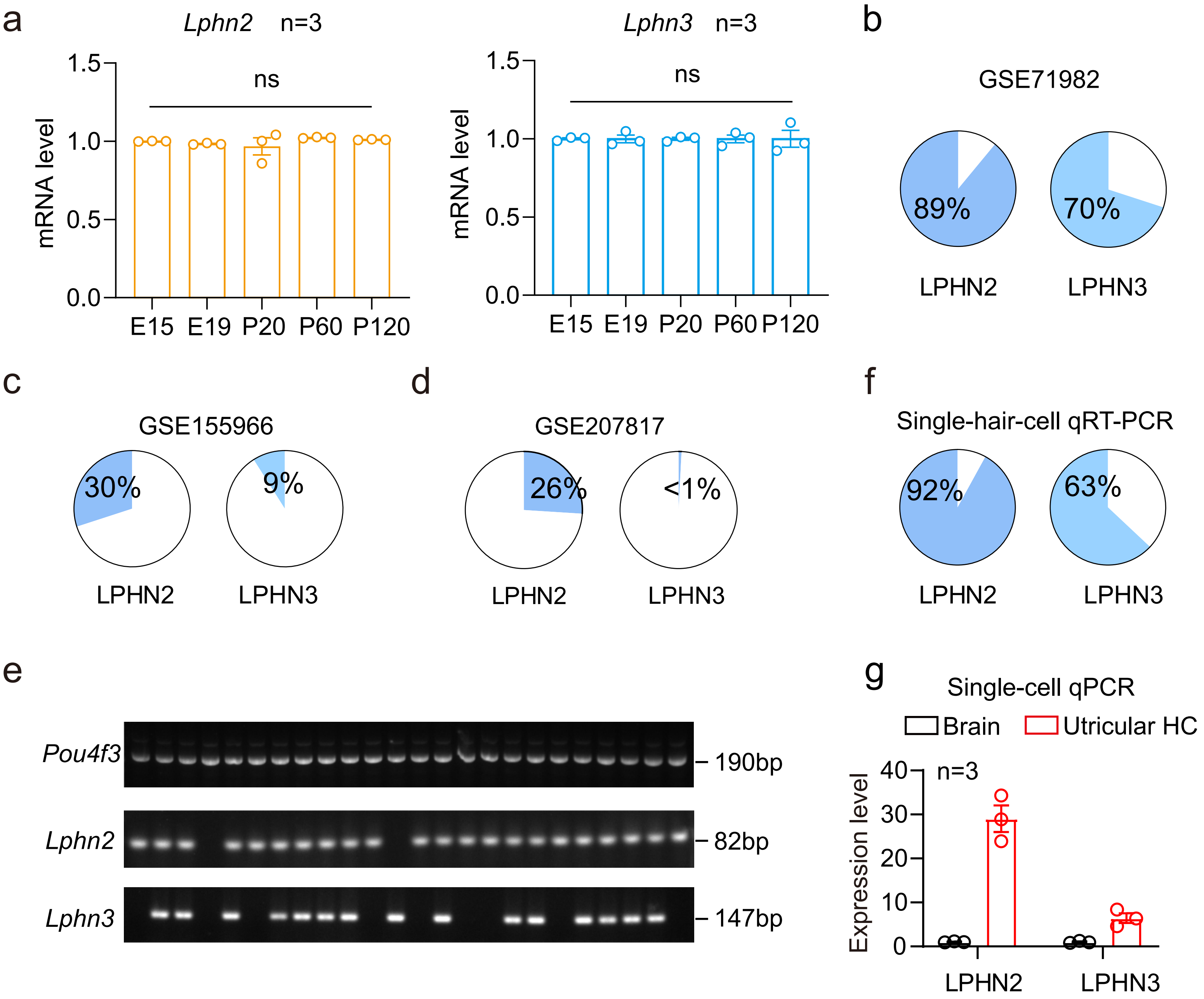

Figure S3. Expression of LPHN2 and LPHN3 in utricular hair cells.

(a) mRNA levels of *Lphn2* (left) and *Lphn3* (right) in the WT mouse utricle harvested at different time points (E15, E19, P20, P60 and P120). Data are normalized to the mRNA levels of their respectively expression at E15 (n = 3). Data are shown as mean ± SEM. ns, no significant difference. mRNA levels of LPHN2 or LPHN3 at different time points compared with that at E15. Data were statistically analyzed using one-way ANOVA with Dunnett's post hoc test.

(b-d) Expression frequency of *Lphn2* and *Lphn3* in mouse or human utricular hair cells. Single cell RNA-seq datasets of utricular hair cells are from GSE71982 (**b**, mouse), GSE155966 (**c**, mouse) and GSE207817 **(d**, human**)**.

(e) Expression profiles of *Lphn2* and *Lphn3* in 24 isolated *Pou4f3*-positive utricular hair cells examined by single-cell qRT-PCR.

(f) Expression frequency of *Lphn2* and *Lphn3* in *Pou4f3*-positive utricular hair cells revealed by single-hair-cell qRT-PCR. Data are correlated to Fig. S3e.

(g) The mRNA levels of *Lphn2* and *Lphn3* in mouse utricular hair cells measured by single-cell qRT–PCR, using their respective expression levels in the brain as a reference (normalized to 1) (n = 3).
