# Supplementary material for: A force-sensitive adhesion GPCR is required for equilibrioception
Source: Cell Res. 2025 Feb 18;35(4):243–64. doi: 10.1038/s41422-025-01075-x (PMC11958651; doi:10.1038/s41422-025-01075-x)
Supplement: Supplementary file 4 — Supplementary Figure4 [file 41422_2025_1075_MOESM4_ESM.pdf]

Supplementary information, Figure S4

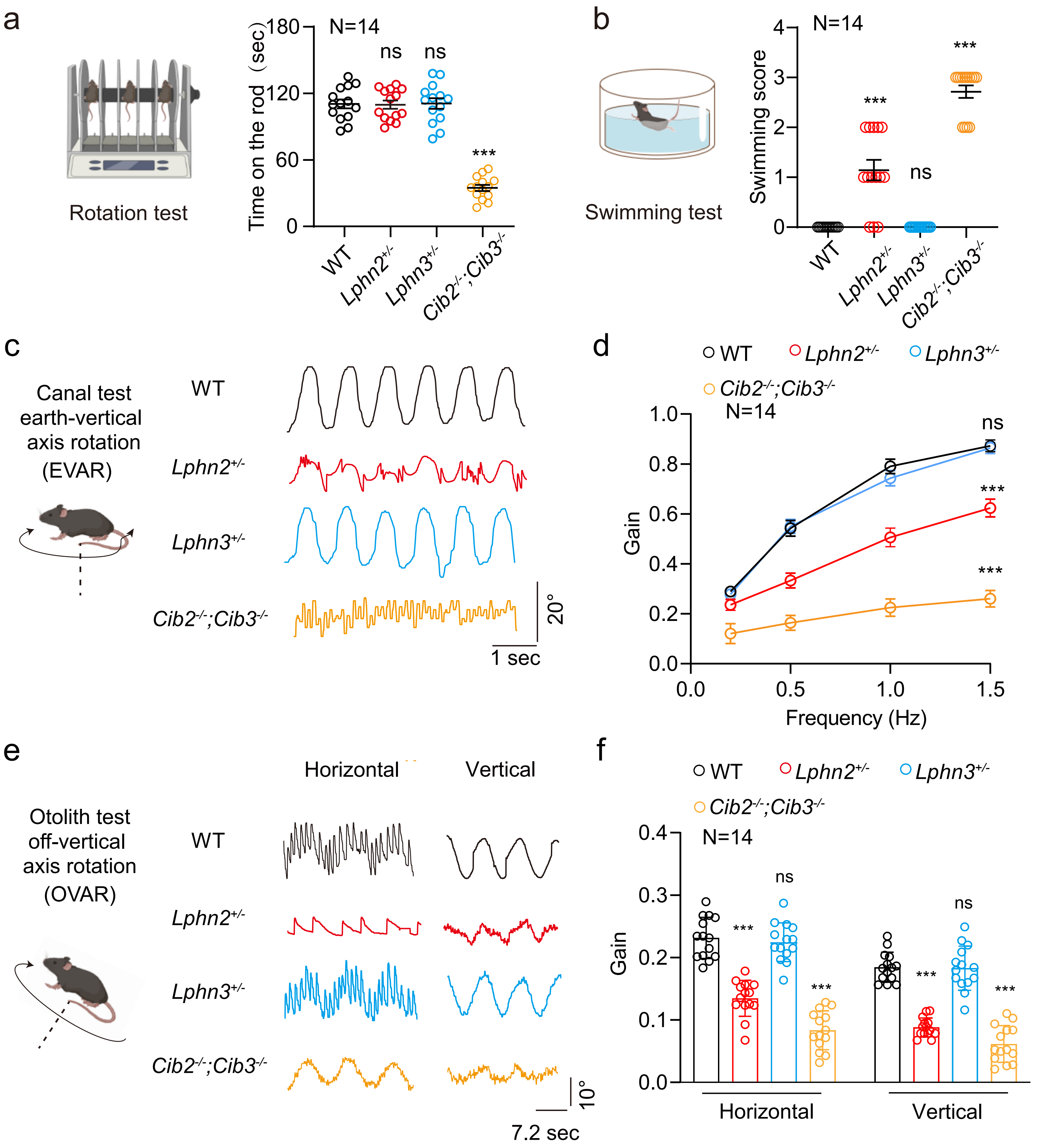

Figure S4. Heterozygous *Lphn2*-deficient mice showed balance disorders

(a) Schematic view (left) and quantification (right) of time on the rotating rod of WT, *Lphn2*$^{+/-}$, *Lphn3*$^{+/-}$ and *Cib2*$^{-/-}$; *Cib3*$^{-/-}$ mice (N = 14 mice per group). Data are shown as mean ± SEM. ***P < 0.001; ns, no significant difference. Gene knockout mice compared with WT mice. Data were statistically analyzed using one-way ANOVA with Dunnett's post hoc test.

(b) Schematic view (left) and quantification (right) of swimming scores of WT, *Lphn2*$^{+/-}$, *Lphn3*$^{+/-}$ and *Cib2*$^{-/-}$; *Cib3*$^{-/-}$ mice in forced swimming test (N = 14 mice per group). Data are shown as mean ± SEM.***P < 0.001; ns, no significant difference. Gene knockout mice compared with WT mice. Data were statistically analyzed using one-way ANOVA with Dunnett's post hoc test.

(c) Schematic view (left) and representative recording curves (right) of the VOR responses of WT, *Lphn2*$^{+/-}$, *Lphn3*$^{+/-}$ and *Cib2*$^{-/-}$; *Cib3*$^{-/-}$ mice to earth-vertical axis rotation (0.25-1.5 Hz, 40°/s peak velocity sinusoidal, whole-body passive rotation).

(d) Quantification of the VOR gain response of WT, *Lphn2*$^{+/-}$, *Lphn3*$^{+/-}$ and *Cib2*$^{-/-}$; *Cib3*$^{-/-}$ mice to earth-vertical axis rotation (N = 14 mice per group). Data are shown as mean ± SEM.***P < 0.001; ns, no significant difference. Gene knockout mice compared with WT mice. Data were statistically analyzed using two-way ANOVA with Dunnett's post hoc test.

(e) Schematic view (left) and representative recording curves (right) of the VOR responses of WT, *Lphn2*$^{+/-}$, *Lphn3*$^{+/-}$ and *Cib2*$^{-/-}$; *Cib3*$^{-/-}$ mice to off-vertical axis rotation (50°/s, whole-body passive rotation).

(f) Quantification of the VOR gain responses of WT, *Lphn2*$^{+/-}$, *Lphn3*$^{+/-}$ and *Cib2*$^{-/-}$; *Cib3*$^{-/-}$ mice to off-vertical axis rotation (N = 14 mice per group). Data are shown as mean ± SEM.***P < 0.001; ns, no significant difference. Gene knockout mice compared with WT mice. Data were statistically analyzed using one-way ANOVA with Dunnett's post hoc test.
