# Supplementary material for: A force-sensitive adhesion GPCR is required for equilibrioception
Source: Cell Res. 2025 Feb 18;35(4):243–64. doi: 10.1038/s41422-025-01075-x (PMC11958651; doi:10.1038/s41422-025-01075-x)
Supplement: Supplementary file 5 — Supplementary Figure5 [file 41422_2025_1075_MOESM5_ESM.pdf]

Supplementary information, Figure S5

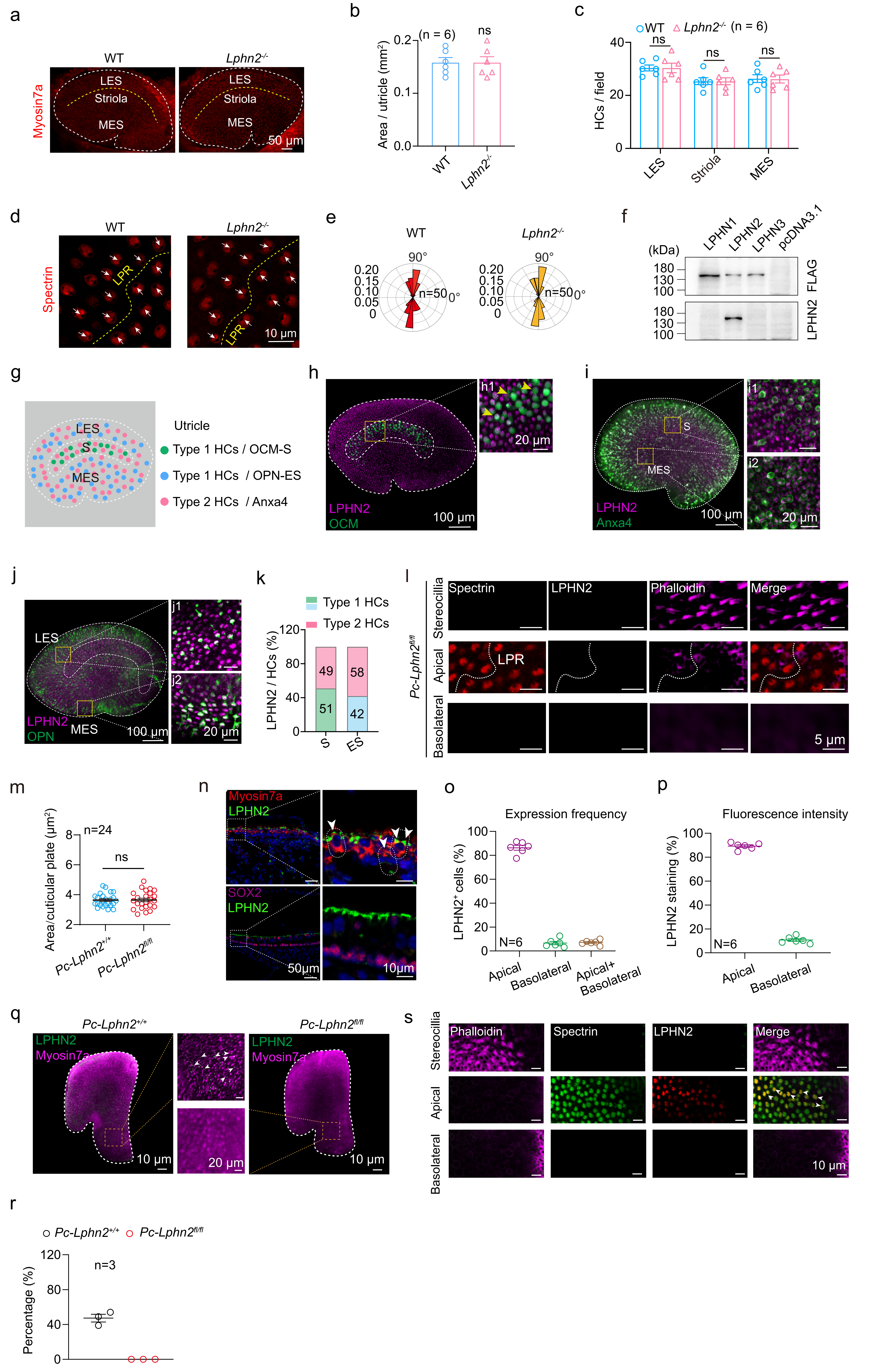

Figure S5. Morphological features of *Lphn2*-deficient utricle in embryonic period and the expression pattern of LPHN2 in saccular hair cells.

(a) Immunostaining of myosin7a (red) in utricular hair cells derived from WT and *Lphn2*$^{-/-}$ mice at E15 (n = 6). Scale bar, 50 µm. LES, lateral extrastriolar region; S, striolar region; MES, medial extrastriolar region.

(b-c) Quantification of the size of utricle (**b**) and the hair cell density at different regions of utricle (**c**) derived from WT and *Lphn2*$^{-/-}$ mice at E15 (n = 6). Field, 50 × 50 µm. Data are correlated to Fig. S5a. Data are shown as mean ± SEM. ns, no significant difference. *Lphn2*$^{-/-}$ mice compared with WT mice. Data were statistically analyzed using unpaired two-sided Student's *t* test.

(d) Immunostaining of βII-spectrin reveals the orientation of hair cells at LPR region of utricles derived from WT and *Lphn2*$^{-/-}$ mice at E15. The hair cell orientation is indicated by the position of the off-center fonticulus without signal. Scale bars: 10 µm.

(e) Circular histogram showing the frequency distribution of hair cell orientation at LPR region of utricles derived from WT and *Lphn2*$^{-/-}$ mice at E15 (n=50 hair cells from 3 mice per group). The lateral side of utricle is defined as 90° (top direction in the histogram).

(f) Immunoblotting of N-terminal Flag-tagged LPHN subfamily members overexpressed in HEK293 cells by anti-Flag antibody or by anti-LPHN2 antibody. Representative blots from three independent experiments were shown (n=3).

(g) Schematic view of hair cell subtypes at different regions of the mouse utricle. OCM (green), Oncomodulin, type I hair cell marker in the S region; SPP1/OPN (blue), osteopontin, type I hair cell marker in the ES region; Anxa4 (pink), Annexin A4, type II hair cell marker.

(h-j) Co-immunostaining of LPHN2 (magenta) with OCM (h, green), Anxa4 (i, green), or OPN (j, green) in mouse utricle whole mounts (n = 3). Scale bar, 100 µm and 20 µm for low- and high-magnification views, respectively.

(k) Quantitative analysis of the expression frequency of LPHN2 in different hair cell subtypes (n = 3). Data are presented as the percentage of type I or II hair cells in all the LPHN2-expressing hair cells in the S or ES region. Data are correlated to Fig. S5h-j.

(l) Co-immunostaining of spectrin (red), LPHN2 (green) and phalloidin (magenta) at different optical planes of hair cells in utricle whole mounts derived from *Pou4f3-CreER*$^{+/-}$*Lphn2*$^{fl/fl}$ mice

(referred to as *Pc-Lphn2*$^{fl/fl}$). Data are correlated to Fig. 2d. Scale bar, 5 µm.

(m) Quantification of the size of cuticular plate (using spectrin as the marker) in utricle whole mounts derived from *Pc-Lphn2*$^{fl/fl}$ mice or *Pc-Lphn2*$^{+/+}$ mice. Data are correlated to Fig. 2d (n = 24 hair cells from 3 mice per group). Data are shown as mean ± SEM. ns, no significant difference. *Pc-Lphn2*$^{fl/fl}$ mice compared with *Pc-Lphn2*$^{+/+}$ mice. Data were statistically analyzed using unpaired two-sided Student's *t* test.

(n) Coimmunostaining of LPHN2 (green) with Myosin7a (red) or Sox2 (magenta) in utricle sections derived from WT mice at P40 (n = 3). Arrows indicate the distribution of LPHN2 immunofluorescence puncta at the apical surface of utricular hair cells (depicted by white dotted lines). Scale bars, 50 µm and 10 µm for low- and high-magnification views, respectively.

(o) Quantification of expression frequency of LPHN2 in different subcellular regions of utricular hair cells (N=6 mice per group; 10 sections were selected from each mouse).

(p) Quantification of fluorescence intensity of LPHN2 in the apical surface (89.7%) and basolateral membrane (10.3%) of utricular hair cells (N=6 mice per group; 10 sections were selected from each mouse).

(q) Representative images of whole-mount coimmunostaining of LPHN2 (green) and myosin7a (magenta) in the saccule of *Pc-Lphn2*$^{+/+}$ mice or *Pc-Lphn2*$^{fl/fl}$ mice at P40 (n = 3). Arrows indicate LPHN2 staining in myosin7a-expressing hair cells. Scale bars, 10 µm and 20 µm for low- and high-magnification views, respectively.

(r) Quantitative analysis of LPHN2 expression in myosin7a-positive saccular hair cells from *Pc-Lphn2*$^{+/+}$ mice or *Pc-Lphn2*$^{fl/fl}$ mice. Data are correlated to Fig. S5q (n = 3).

(s) Coimmunostaining of spectrin (green), LPHN2 (red) and phalloidin (magenta) at different optical planes of hair cells in saccular whole mounts of P40 mice. Arrows indicate coimmunostaining of LPHN2 with spectrin. Scale bar, 10 µm.
