# Supplementary material for: A force-sensitive adhesion GPCR is required for equilibrioception
Source: Cell Res. 2025 Feb 18;35(4):243–64. doi: 10.1038/s41422-025-01075-x (PMC11958651; doi:10.1038/s41422-025-01075-x)
Supplement: Supplementary file 6 — Supplementary Figure6 [file 41422_2025_1075_MOESM6_ESM.pdf]

Supplementary information, Figure S6

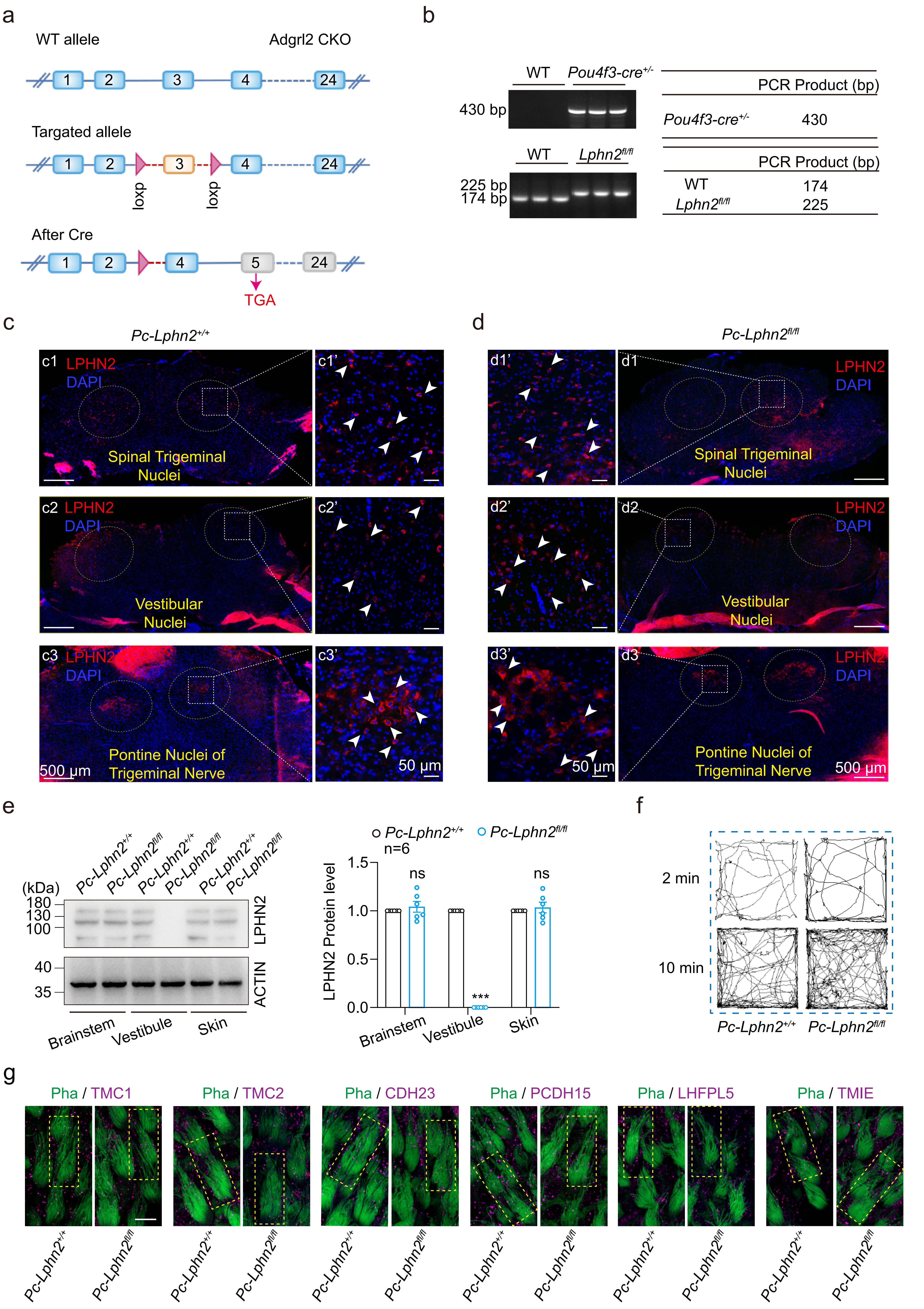

h

	Target protein (the numbers of puncta / hair cell / N = 6)					
Gene	TMC1	TMC2	CDH23	PCDH15	LHFPL5	TMIE
Pc-Lphn2+/+	30 ± 1	17 ± 1	18 ± 2	22 ± 1	24 ± 2	20 ± 2
*Pc-Lphn2*fl/fl	29 ± 2 ns	19 ± 1 ns	19 ± 2 ns	21 ± 1 ns	23 ± 1 ns	19 ± 1 ns

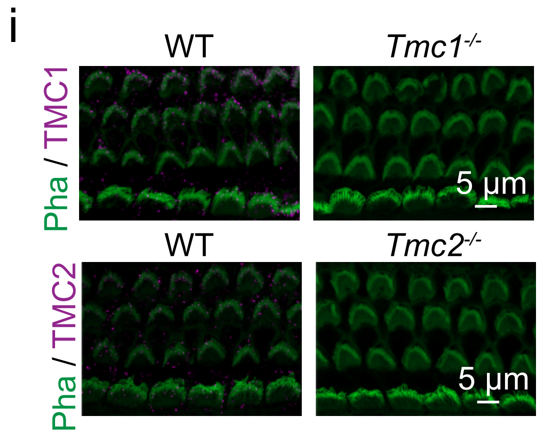

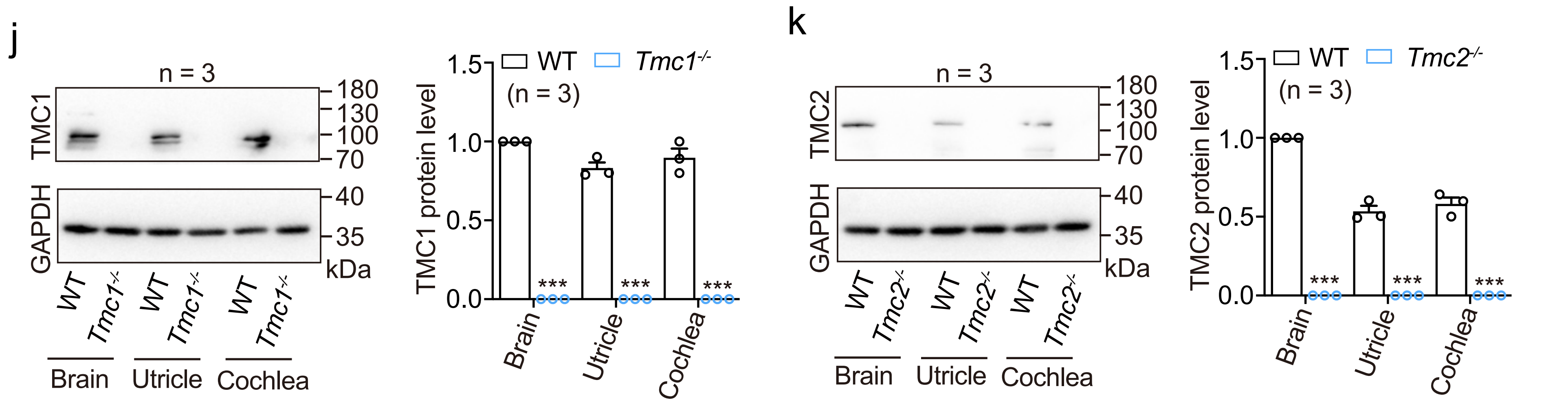

Figure S6. Generation, verification and characterization of hair cell-specific *Lphn2*-deficient mice

(a) Schematic representation of generation of hair cell-specific *Lphn2* knockout mice by crossing the *Lphn2*$^{fl/fl}$ mice with the inducible *Pou4f3-CreER*$^{+/-}$ transgenic mice. The exons 3 premature translation termination of *Lphn2* and the specific ablation of LPHN2 in Pou4f3-expressing hair cells.

(b) Genotyping PCR results showing amplified fragments derived from *Pou4f3-CreER*$^{+/-}$ mice, *Lphn2*$^{fl/fl}$ mice and WT mice.

(c, d) Immunostaining of LPHN2 (red) in the brainstem derived from *Pc-Lphn2*$^{fl/fl}$ mice or *Pc-Lphn2*$^{+/+}$ mice (N = 3 mice per group). Enlarged images show the normal expression of LPHN2 in the spinal trigeminal nuclei (top panel), vestibular nuclei (middle panel) and pontine nuclei of trigeminal nerve (bottom panel) of *Pc-Lphn2*$^{fl/fl}$ mice. Scale bar: 500 μm and 50 μm for low and high magnification view, respectively.

(e) Western blotting (left) and quantitative analysis (right) of endogenous expression of LPHN2 in the brainstem, vestibule and skin isolated from *Pc-Lphn2*$^{fl/fl}$ and *Pc-Lphn2*$^{+/+}$ mice (n=6). Data are normalized to the expression levels of LPHN2 in respective organs of *Pc-Lphn2*$^{+/+}$ mice. Data are shown as mean ± SEM. ***P < 0.001; ns, no significant difference. *Pc-Lphn2*$^{fl/fl}$ mice compared with *Pc-Lphn2*$^{+/+}$ mice. Data were statistically analyzed using unpaired two-sided Student's *t* test.

(f) Representative tracks of *Pc-Lphn2*$^{fl/fl}$ and *Pc-Lphn2*$^{+/+}$ mice in open-field test during 2 min or 10 min tracking period. Data are correlated to Fig. 3e, f.

(g) Zoomed-out images of co-immunostaining of phalloidin (green, referred to as Pha) and different MET machinery components (magenta), including TMC1, TMC2, CDH23, PCDH15, LHFPL5 and TMIE, in utricular hair cells derived from *Pc-Lphn2*$^{+/+}$ and *Pc-Lphn2*$^{fl/fl}$ mice (N = 6 mice per group). Scale bar: 5 μm. Data are correlated to Fig. 4e.

(h) Summary of quantification of immunostaining puncta for each MET machinery component at the stereocilia of utricular hair cell derived from *Pc-Lphn2*$^{+/+}$ and *Pc-Lphn2*$^{fl/fl}$ mice (N = 6 mice per group; 20-30 hair cells were randomly selected from each mouse). Data are correlated to Fig. 4e and Fig. S6g. Data are shown as mean ± SEM. ***P < 0.001; ns, no significant difference. *Pc-Lphn2*$^{fl/fl}$ mice compared with *Pc-Lphn2*$^{+/+}$ mice. Data were statistically

analyzed using unpaired two-sided Student's *t* test.

(i) Co-immunostaining of TMC1 (upper, magenta) or TMC2 (lower, magenta) with Phalloidin (Pha, green) in the stereocilia of hair cells in the cochlear whole mounts derived from WT and *Tmc1*$^{-/-}$ or *Tmc2*$^{-/-}$ mice (N = 6 mice per group). Scale bar, 5 μm.

(j) Representative blotting (left) and quantitative analysis (right) of the protein levels of endogenous TMC1 in cochlea, utricle and brain isolated from the WT and *Tmc1*$^{-/-}$ mice (n = 3). Data are shown as mean ± SEM. ***P < 0.001. *Tmc1*$^{-/-}$ mice compared with WT mice. Data were statistically analyzed using unpaired two-sided Student's *t* test.

(k) Representative blotting (left) and quantitative analysis (right) of the protein levels of endogenous TMC2 in cochlea, utricle and brain isolated from the WT and *Tmc2*$^{-/-}$ mice (n = 3). Data are shown as mean ± SEM. ***P < 0.001. *Tmc2*$^{-/-}$ mice compared with WT mice. Data were statistically analyzed using unpaired two-sided Student's *t* test.
