# Supplementary material for: A force-sensitive adhesion GPCR is required for equilibrioception
Source: Cell Res. 2025 Feb 18;35(4):243–64. doi: 10.1038/s41422-025-01075-x (PMC11958651; doi:10.1038/s41422-025-01075-x)
Supplement: Supplementary file 7 — Supplementary Figure7 [file 41422_2025_1075_MOESM7_ESM.pdf]

# Supplementary information, Figure S7

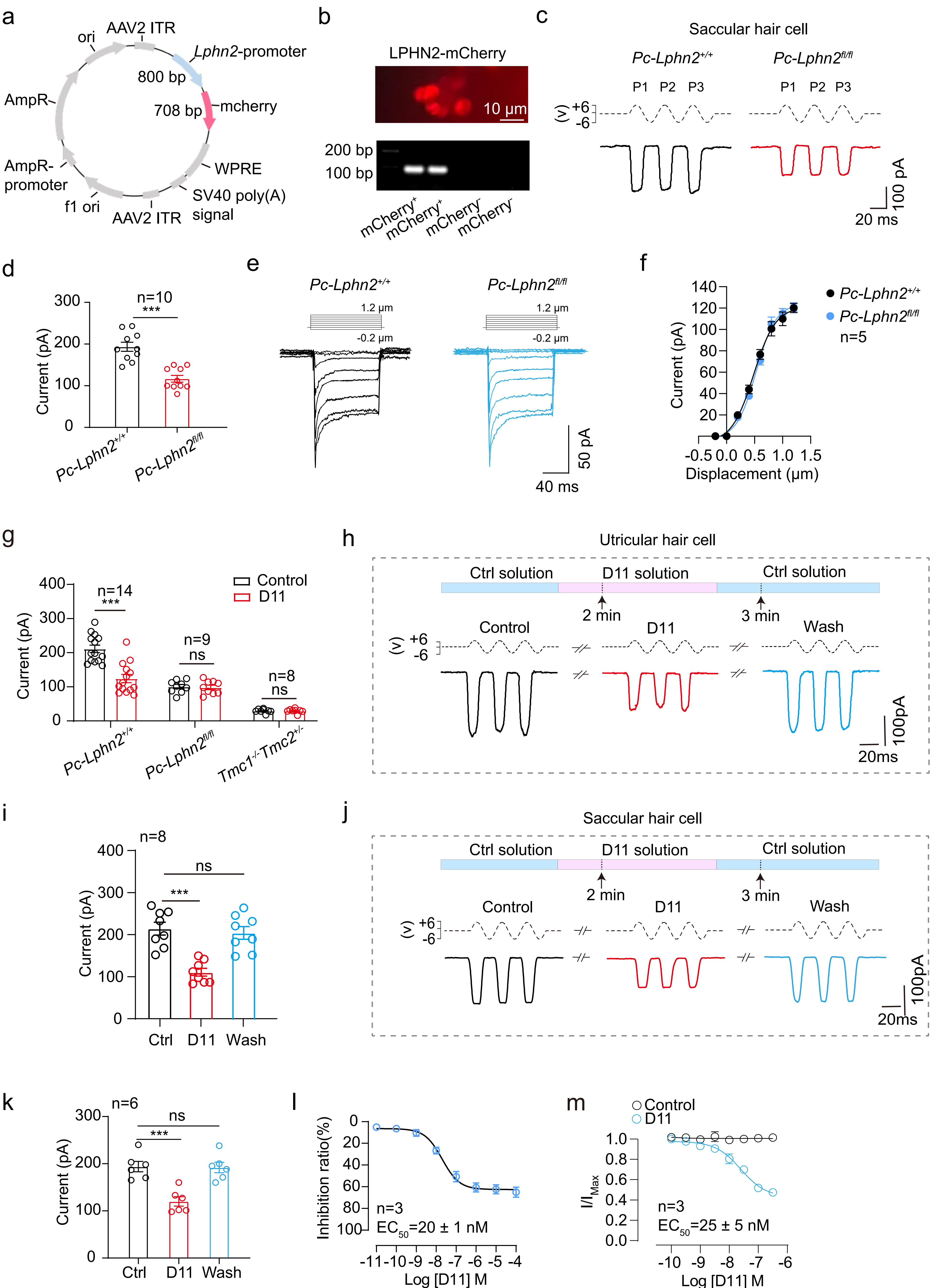

**Figure S7. Genetic ablation or pharmacological inhibition of LPHN2 impairs MET currents in vestibular hair cells**

**(a)** Schematic diagram showing the construction of AAV-ie-*Lphn2pr*-mCherry for the labelling of LPHN2-expressing vestibular hair cells.

**(b)** Representative fluorescent image (top panel) and single-cell RT-PCR results (bottom panel) showing the specific expression of LPHN2 in AAV-ie-*Lphn2pr*-mCherry-labeled utricular hair cells. Scale bar, 10 μm.

**(c, d)** Representative current traces **(c)** and quantitative analysis **(d)** of the MET currents induced by sinusoidal fluid jet stimulation in saccular hair cells of *Pc-Lphn2*$^{+/+}$ mice (black) or *Pc-Lphn2*$^{fl/fl}$ mice (red) at P10 (n = 10). Data are shown as mean ± SEM. ***P < 0.001; *Pc-Lphn2*$^{+/+}$ mice compared with *Pc-Lphn2*$^{fl/fl}$ mice. Data were statistically analyzed using unpaired two-sided Student's *t* test.

**(e)** Representative mechanotransduction currents in utricular hair cells from *Pc-Lphn2*$^{+/+}$ (black traces) or *Pc-Lphn2*$^{fl/fl}$ mice (blue traces) in response to a set of 100 ms hair bundle deflections ranging from −0.2 μm to 1.2 μm (0.2 μm steps).

**(f)** Current displacement plots obtained from similar data as shown in (e) (n = 5).

**(g)** MET responses in utricular hair cells derived from *Pc-Lphn2*$^{+/+}$, *Pc-Lphn2*$^{fl/fl}$ or *Tmc1*$^{-/-}$ *Tmc2*$^{+/-}$ mice in the absence or presence of 50 nM D11 (n = 14, 9 and 8 for *Pc-Lphn2*$^{+/+}$, *Pc-Lphn2*$^{fl/fl}$ and *Tmc1*$^{-/-}$ *Tmc2*$^{+/-}$ cells, respectively). Data are correlated to Fig. 5d, e. Data are shown as mean ± SEM. ***P < 0.001; ns, no significant difference. Utricular hair cells treated with D11 compared with the control cells. Data were statistically analyzed using paired two-sided Student's *t* test.

**(h, i)** Representative current traces (**h**) and quantitative analysis **(i)** of fluid jet-stimulated MET currents in utricular hair cells before and after treatment with 50 nM D11 (n = 8). The MET current returned to normal level after washing out D11. D11 solution was bath applied using a gravity fed application system at a perfusion rate of 2-3 ml/min and reached the recording chamber to replace the control solution. Electrophysiological recording was performed 2 min after perfusion. To wash away the D11, D11 solution was replaced by the control solution and the currents were recorded after continuous perfusion by control solution for 3 min. Data are shown as mean ± SEM. ***P < 0.001; ns, no significant difference. Utricular hair cells treated

with D11 or after washing compared with the control cells. Data were statistically analyzed using paired two-sided Student's *t* test.

**(j, k)** Representative current traces (**j**) and quantitative analysis (**k**) of fluid jet-stimulated MET currents in saccular hair cells before and after treatment with 50 nM D11 (n = 6). The MET current returned to normal level after washing out D11. Data are shown as mean ± SEM. ***P < 0.001; ns, no significant difference. Saccular hair cells treated with D11 or after washing compared with the control cells. Data were statistically analyzed using paired two-sided Student's *t* test.

**(l)** Dose-dependent inhibitory effects of D11 on the force (3 pN)-induced Gs activation in LPHN2-overexpressing HEK293 cells (n = 3).

**(m)** Dose-dependent inhibitory effects of D11 on the fluid jet-stimulated MET currents in utricular hair cells (n=3). Data are normalized to the maximal MET response of control vehicle-treated hair cells in respective groups.
