# Supplementary material for: A force-sensitive adhesion GPCR is required for equilibrioception
Source: Cell Res. 2025 Feb 18;35(4):243–64. doi: 10.1038/s41422-025-01075-x (PMC11958651; doi:10.1038/s41422-025-01075-x)
Supplement: Supplementary file 8 — Supplementary Figure8 [file 41422_2025_1075_MOESM8_ESM.pdf]

Supplementary information, Figure S8

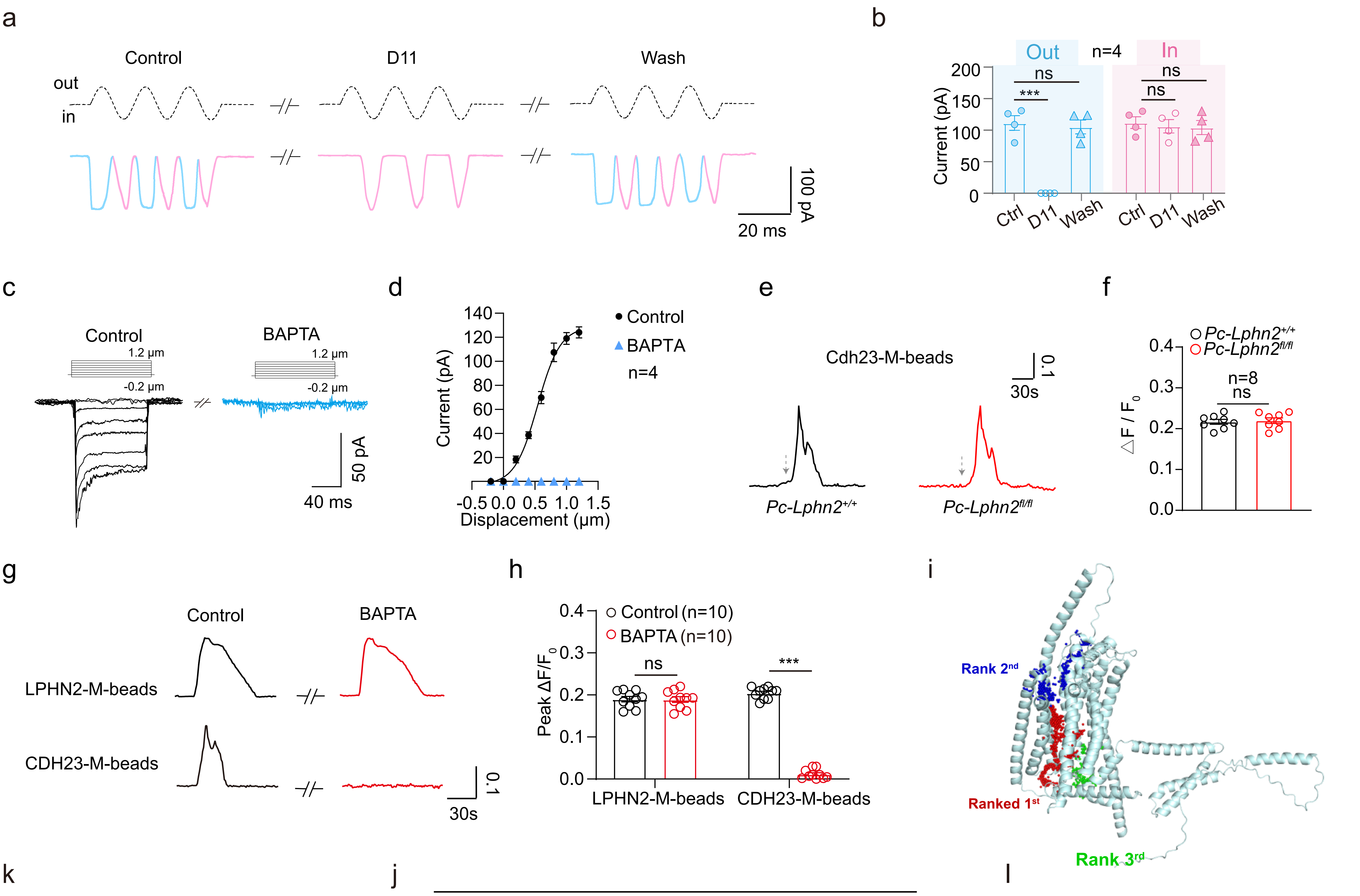

k

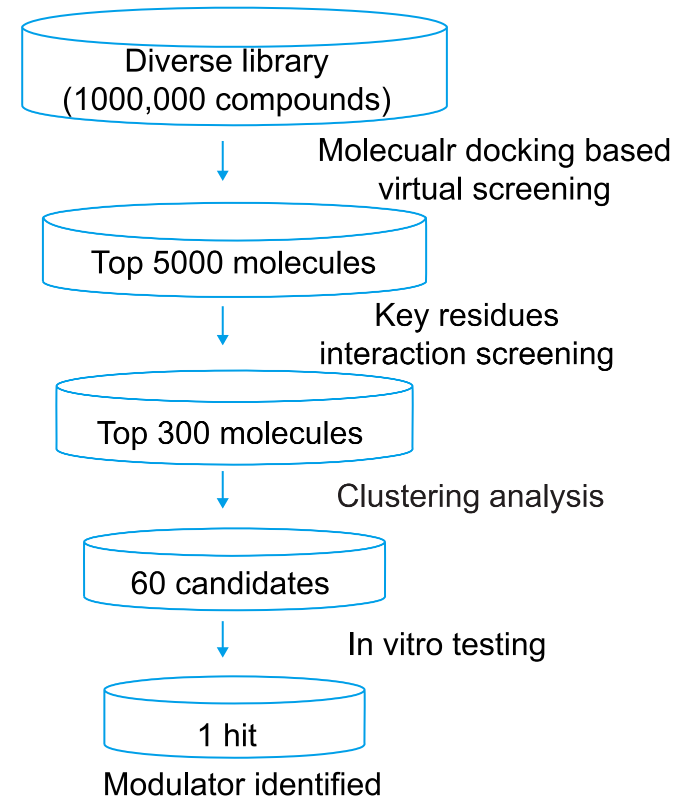

j

Pocket	Site Score	Size	Dscore	Volume(Å³)
1	1.086	362	1.121	1163.456
2	1.016	277	1.032	844.123
3	0.992	185	0.964	561.491

l

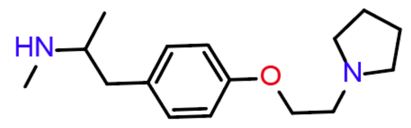

C14

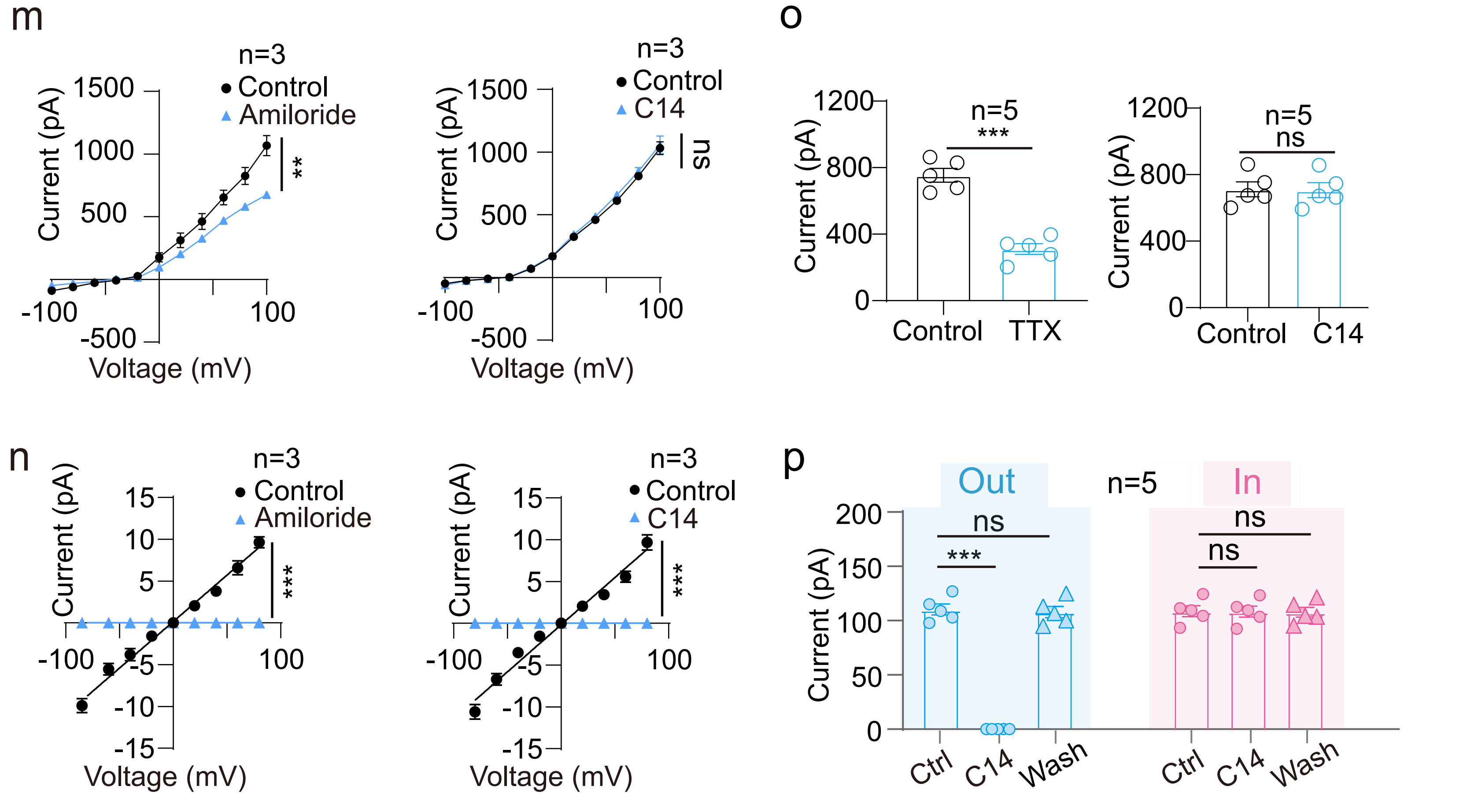

Figure S8. The residual normal-polarity MET current in BAPTA-treated utricular hair cells is LPHN2 and TMC1 dependent

(a, b) Representative current traces (**a**) and quantitative analysis (**b**) of fluid jet-stimulated MET responses in BAPTA-treated utricular hair cells before and after treatment with 50 nM D11 (n = 4). The saturated currents could be recovered after washing out D11. The normal-polarity and reverse-polarity MET current traces are colored blue and pink, respectively. Data are shown as mean ± SEM. ***P < 0.001; ns, no significant difference. Utricular hair cells treated with D11 or after washing compared with those treated with control vehicle. Data were statistically analyzed using paired two-sided Student's *t* test.

(c) Representative mechanotransduction currents in utricular hair cells before and after treatment with BAPTA for 5 min in response to a set of 100 ms hair bundle deflections ranging from -0.2 μm to 1.2 μm (0.2 μm steps).

(d) Current displacement plots obtained from similar data as shown in (c) (n = 4).

(e, f) Representative traces (**e**) and quantitative analysis (**f**) of the Ca^{2+} responses in individual utricular hair cell derived from P10 *Pc-Lphn2*$^{+/+}$ mice or *Pc-Lphn2*$^{fl/fl}$ mice in response to force applied by CDH23-M-beads (n=8). Data are shown as mean ± SEM. ns, no significant difference. *Pc-Lphn2*$^{fl/fl}$ utricular hair cells compared with *Pc-Lphn2*$^{+/+}$ utricular hair cells. Data were statistically analyzed using unpaired two-sided Student's *t* test.

(g, h) Representative traces **(g)** and quantitative analysis **(h)** of Ca^{2+} signal in utricular hair cells in response to force applied by CDH23-M-beads or LPHN2-M-beads before and after treatment with BAPTA for 5 min (n = 10). Data are shown as mean ± SEM. ***P < 0.001; ns, no significant difference. Utricular hair cells treated with BAPTA compared with those treated with control vehicle. Data were statistically analyzed using paired two-sided Student's *t* test.

(i) Predicted ligand-binding pockets in mouse TMC1 determined using the SiteMap algorithm. The mouse TMC1 structure was modelled using AlphaFold2. The predicted binding pockets are filled with dark red, blue or green balls.

(j) Calculated results of the predicted top 3 ligand-binding pockets in modelled mouse TMC1 structure.

(k) Flowchart showing the procedures for in-silico screening of TMC1 inhibitors. 1 million compounds were virtually screened against the putative pocket (Pocket 1) of TMC1 and the top

5000 were selected for the second round of screening against key pocket residues. The resultant top 300 molecules were subjected to clustering analysis, leading to 60 candidates. The final hit was identified by *in vitro* and *in vivo* electrophysiology recording assay.

(l) The chemical structure of the TMC1 inhibitor C14.

(m) Effects of MET channel blocker Amiloride (left) and TMC1 inhibitor C14 (right) on the CNG currents recorded in CNGA3-overexpressed HEK293 cells (n=3). Whole-cell currents were elicited by voltage steps between -100 mV and +100mV, and the corresponding I-V curves were generated. Data are shown as mean ± SEM. **P < 0.01; ns, no significant difference. Utricular hair cells treated with Amiloride or C14 compared with the control cells. Data were statistically analyzed using paired two-sided Student's *t* test.

(n) Effects of MET channel blocker Amiloride (left) and TMC1 inhibitor C14 (right) on spontaneous single-channel currents recorded in HEK293 cells transfected with TMC1 and LGR6 (n=3). Spontaneous single-channel currents were elicited by voltage steps between -80 mV and +80 mV, and the corresponding I-V curves were generated. Data are shown as mean ± SEM. ***P < 0.001. Utricular hair cells treated with Amiloride or C14 compared with the control cells. Data were statistically analyzed using paired two-sided Student's *t* test.

(o) Effects of sodium channel blocker TTX (left) and TMC1 inhibitor C14 (right) on sodium currents measured at -120 mV in primary utricular hair cells (n=5). Data are shown as mean ± SEM. ***P < 0.001; ns, no significant difference. Utricular hair cells treated with TTX or C14 compared with the control cells. Data were statistically analyzed using paired two-sided Student's *t* test.

(p) Quantitative analysis of fluid jet-stimulated MET responses in BAPTA-treated utricular hair cells before and after treatment with 1 μM C14 (n = 5). The residual normal-polarity MET currents could be recovered after washing out C14. Data are shown as mean ± SEM. ***P < 0.001; ns, no significant difference. Utricular hair cells treated with C14 or after washing compared with those treated with control vehicle. Data were statistically analyzed using paired two-sided Student's *t* test.
