# Supplementary material for: A force-sensitive adhesion GPCR is required for equilibrioception
Source: Cell Res. 2025 Feb 18;35(4):243–64. doi: 10.1038/s41422-025-01075-x (PMC11958651; doi:10.1038/s41422-025-01075-x)
Supplement: Supplementary file 9 — Supplementary Figure9 [file 41422_2025_1075_MOESM9_ESM.pdf]

Supplementary information, Figure S9

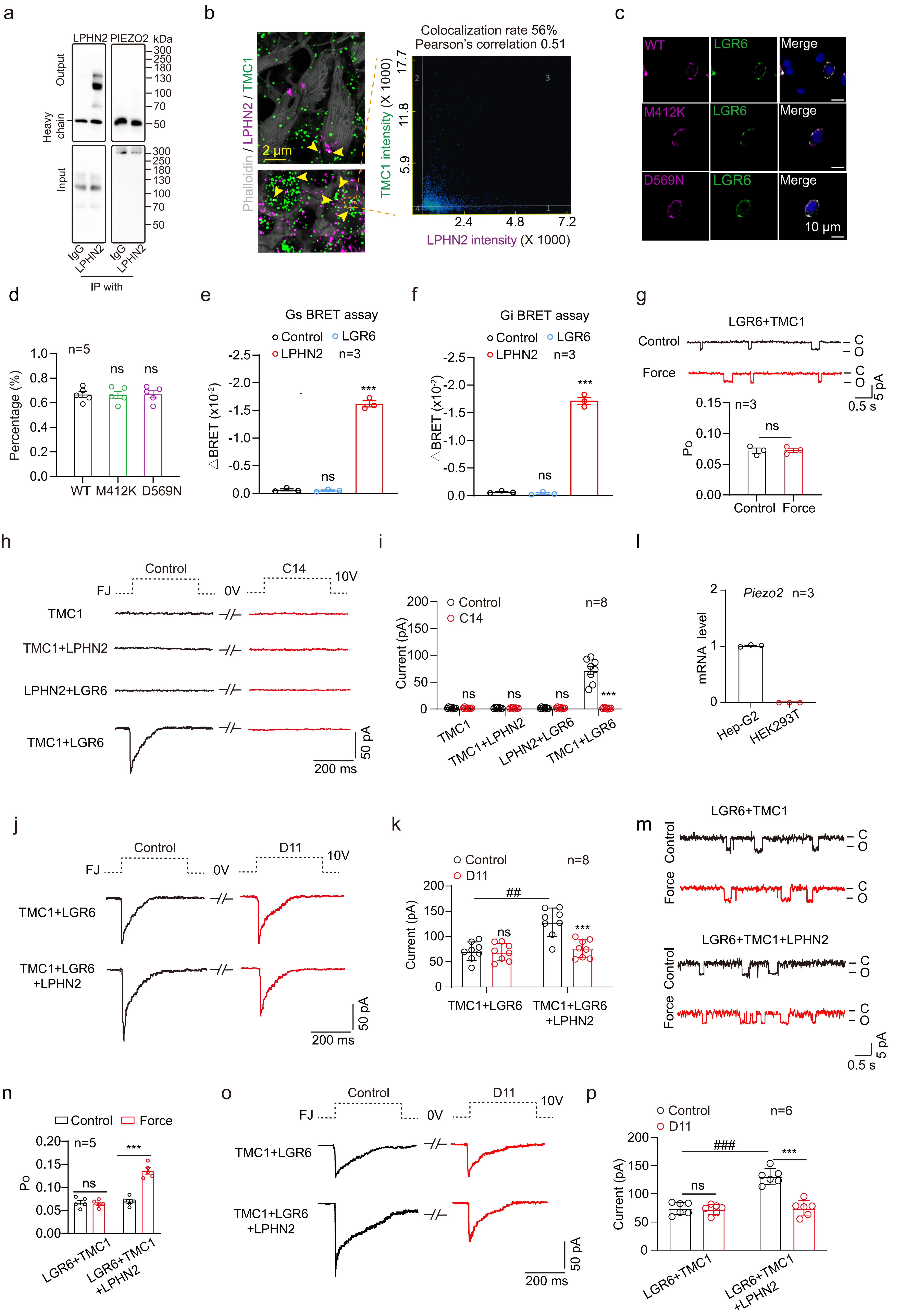

Figure S9. Force sensation by LPHN2 but not LGR6 regulates TMC1 opening in the heterologous system

(a) Co-immunoprecipitation of LPHN2 with Piezo2 in the lysates of mouse utricles. Representative blots from three independent experiments are shown (n = 3).

(b) Co-immunostaining of LPHN2 (magenta) with TMC1 (green) in the apical surface of utricular hair cells in utricle whole mounts derived from WT mice (N = 6 mice per group). Scale bar: 2 μm. Arrows indicate the colocalization of LPHN2 with TMC1 at the apical surface of utricular hair cells. The Pearson's correlation analysis of the fluorescence intensities of LPHN2 and TMC1 revealed a correlation coefficient of 0.51.

(c) Co-immunostaining of LGR6 (green) with TMC1 (WT or mutants, magenta) in HEK293 cells. Scale bar: 10 μm. Representative images from five independent experiments are shown (n = 5).

(d) Quantitative analysis of the cell-surface expression efficiency of WT TMC1 or TMC1 mutants in HEK293 cells. Data are presented as the percentage of TMC1 immunofluorescence intensity on plasma membrane. The cells exhibiting more than 50% of TMC1 expression on plasma membrane (approximately 20% of the transfected cells) were selected for analysis and the following electrophysiological recording. Data are from 5 independent experiments (n=5). Data are shown as mean ± SEM. ns, no significant difference. TMC1 mutants compared with WT TMC1. Data were statistically analyzed using one-way ANOVA with Dunnett's post hoc test.

(e-f) Gs (**e**) or Gi3 (**f**) activation in HEK293 cells overexpressing LGR6 or LPHN2 in response to 10 pN force stimulation measured by G protein dissociation BRET assay (n = 3). HEK293 cells transfected with empty vector pcDNA3.1 and G protein probes were used as the negative control. Data are shown as mean ± SEM. ***P < 0.001; ns, no significant difference. HEK293 cells transfected with LPHN2 or LGR6 compared with those transfected with pcDNA3.1. Data were statistically analyzed using one-way ANOVA with Dunnett's post hoc test.

(g) Representative traces (upper) and quantitative analysis (bottom) of the single-channel currents recorded in HEK293 cells transfected with TMC1 and LGR6 under control condition (black) or in response to 10 pN force stimulation (red) (n = 3). Data are shown as mean ± SEM. ns, no significant difference. Force-stimulated cells compared with control cells. Data were

statistically analyzed using paired two-sided Student's *t* test.

(h-i) Representative traces **(h)** and quantitative analysis **(i)** of the whole-cell currents recorded in HEK293 cells transfected with TMC1 alone, TMC1/LPHN2, LPHN2/LGR6 or TMC1/LGR6 in the absence (black) or presence (red) of 1 μM C14 (n = 8). A one-time step fluid jet (10 V and 400 ms square-wave stimulation) was used to evoke the currents. Data are shown as mean ± SEM. ***P < 0.001; ns, no significant difference. C14-treated HEK293 cells compared with control cells. Data were statistically analyzed using paired two-sided Student's *t* test.

(j-k) Representative traces **(j)** and quantitative analysis **(k)** of the whole-cell currents recorded in HEK293 cells transfected with TMC1/LGR6 or TMC1/LGR6/LPHN2 in the absence (black) or presence (red) of 50 nM D11 (n = 8). Data are shown as mean ± SEM. ***P < 0.001; ns, no significant difference. D11-treated HEK293 cells compared with control cells; $^{\#\#}$P < 0.01. HEK293 cells transfected with TMC1/LGR6/LPHN2 compared with those transfected with TMC1/LGR6. Data were statistically analyzed using two-way ANOVA with Dunnett's post hoc test.

(l) mRNA levels of *Piezo2* in HepG2 cells or HEK293 cells measured by qRT-PCR. HepG2 cell line was used as positive control. Data are normalized to the mRNA levels of *Piezo2* in HepG2 cells (n = 3).

(m) Representative spontaneous and force (10 pN)-stimulated single-channel current traces recorded in *Piezo1* knockout HEK293T (P1-KO-HEK) cells transfected with TMC1/LGR6 or TMC1/LGR6/LPHN2.The force was applied on LPHN2 through LPHN2-M-beads.

(n) Quantitative analysis of the single-channel open probability in P1-KO-HEK cells transfected with TMC1/LGR6 or TMC1/LGR6/LPHN2 (n = 5 per group). Data are shown as mean ± SEM. ***P < 0.001; ns, no significant difference. Force-stimulated P1-KO-HEK cells compared with the control cells. Data were statistically analyzed using paired two-sided Student's *t* test.

(o, p) Representative traces **(o)** and quantitative analysis **(p)** of the whole-cell currents recorded in P1-KO-HEK cells transfected with TMC1/LGR6 or TMC1/LGR6/LPHN2 in the absence (black) or presence (red) of 50 nM D11 (n = 6 per group). A one-time step fluid jet (10 V and 400 ms square-wave stimulation) was used to evoke the currents. Data are shown as mean ± SEM.***P < 0.001; ns, no significant difference. P1-KO-HEK cells treated with D11 compared

with the control cells. $^{###}P < 0.001$. P1-KO-HEK cells transfected with TMC1/LGR6/LPHN2 compared with those transfected with TMC1/LGR6. Data were statistically analyzed using two-way ANOVA with Dunnett's post hoc test.
