# Supplementary material for: A force-sensitive adhesion GPCR is required for equilibrioception
Source: Cell Res. 2025 Feb 18;35(4):243–64. doi: 10.1038/s41422-025-01075-x (PMC11958651; doi:10.1038/s41422-025-01075-x)
Supplement: Supplementary file 10 — Supplementary Figure10 [file 41422_2025_1075_MOESM10_ESM.pdf]

Supplementary information, Figure S10

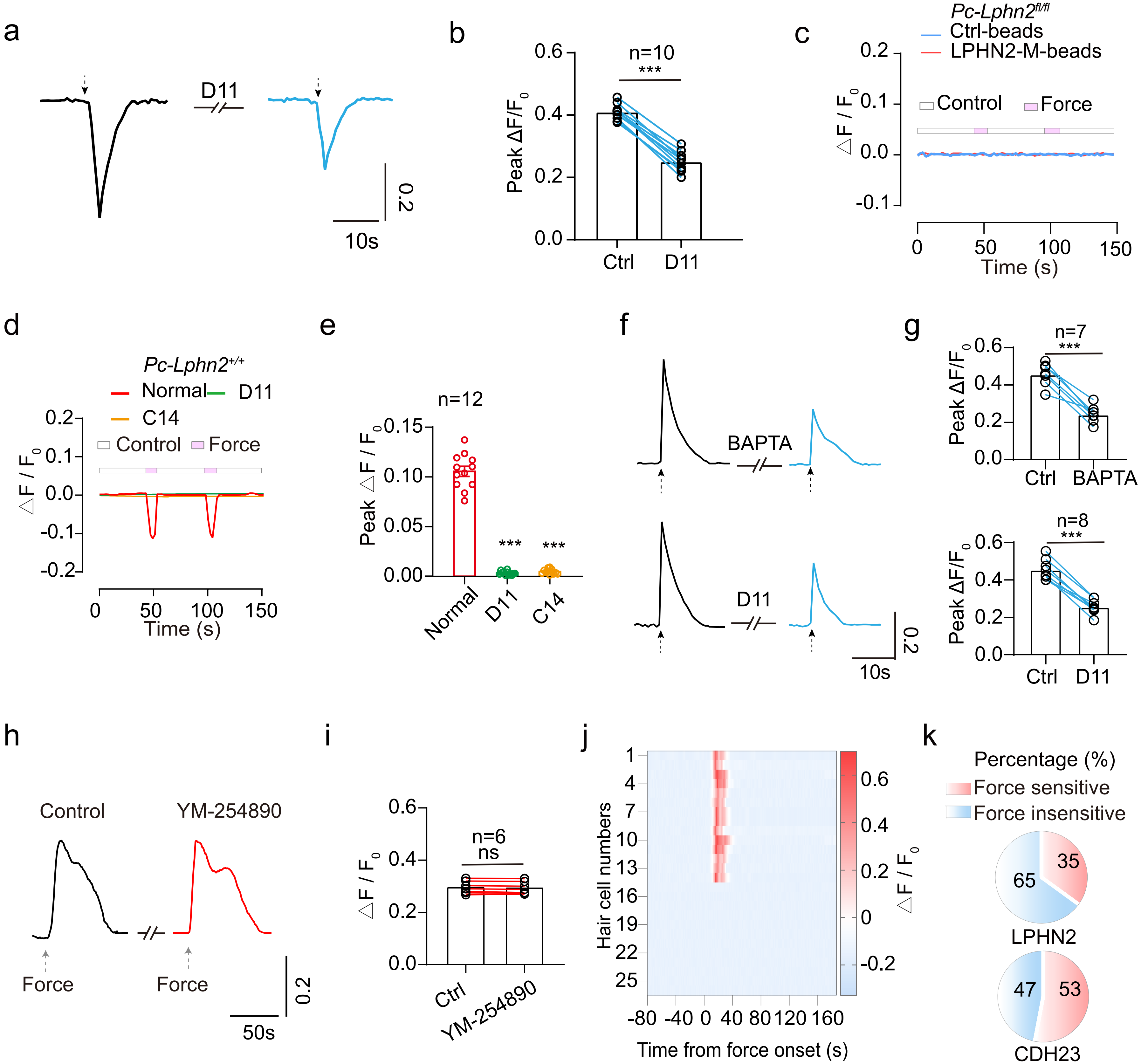

Figure S10. Both tip link and LPHN2 contribute to force-stimulated glutamate release and Ca^{2+} signals in utricular hair cells

(a, b) Representative traces (**a**) and quantitative analysis (**b**) of fluid jet-stimulated glutamate secretion from utricular hair cells in the absence or presence of 50 nM D11 (n = 10). Data are shown as mean ± SEM.***P < 0.001. Utricular hair cells treated with D11 compared with those treated with control vehicle. Data were statistically analyzed using paired two-sided Student's *t* test.

(c) Representative traces of glutamate secretion from individual utricular hair cell derived from *Pc-Lphn2$^{fl/fl}$* mice in response to force applied by LPHN2-M-beads or control beads. Data are correlated to Fig. 8e, f.

(d, e) Representative traces (**d**) and quantitative analysis (**e**) of glutamate secretion from individual utricular hair cell derived from P10 *Pc-Lphn2$^{+/+}$* mice pretreated with control vehicle, 50 nM D11 or 1 μM C14 in response to force applied by LPHN2-M-beads (n = 12). Data are shown as mean ± SEM. ***P < 0.001. Utricular hair cells pretreated with D11 or C14 compared with those treated with control vehicle. Data were statistically analyzed using one-way ANOVA with Dunnett's post hoc test.

(f, g) Representative traces (**f**) and quantitative analysis (**g**) of fluid jet-stimulated Ca^{2+} signals in utricular hair cells in the absence or presence of 5 mM BAPTA or 50 nM D11 (n = 7 hair cells and 8 hair cells for BAPTA and D11 treatment, respectively). Data are shown as mean ± SEM. ***P < 0.001. Utricular hair cells treated with BAPTA or D11 compared with those treated with control vehicle. Data were statistically analyzed using paired two-sided Student's *t* test.

(h, i) Representative traces **(h)** and quantitative analysis **(i)** of the force-induced Ca^{2+} responses in LPHN2-expressing utricular hair cells before or after treatment with 60 μM YM-254890 (n=6). Data are shown as mean ± SEM. ns, no significant difference. YM-254890-treated hair cells compared with control cells. Data were statistically analyzed using paired two-sided Student's *t* test.

(j) Heatmaps showing the Ca^{2+} responses in individual utricular hair cell in response to force applied by CDH23-M-beads (n=26).

(k) Proportion of force-sensitive and force-insensitive utricular hair cells in response to force stimulation applied by CDH23-M-beads or Lphn2-M-beads. Lphn2-M-beads were applied on

mCherry-labelled utricular hair cells while CDH23-M-beads were applied on randomly-selected hair cells. Data are correlated to Fig. 8k and Fig. S10j.
