# Supplementary material for: A force-sensitive adhesion GPCR is required for equilibrioception
Source: Cell Res. 2025 Feb 18;35(4):243–64. doi: 10.1038/s41422-025-01075-x (PMC11958651; doi:10.1038/s41422-025-01075-x)
Supplement: Supplementary file 11 — Supplementary Figure11 [file 41422_2025_1075_MOESM11_ESM.pdf]

Supplementary information, Figure S11

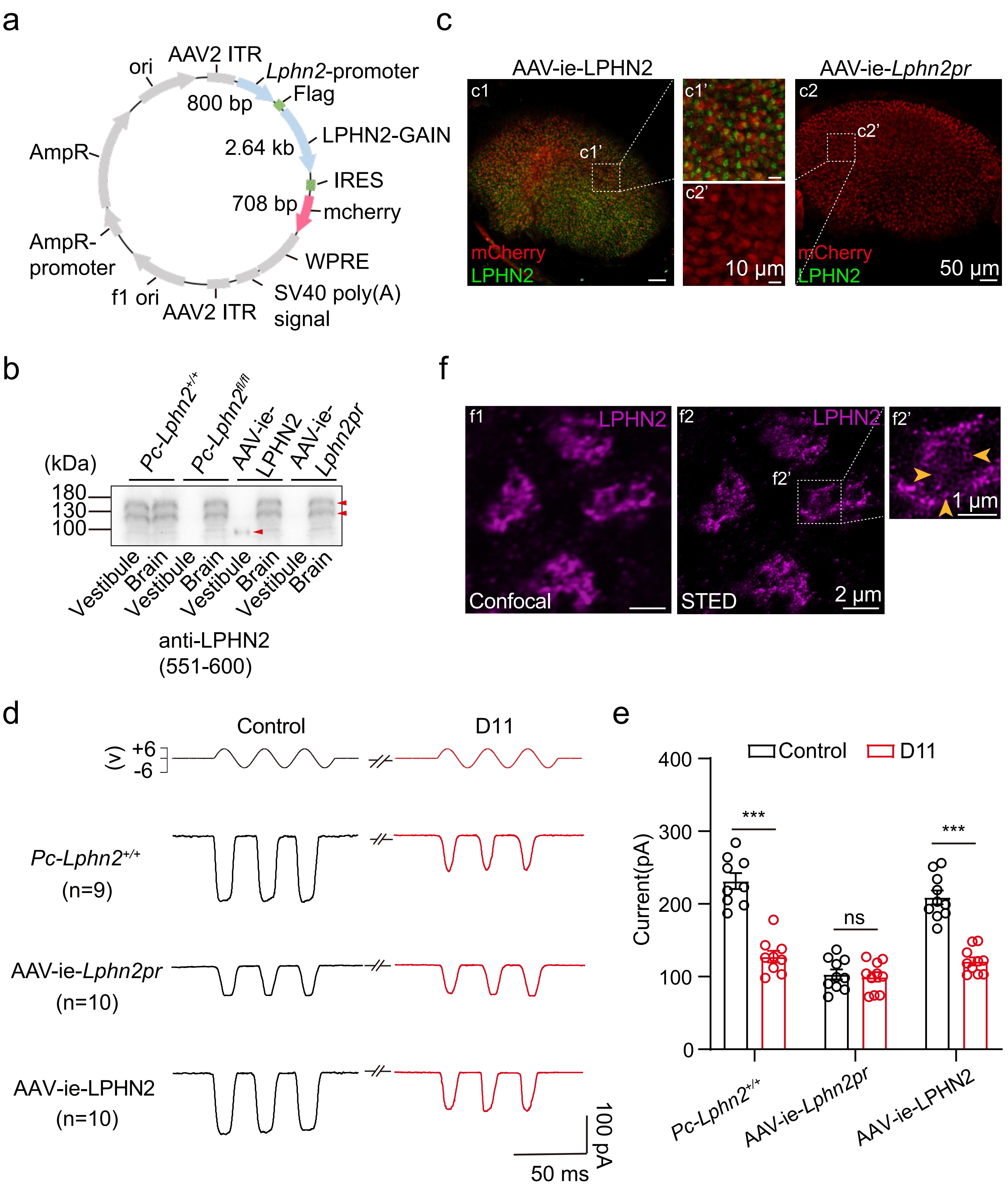

Figure S11. Re-expression of LPHN2 in vestibular hair cells of *Lphn2*-deficient mice through AAV-mediated gene delivery

(a) Schematic diagram showing the construction of AAV-ie-LPHN2-mCherry for in vivo rescue experiment.

(b) Representative western blotting showing the expression of LPHN2 in the membrane fractions of the vestibule or brain isolated from *Pc-Lphn2*$^{+/+}$, *Pc-Lphn2*$^{fl/fl}$, AAV-ie-LPHN2-treated *Pc-Lphn2*$^{fl/fl}$ mice (referred to as AAV-ie-LPHN2 mice) and AAV-ie-*Lphn2pr*-mCherry-treated *Pc-Lphn2*$^{fl/fl}$ mice (referred to as AAV-ie-*Lphn2pr* mice). An antibody against the N-terminal (551-600 residues) of LPHN2 was used, which revealed the full-length LPHN2 (~170 kDa) and the N-terminal fragment of LPHN2 (~110 kDa) in the *Pc-Lphn2*$^{+/+}$ vestibules. In contrast, only a truncated version of LPHN2 (LPHN-GAIN, ~100 kDa) was detected in the vestibules of AAV-ie-LPHN2 mice. Representative blots from three independent experiments are shown (n = 3).

(c) Immunostaining of LPHN2 (green) with mCherry (red) in utricle whole mounts derived from *Pc-Lphn2*$^{fl/fl}$ mice treated with AAV-ie-LPHN2-mCherry or AAV-ie-*Lphn2pr*-mCherry (n = 3 mice per group). Scale bars: 50 μm and 10 μm for low and high magnification view, respectively.

(d, e) Representative current traces (**d**) and quantitative analysis (**e**) of fluid jet-stimulated MET responses of utricular hair cells derived from *Pc-Lphn2*$^{+/+}$, AAV-ie-*Lphn2pr* or AAV-ie-LPHN2 mice in the absence or presence of 50 nM D11 (n = 9, 10 and 10 for *Pc-Lphn2*$^{+/+}$, AAV-ie-*Lphn2pr* or AAV-ie-LPHN2 cells, respectively). Data are shown as mean ± SEM. ***P < 0.001; ns, no significant difference. Utricular hair cells treated with D11 compared with those treated with control vehicle. Data were statistically analyzed using paired two-sided Student's *t* test.

(f) Representative confocal (left) and STED (right) images of LPHN2 in utricular hair cells (n = 3 mice per group). Scale bars, 2 μm and 1 μm for low- and high-magnification views, respectively.
