# Supplementary material for: A force-sensitive adhesion GPCR is required for equilibrioception
Source: Cell Res. 2025 Feb 18;35(4):243–64. doi: 10.1038/s41422-025-01075-x (PMC11958651; doi:10.1038/s41422-025-01075-x)
Supplement: Supplementary file 12 — Supplementary TableS1 [file 41422_2025_1075_MOESM12_ESM.pdf]

Supplementary information, Table S1. The genotyping primer sequences for the mice used in the present study.

Name	5'-3' Sequence
Pou4f3-creER-F	TCCGAGTATGATCCTACCAGACCC
Pou4f3-creER-R	TCTCTTCCAGAGACTTCAGGGTGC
Adgrl2KO-F	CTACCGAGAAGTGTAAGGAAACCC
Adgrl2KO-R1	TGAAGGGACTTCTAAACTCAAACCT
Adgrl2KO-R2	TCATAGCTGATCATACACAGACCC
Adgrl3KO-F1	GGCCACTATGAGCTAGAGACTTTT
Adgrl3KO-R	CTCTCTCTTTCTGGCATGATCTGA
Adgrl3KO-F2	ACACCGAATCTCTTCAAGACTGAG
Gpr133KO-F1	CTTTGATCTCCTTCTTGGAGGCTTC
Gpr133KO-R	CAGTGTGAATCTTGTAGGTGGGTC
Gpr133KO-F2	CCGTTTCAACCTTCAGCTTCG
Gpr126Flox-F	CTCTTCACAGCCGGATTAGC
Gpr126Flox-R	TTGGGGAGCCTTTTTATCCT
Atoh1-cre-F	GCCTGCATTACCGGTCGATGC
Atoh1-cre-R	CAGGGTGTTATAAGCAATCCC
Vlgr1KO-P1	AGAGAGTAAGGACCCTCAGTTG
Vlgr1KO-P2	GAGTACTGCTAGCGGCGTAG
Vlgr1KO-P3	CTGACTTCTGTCATCCCAATG
Adgrl2Flox-F	AGCTTAAGAGAAGAGCATCTGGG
Adgrl2Flox-R	TGTGAACAAGATCCCCAGAACAG
Cib3KO-F	AGGTTTTGTACTGAGGAACTTGTC
Cib3KO-R1	CTGCTCTTCACTTATCCATACATTC
Cib3KO-R2	AAAGTGACCTTCCAGTAGTCCTC
Cib2KO-F	GGATTGTGGAGGCTTTCT
Cib2KO-R	ATGGCTCACAAGATGCTC
Tmc1KO-F1	GATGAACATTTTGGTACCCTTCTACTA
Tmc1KO-R1	CACACTTTGACACGTACAGTCTTTTAT
Tmc1KO-F2	TCTGAGCTTCTTAATCTCTGGTAGAAC
Tmc1KO-R2	ATACAGTCCTCTTCACATCCATGCT
Tmc2KO-F1	CGGTTCTTCTGTGGCATCTTACTT
Tmc2KO-R1	ACCAGGCAATTGACATGAATA
Tmc2KO-F2	CTGCCTTCTGGTTAGATCACTTCA
Tmc2KO-R2	GTGTTTTAAGTGTACCCACGGTCA
