# Supplementary material for: A force-sensitive adhesion GPCR is required for equilibrioception
Source: Cell Res. 2025 Feb 18;35(4):243–64. doi: 10.1038/s41422-025-01075-x (PMC11958651; doi:10.1038/s41422-025-01075-x)
Supplement: Supplementary file 13 — Supplementary TableS2 [file 41422_2025_1075_MOESM13_ESM.pdf]

Supplementary information, Table S2. The sequences of qRT-PCR primers used in the present study.

Name	5'-3' Sequence
Pou4f3-F	AATTCTCCAGCCTACACTCCG
Pou4f3-R	ATGGTATGGTAGGTGGCGTC
Lphn2-F	TGACTCAAAGGTGCAACAATCG
Lphn2-R	TAAGTTCCGGGACATGGATCA
Lphn3-F	AGTGACCGCAACACCATTCA
Lphn3-R	GAAGGCCGCCAAGAAGAAGA
Mouse-Actb-F	GACGAGGCCCAGAGCAAGAGAG
Mouse-Actb-R	ACGTACATGGCTGGGGTGTTG
Human- Actb-F	CATGTACGTTGCTATCCAGGC
Human- Actb-R	CTCCTTAATGTCACGCACGAT
Piezo2-F	ATGGCCTCAGAAGTGGTGTG
Piezo2-R	ATGTCCTTGCATCGTCGTTTT

References

1 Boyden, S. E., Desai, A., Cruse, G. *et al.* Vibratory Urticaria Associated with a Missense Variant in ADGRE2. *The New England journal of medicine* **374**, 656-663 (2016).

2 Karpus, O. N., Veninga, H., Hoek, R. M. *et al.* Shear stress-dependent downregulation of the adhesion-G protein-coupled receptor CD97 on circulating leukocytes upon contact with its ligand CD55. *Journal of immunology (Baltimore, Md. : 1950)* **190**, 3740-3748 (2013).

3 Yeung, J., Adili, R., Stringham, E. N. *et al.* GPR56/ADGRG1 is a platelet collagen-responsive GPCR and hemostatic sensor of shear force. *Proceedings of the National Academy of Sciences of the United States of America* **117**, 28275-28286 (2020).

4 Wilde, C., Fischer, L., Lede, V. *et al.* The constitutive activity of the adhesion GPCR GPR114/ADGRG5 is mediated by its tethered agonist. *FASEB journal : official publication of the Federation of American Societies for Experimental Biology* **30**, 666-673 (2016).

5 Petersen, S. C., Luo, R., Liebscher, I. *et al.* The adhesion GPCR GPR126 has distinct, domain-dependent functions in Schwann cell development mediated by interaction

with laminin-211. *Neuron* **85**, 755-769 (2015).

6 Scholz, N., Gehring, J., Guan, C. *et al.* The adhesion GPCR latrophilin/CIRL shapes mechanosensation. *Cell reports* **11**, 866-874 (2015).

7 Ping, Y. Q., Xiao, P., Yang, F. *et al.* Structural basis for the tethered peptide activation of adhesion GPCRs. *Nature* **604**, 763-770 (2022).

8 McMillan, D. R. & White, P. C. Loss of the transmembrane and cytoplasmic domains of the very large G-protein-coupled receptor-1 (VLGR1 or Mass1) causes audiogenic seizures in mice. *Molecular and cellular neurosciences* **26**, 322-329 (2004).
